# Profiling, Bioinformatic, and Functional Data on the Developing Olfactory/GnRH System Reveal Cellular and Molecular Pathways Essential for This Process and Potentially Relevant for the Kallmann Syndrome

**DOI:** 10.3389/fendo.2013.00203

**Published:** 2013-12-31

**Authors:** Giulia Garaffo, Paolo Provero, Ivan Molineris, Patrizia Pinciroli, Clelia Peano, Cristina Battaglia, Daniela Tomaiuolo, Talya Etzion, Yoav Gothilf, Massimo Santoro, Giorgio R. Merlo

**Affiliations:** ^1^Department of Molecular Biotechnology and Health Science, University of Torino, Torino, Italy; ^2^Department of Medical Biotechnology Translational Medicine (BIOMETRA), University of Milano, Milano, Italy; ^3^Institute of Biomedical Technology, National Research Council, ITB-CNR, Segrate, Italy; ^4^The George S. Wise Faculty of Life Sciences, Department of Neurobiology, Tel-Aviv University, Tel-Aviv, Israel

**Keywords:** olfactory development, GnRH neuron, Kallmann syndrome, extracellular matrix, transcription profiling, disease gene prediction

## Abstract

During embryonic development, immature neurons in the olfactory epithelium (OE) extend axons through the nasal mesenchyme, to contact projection neurons in the olfactory bulb. Axon navigation is accompanied by migration of the GnRH+ neurons, which enter the anterior forebrain and home in the septo-hypothalamic area. This process can be interrupted at various points and lead to the onset of the Kallmann syndrome (KS), a disorder characterized by anosmia and central hypogonadotropic hypogonadism. Several genes has been identified in human and mice that cause KS or a KS-like phenotype. In mice a set of transcription factors appears to be required for olfactory connectivity and GnRH neuron migration; thus we explored the transcriptional network underlying this developmental process by profiling the OE and the adjacent mesenchyme at three embryonic ages. We also profiled the OE from embryos null for *Dlx5*, a homeogene that causes a KS-like phenotype when deleted. We identified 20 interesting genes belonging to the following categories: (1) transmembrane adhesion/receptor, (2) axon-glia interaction, (3) scaffold/adapter for signaling, (4) synaptic proteins. We tested some of them in zebrafish embryos: the depletion of five (of six) *Dlx5* targets affected axonal extension and targeting, while three (of three) affected GnRH neuron position and neurite organization. Thus, we confirmed the importance of cell–cell and cell-matrix interactions and identified new molecules needed for olfactory connection and GnRH neuron migration. Using available and newly generated data, we predicted/prioritized putative KS-disease genes, by building conserved co-expression networks with all known disease genes in human and mouse. The results show the overall validity of approaches based on high-throughput data and predictive bioinformatics to identify genes potentially relevant for the molecular pathogenesis of KS. A number of candidate will be discussed, that should be tested in future mutation screens.

## Introduction

Central Hypogonadic Hypogonadism (CHH), is a heterogeneous genetic disorders characterized by absent or incomplete puberty, due to low circulating gonadotropins and sex steroids. Its mode of inheritance can be X-linked, autosomal dominant, or autosomal recessive, although unrelated sporadic cases occur more frequently (1). The disease is often associated with anosmia/hyposmia, in this case it is known as Kallmann Syndrome [KS, on-line Mendelian inheritance in man (OMIM) 308700], or with a normal sense of smell (normosmic CHH, or nCHH). These conditions are variably associated with non-reproductive phenotypes such as unilateral renal agenesis, skeletal abnormalities, midline malformations, or hearing loss. Neurological symptoms (including synkinesia of the hands, sensorineural deafness, eye-movement abnormalities, cerebellar ataxia, and gaze-evoked horizontal nystagmus) may also occur depending on the specific mode of inheritance (2).

Mutations affecting a large number of unrelated genes have been linked to the onset of KS/nCHH, currently including *Anosmin1* (*KAL1), Fibroblast Growth Factor Receptor-1* (*FGFR1*), *Fibroblast Growth Factor 8* (*FGF8*), *GnRH receptor* (*GNRH-R*), *Nasal Embryonic LHRH Factor (NELF), Kisspeptin (KISS1)*; *Kisspeptin Receptor (KISS-R)/G-protein-Coupled Receptor 54 (GPR54), Prokineticin-2 (PROK-2), Prokineticin Receptor-2 (PROKR2), Chromodomain Helicase DNA-binding Protein 7 (CHD7), Neurokinin-B (TAC3), Neurokinin-B Receptor (TAC3R), Heparan Sulfate 6-O-SulphoTransferase 1 (HS6ST1), SOX10, Semaphorin-3A* (*SEMA3A*), and five novel genes, members of the “FGF8-synexpressome” (1–8). In addition, several mouse models of targeted gene disruption have been shown to exhibit a KS-like phenotype (6, 9–18).

Despite the number of genes mutated in KS/nCHH, the majority of patients (>60%) do not harbor mutations in known disease genes, thus it is expected that many additional disease loci remain to be identified. In addition, the mutations found in KS/nCHH patients, once thought to act alone, are now recognized as cooperating mutations, and in fact in some cases a bi-genic or oligo-genic origin of these disease has been reported, with specific genotype/phenotype correlations (19–22). These findings open questions on the actual prevalence of single and combined mutations, the functional cooperation between them, and the possibility to use these information for accurate prognostic evaluations.

Kallmann syndrome is righteously considered a developmental disease. During embryonic development the GnRH neurons originate in the primitive olfactory area, migrate along the extending axons from the olfactory epithelium (OE) and the vomero-nasal organ (VNO), reach the anterior-basal forebrain and home in the septo-hypothalamic region of the adult brain (23–28). The association of the olfactory axons with the immature GnRH neurons, hence their ability to migrate and reach the hypothalamus, is an ancient and highly conserved developmental process, justified by the fact that it is essential for puberty and reproduction, in addition to neuro-modulatory functions (27, 29, 30). Not surprisingly this process is governed by a large set of molecular cues. Several studies have identified specific signaling molecules and their cognate receptors, as well as adhesion molecules, axon-glia and axon-matrix molecules play a role in guiding the axons to the correct position and consent the penetration of the basement membrane and the brain parenchyma (31–40). For instance, the semaphorin co-receptor Nrp1 is expressed by extending axon and GnRH neurons, and mediates the guiding functions of Sema3a, expressed in the nasal mesenchyme (10, 11). FGF8 has been shown to act as survival factor for olfactory and migrating GnRH neurons, which express its receptor FGFR1 (41–43), and both genes are mutated in a subset of KS/nCHH patients. To further complicate the picture, a cell population on the surface of the OB interacts with incoming axons, GnRH neurons and the CNS, and provide key signals for basement membrane fenestration, hence axon connectivity (44).

Due to the close relationship of olfactory axon elongation/connectivity and GnRH migration that occurs during embryonic development, the GnRH neuronal migration is strictly dependent on the integrity and connectivity of the olfactory pathway (30). A premature termination or mislocalization of olfactory axons results in impaired odor perception and GnRH homing. Thus, defects in olfactory development and/or GnRH neuron migration are considered the main primary cause of KS. The genetic findings summarized above have revealed much about the abnormalities that can befall both the development of the olfactory sensory system and GnRH neuron ontogenesis, including their differentiation, migration, maturation, circuit formation, and senescence.

Experimentally, animal models with altered olfactory and GnRH development are becoming available, including mouse, Zebrafish, and Medaka. The zebrafish embryo is ideal for developmental genetic studies, and the depletion of *anosmin-1a* leads to altered olfactory development and a KS-like phenotype (45, 46). In mice, several mutant strains display a phenotype that closely resemble KS/nCHH, including mouse mutant for *Dlx5* (14, 16, 47), *Emx2* (18), *Klf7* (13), *FezF1* (17, 48), *Six1* (12), *Prok2* and its receptor *Prokr2* (6, 15), *Lhx2* (9), *Ebf2* (49), *Nrp1* and *Sema3a* (10, 11). Notably, 7 of these (*Dlx5, Emx2, Klf7, Six1, FezF1, Ebf2*, and *Lhx2*) code for unrelated transcription factors, thus it can be postulated the existence of transcription regulatory networks, yet to be uncovered, that sustain olfactory development and connectivity, consent migration of the GnRH neurons and may contribute to the onset of KS/nCHH when altered. Furthermore, it is increasingly recognized that biological processes are governed and regulated by regulatory modules and networks of molecular interaction, not limited to protein-coding genes, rather then simplistically by individual genes.

To advance in our knowledge on the molecular regulation of axon extension/connectivity and GnRH neuron migration, in the present study we adopted a strategy based on the generation of transcriptome-wide profile data, combined with bioinformatic analyses and meta-analyses. In addition to the normal olfactory tissue we have also included one of the mouse models of KS, i.e., the *Dlx5* null (14, 16, 47). We then used transgenic Zebrafishes to image the olfactory axons and the GnRH neurons, and use these to establish the function of Dlx5 targets for olfactory axon extension/contact and on GnRH neuron migration and neurite extension. The results confirm a role for *Dlx5* and *FGFR1*, and indicate *Lrrn1* and *Lingo2* as novel players for olfactory axon organization and for GnRH neuron migration. Finally, we applied a gene prediction algorithm based on conserved co-expression networks, on all known human and mouse KS-causing genes. We predict a set of best candidates for causing, con-causing, or modifying the KS/nCHH phenotype.

## Materials and Methods

### Mice null for Dlx5

Mice with targeted disruption of *Dlx5* have been previously reported (50). The null allele, denominated *Dlx5^lacZ^*, allows for detection of the *Dlx5*-expressing cells by staining for β-galactosidase (β-gal) expression. The olfactory phenotype has been previously characterized (14, 16, 47). To obtain the WT samples, only WT males and females were crossed. To obtain *Dlx5* mutant samples, *Dlx5*^+/−^ (heterozygous) males and females were crossed; the progeny showed the expected Mendelian ratios of genotypes^+/+^, *Dlx5*^+/−^ and *Dlx5^−^*^/−^. Pregnant females were sacrificed at the chosen embryonic age by cervical dislocation. The day of the vaginal plug was considered E0.5. All animal procedures were approved by the Ethical Committee of the University of Torino, and by the Italian Ministry of Health.

### Tissue collection from mouse embryos

Embryos were collected clean of extra-embryonic tissues (used for genotyping) by manual dissection, transferred in RNAse-free PBS, and further dissected to separate the head. This was then included in 3% low-melting agarose in PBS, let harden and sectioned by vibratome (250 μm). Sections were manually dissected in cold PBS, with fine pins, to collect the OE or the VNO epithelia, or alternatively to collect the adjacent mesenchyme (Figure S1 in Supplementary Material). The excised tissues were individually collected in RNA-later (Ambion) and stored at −20°C until extraction. Following genotyping, samples of the same genotype were pooled. For the *Dlx5* mutant tissues, the entire epithelial lining of the nasal cavity was collected, since it was not possible to discriminate the OE vs. the respiratory epithelium.

### RNA extraction, labeling, and hybridization on mouse exon-specific arrays

At least 15 embryos were used for each developmental age, the collected tissues were pooled in three independent biological samples, used to extract total RNA with the Trizol (Invitrogen).

After extraction, RNA samples were quantified using a NanoDrop spectrophotometer (NanoDrop Technologies), the integrity of RNA molecules was assessed by capillary electrophoresis on a Agilent Bioanalyzer (Agilent), and found to have a RIN (RNA Integrity Number) value >5. One microgram of each total RNA sample (in triplicate) was processed using the Affymetrix platform’s instruments, following the GeneChip Whole Transcript Sense Target Labeling procedure, according to instructions. Ribosomal RNA was depleted using the RiboMinus kit (Invitrogen), cDNA was synthesized with random primers coupled with the T7 Promoter sequence, using SuperScript II for first-strand synthesis, and DNA Polymerase I for second-strand synthesis. The cDNA was and used as template for IVT amplification, using T7 polymerase. The amplificated products were used to synthesize single-stranded cDNAs, with the incorporation of dUTP, the products were fragmented by uracil-DNA-glycosylase (UDG) and apurinic/apyrimidinic endonuclease-1 (APE 1) treatment. Finally, 5.5 μg of fragmented cDNA samples were biotinylated with terminal deoxynucleotidyl transferase and used to hybridize on GeneChip^®^ Exon 1.0 ST Arrays (Affymetrix, Santa Clara, USA). The Chips were washed and stained with Streptavidin-phycoerithrin in the GeneChip Fluidic Station 450 and scanned with Affymetrix GeneChip^®^ Scanner 3000 7G.

### Analysis of microarray data

Quality control was performed using the Affymetrix Expression Console software^1^. All the experiments exhibited optimal quality controls and correctly clustered in the right sample groups; they were thus all included in the analysis. Normalization and probeset summarization steps were performed with RMA, within the OneChannelGUI package (51) included in Bioconductor (52), separately for each pairwise comparison including the six relevant arrays (three biological replicates per condition). Differentially expressed genes (DEG) for each pairwise comparison were obtained with Rank Products (53), adopting a 0.05 false discovery rate (adj. *p*-value ≤0.05).

### Softwares and databases

For preliminary Gene Ontology (G.O.) analyses we used DAVID^2^ and KEGG^3^. For improved categorization and visualization, we used ClueGO (54). For the time course analysis we used default parameters. For the analysis of down-regulated DEGs in the *Dlx5^−^*^/−^ samples we relaxed the analysis by using a cutoff of 0.001 on nominal enrichment *p*-value. For embryonic expression of RefSeq genes we used the two on-line *in situ* hybridization databases GenePaint^4^ and Eurexpress^5^. For the position weight matrix (PWM) we used the JASPAR database. Tissue-specific conserved co-expression networks were obtained with the TS-CoExp Browser^6^ (55).

We also used the following web resources: Ensembl Genome Browser^7^, UCSC Genome Browser^8^, RefSeq^9^, Mouse Genome Informatix^10^, OMIM^11^.

### Genome-wide prediction of Dlx binding sites and putative target genes

With the PWM of Dlx5 provided by JASPAR under accession PH0024.1 (56) Dlx5 sites were predicted by standard log-likelihood ratios, using as null model the nucleotide frequencies computed over the whole intergenic fraction of the mouse genome. We considered only those sites scoring 50% of the maximum possible score or better. We selected sites that are conserved in at least two (of eight vertebrate species). A site is defined as conserved with species S if it lies in a region of the mouse genome which is aligned with a region of the S genome and the aligned sequence in/S/is a site according to the same definition used for mouse sites. A ranked list of putative Dlx5 targets was obtained from the identified sites as described (57).

### Conserved co-expression network, and prediction/prioritization of putative disease genes

Tissue-specific conserved co-expression networks were obtained with the TS-CoExp Browser (see footnote text 6) (55, 58), based on 5188 human and 2310 mouse manually annotated microarray experiments. For disease prediction/prioritization we used a tool within the TS-CoExp Browser and the same approach based on conserved co-expression networks, but instead of using genes causing similar phenotypes, we used KS-disease genes as “reference” genes. These genes were selected based on documented mutations in KS patients (for human) or well described olfactory/GnRH embryonic phenotype recapitulating KS (mouse).

### Validation of array data by real-time qPCR

Tissue samples corresponding to WT and *Dlx5^−^*^/−^ OE were collected from embryos at the age E12.5, transferred in RNA-later in individual tubes and stored at −20°C. The genotype was determined on extra-embryonic tissues. Samples were pooled according to the genotype, collected in Trizol (Invitrogen), and used to extract total RNA according to the instructions. For Real-Time qPCR, 250 ng of total RNA was reverse-transcribed at 42°C for 50 min in the presence of 500 ng/μl random hexamers, 10 mM of each dNTPs, RNasin and Improm Reverse Transcriptase (Promega). Relative cDNA abundance was determined using the AB7900 System and the GoTaq qPCR Master Mix (Promega). Specific cDNAs were amplified using primers and probes designed according the Universal Probe Library system (UPS, Roche). Experiments were repeated at least twice on independent samples, every point was done in triplicate, results were normalized to the level of *TATA-binding protein* (TBP) and *GAPDH* mRNAs. Data analysis was performed with ABI software, version 2.1 (Applied Biosystems) using the comparative Cq method, calculated with the formula of the DDCq. For each primer-pair, the melting curves of the amplified products revealed a single peak. Primer sequences are provided (Table S1 in Supplementary Material).

### Zebrafish strains and gene knock-down in embryos

The following two strains were used for visualization of the olfactory axons: *OMP^2k^:gap-CFP^rw034^* and *TRPC2^4.5k^:gap-Venus^rw037^* (59–61), and were obtained from Drs. Nobuhiko Miyasaka and Yoshihiro Yoshihara (RIKEN Brain Science Inst., Japan). The fish strain *GnRH3:GFP* (62–64) was obtained from Dr. Y. Zohar (University of Maryland Biotechnology Institute, Baltimore, USA) and Dr. Y. Gothilf (Life Sciences, Tel-Aviv University, Israel). Adult fishes were maintained, bred and genotyped according to standard procedures, kept under a 14 h-light and 10 h-dark photoperiod at 28°C. Allelic transmission followed the expected Mendelian ratios. Following fertilization, 1-cell zygotes were collected and maintained in the presence of 0.003% 1-phenyl-2-thiourea (PTU) to prevent formation of melanin.

To down-modulate specific genes, we injected antisense morpholino oligos (MO) into zebrafish oocytes (65, 66). MO were designed either to block splicing at a specific exon-intron junction (GeneTool oligo design), and consequently lead to present of aberrant transcripts and frame-shifted translation, or to anneal to the ATG start codon and inhibit translation initiation. For *z-dlx5a* we combined two MOs: one annealing with the exon1-intron1 splice junction and leading to a premature Stop codon upstream of the homeodomain; the other annealing with the Start codon. Sequences and properties of all the MO are in Table S2 in Supplementary Material. Zygotes were collected at one-cell stage and injected under stereological examination with 4 ng of MO, in presence of Phenol Red for subsequent selection. From 48 to 72 h post fertilization (hpf) embryos were fixed with 4% PFA at 4°C ON, washed in PBS, and embedded in 4% low-melting agarose, 0.1% Tween-20. The apical portion of the head was manually dissected from the rest of embryo. Confocal microscopy analysis was performed using a Leica TCS SP5 (Leica Microsystems). The OMP:CFP+ and the Trpc2:Venus+ (YFP+) axons were viewed in a frontal plane, while the GnRH3:GFP+ neurons were viewed in a ventral plane. Images were acquired as Z-stacks of 1 μm thick optical sections. Digital micrographs images were contrast balanced and color matched using Photoshop7 (Adobe), cropped, rotated, and assembled into figures with QuarkXpress (Pantone).

## Results

### Genes differentially expressed during olfactory development

We set forth to generate expression profiles of the OE at key stages of its development, comprising the time of axonal connection. We selected three developmental stages, i.e., the Olfactory Placode (OPL) at E11.5, the OE at E12.5, and either the OE or the VNO at E14.5. Mouse Affymetrix GeneChip^®^ Exon 1.0 ST Arrays were used to analyze the gene expression profiles of the developing olfactory (neuro)epithelium (OE). Comparing the OE E12 vs. the OE at E11, with adj. *p*-value ≤0.05 and fold-change ≤−0.9 or ≥0.9, we found 29 up-regulated and 62 down-regulated genes. Comparing the OE at E14 vs. the OPL E11 we found 358 up-regulated and 17 down-regulated genes. Comparing the VNO E14 vs. the OPL E11 we found 459 up-regulated and 21 down-regulated genes.

A fraction of the DEGs might derive from mesenchymal cells present in the epithelial samples; as a matter of fact, epithelial cells do not easily detach from the basement membrane and mesenchymal cells inevitably tend to remain attached. A survey of the embryonic expression territory of the modulated genes using the on-line expression databases and www.genepaint.org and www.eurexpress.org showed that about 10% of the DEGs was indeed expressed in the nasal mesenchyme adjacent to the OE, and not in the OE or VNO proper. Thus, we decided to estimate the extent of mesenchymal contamination in the OPL, OE, and VNO samples, by collecting pure mesenchymal tissue adjacent to the OPL, OE, and VNO, at the same embryonic ages, and use the RNA extracted from these to quantitatively determine the mRNA abundance of “epithelial only” (*FoxJ1, Fmo2*, and *Ehf*) and “mesenchymal only” (*Sp7* and *Lect1*) genes, by Real-Time qPCR. In the same experiment we compared the samples of the OE (mixed epithelium and mesenchyme) with “pure mesenchyme” samples at the same embryonic age. The results indicate that the abundance of a mesenchymal RNA in the OE samples is roughly 15% that of the pure mesenchyme samples, thus we assumed that the contribution of MES in the EPI samples is 15% (Figure S2 in Supplementary Material).

At the same time, using the GeneChip^®^ Exon 1.0 ST Arrays and the same hybridization procedure and statistical analyses used before, we generated profiling data from the MES samples collected from wild-type embryos at E11.5, E12.5, and E14.5. At the age E14.5 the samples were collected adjacent to the OE or adjacent to the VNO, according to their anatomical position, and maintained separated. This effort was undertaken to: (1) explore the global changes of expression that underlie interaction between the OE and the MES, (2) carry out a subtraction step on the raw EPI data, to generate cleaner OE data.

By comparing the MES samples at E12 vs. E11 we found 118 up-regulated and 17 down-regulated genes; comparing the samples OE at E14 vs. OPL E11 we detected 284 up-regulated and 41 down-regulated genes, while comparing the VNO at E14 vs. OPL E11 we detected 293 up-regulated and 35 down-regulated genes (the non-annotated probes are not included). Then we subtracted the estimated expression of MES genes (15%) from the raw expression data, applying this general formula to all genes present and expressed:

$$E_{i}^{c}\left(g\right)=E_{i}\left(g\right)-F\times M\left(g\right)$$

where *E_i_*(*g*) is the expression of gene g in the i-th replicate of the EPI dataset and *M*(*g*) is the expression in the MES dataset, averaged over all replicates. *F* is the estimated mesenchymal fraction, equal to 0.15. Choosing *F* to be equal to 0.1 or 0.2 did not significantly alter the results. With this calculation we created a subtracted and corrected dataset with expression values more indicative of the sole EPI expression. Comparing the corrected EPI samples at E12 vs. E11 we found 9 up-regulated genes and 57 down-regulated; comparing the samples OE at E14 vs. OPL E11 we detected 250 up-regulated and 19 down-regulated genes, while comparing the VNO E14 vs. OPL E11 we detected 347 up-regulated genes and 14 down-regulated (the non-annotated probes and the OR genes are not counted). After the subtraction, a number of genes reached a “no expression” level. We assume that this is due mainly to the fact that their differential expression was relative to the MES. We examined how many of the genes that disappeared from the raw list are up-regulated in the MES samples, and detected highly significant enrichments (*p* < 4e–12).

The corrected lists of EPI DEGs up- and down-modulated in the OE E14 vs. OPL E11 are reported in Tables S3 and S4 in Supplementary Material, respectively, while the lists of DEGs up- and down-modulated in the VNO E14 vs. OP E11 are in Tables S5 and S6 in Supplementary Material, respectively. In the OE we find genes expected to be associated or to play a role in neuronal differentiation and/or olfactory development, such as *NeuroD, OMP, Peripherin, NCAM2, Claudins, Keratins*, and *Lhx2* (a gene causing a KS-like phenotype in the mouse) (9). In addition we find a set of olfactory receptor (OR) genes, as expected (Table S7 in Supplementary Material).

**Figure 1:**
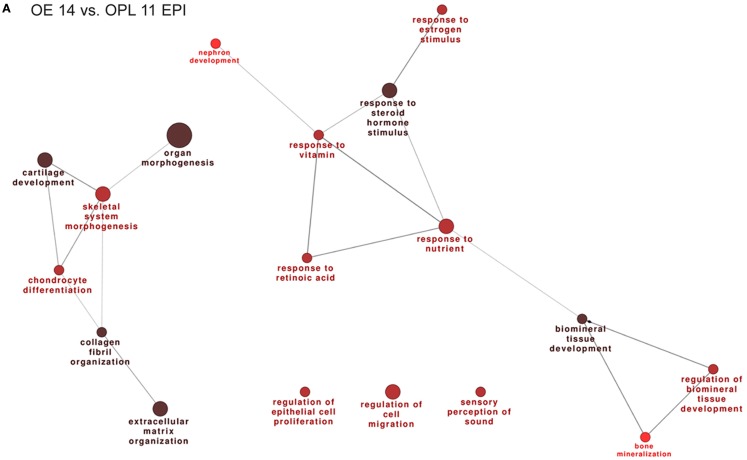

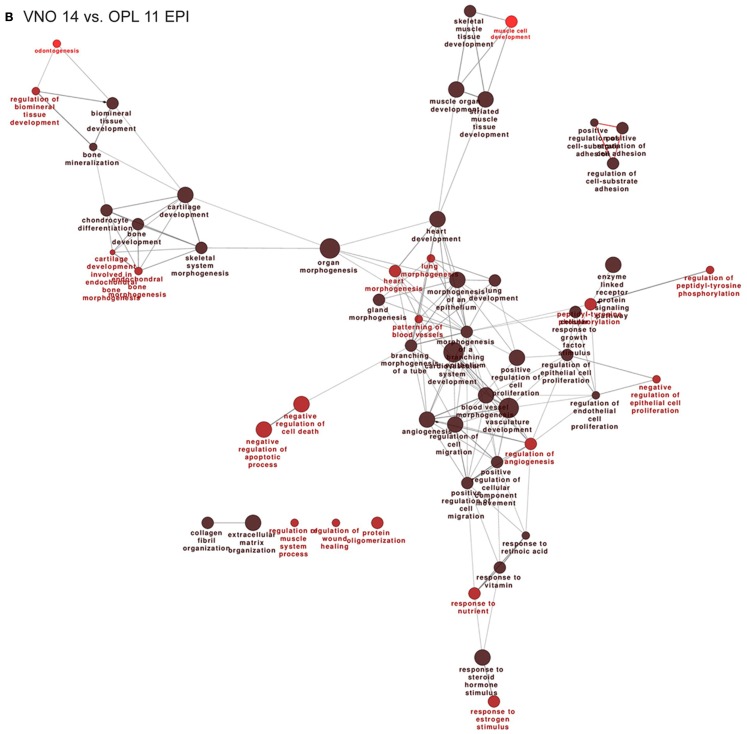
**Functional categorization of genes differentially expressed during normal OE and VNO development**. Significantly over-represented functional categories of DEGs detected in the (corrected) EPI samples, comparing OE 14.5 vs. OPL 11.5 **(A)** or comparing the VNO 14.5 vs. OPL 11.5 **(B)** were organized them in networks using ClueGO. Only the results of the up-regulated DEGs are shown. Circles represent over-represented categories, lines indicate related categories.

Next we carried out functional categorization analyses on the genes up-regulated in the OE, to identify enriched functional categories, using the Gene Ontology-based ClueGo tool (54, 67). Since this analysis could be biased by the OR genes, which are numerous (about 1000 in the mouse genome) and belong to a single category, we masked the OR genes. The results are shown in Figure 1A. From the comparisons OE 14 vs. OPL 11 we detect: regulation of epithelial cell proliferation, regulation of cell migration, regulation of extracellular matrix organization, and various categories of response to signals.

In the VNO, we find several genes expected to be associated or to play a role in VNO development, such as *NeuroD, OMP, Lhx2, Peripherin, Claudins, Keratins, EphA3, Neuropilin1, Lamininβ3, Lhx2* (a Kallmann gene in the mouse), and *Dcx*. In addition we find several OR genes, as expected (Table S8 in Supplementary Material). We carried out functional categorization on the genes up-regulated in the VNO, after masking the differentially expressed OR genes, and detected the over-represented classes shown in Figure 1B. Focusing on the comparisons E14 vs. E11, we detect: regulation of epithelial cell proliferation, regulation of cell migration, regulation of cell adhesion, gland and epithelium morphogenesis, cartilage development, bone development, extracellular matrix organization, and various categories of response to signals.

### DEGs in olfactory-associated MES, during development

**Figure 2:**
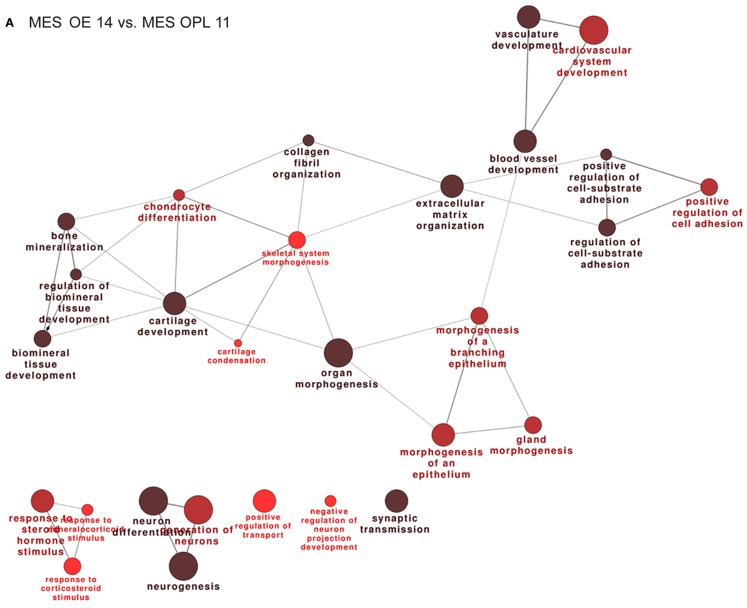

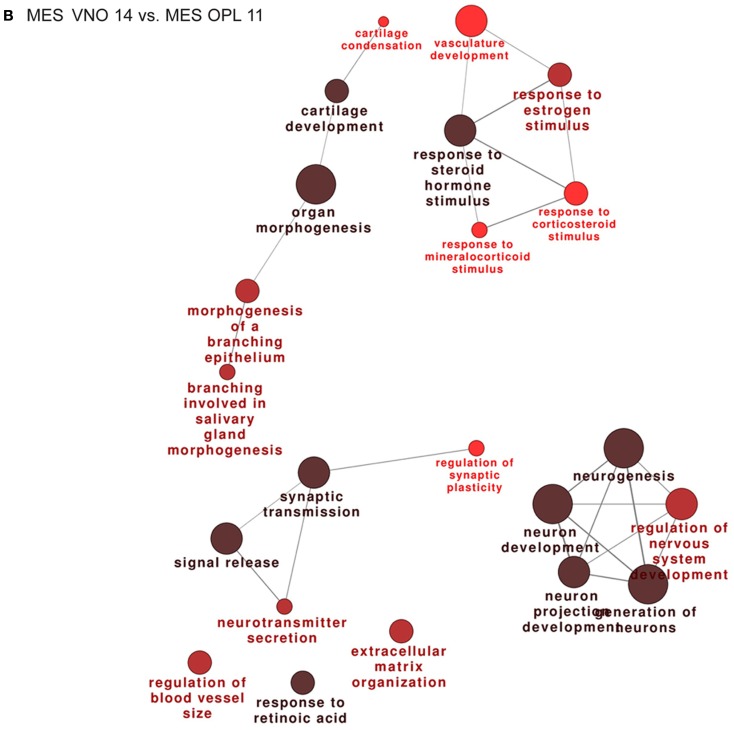
**Functional categorization of genes differentially expressed in the mesenchyme adjacent the OE and VNO, during development**. Significantly over-represented functional categories of the DEGs detected in the mesenchymal tissue associated with the OE 14.5 vs. OPL 11.5 **(A)** or associated with the VNO 14.5 vs. OPL 11.5 **(B)** were organized in networks using ClueGO. Only the results of the up-regulated DEGs are shown.

We then compared the profiles of the MES samples across the developmental ages E11.5–E12.5–E14.5. The full lists of up- and down-modulated DEGs relative to the OE are provided in Tables S9 and S10 in Supplementary Material, respectively, while the full lists of up- and down-regulated DEGs relative to the VNO are provided in Tables S11 and S12 in Supplementary Material, respectively. We recognized genes playing a role in cell–cell communication, signaling, matrix remodeling, etc. such as *Integrins, Contactins, Matrillins, Tenascin, Collagens, MMPs, Adams, Lectin Galactose Binding 9, Elastin, FGF7, FGF12, Sfrp2, Sfrp4, Sema3D, Sema3C, Nrp1, Wnt2, Bmp5, Follistatin*. We also found some neuronal genes, likely due to a minimal presence of olfactory neuron in the MES sample and to the presence of migratory GnRH neurons in the E14 sample, minimal in the E11 sample. Functional categorization on these DEGs detected an enrichment of the following categories: extracellular matrix organization, cell-substrate adhesion, cartilage and bone development, organ morphogenesis, response to signals, and some neuronal categories (Figures 2A,B).

### Profiling of the Dlx5^*−*/−^ vs. wild-type OE

**Figure 3:**
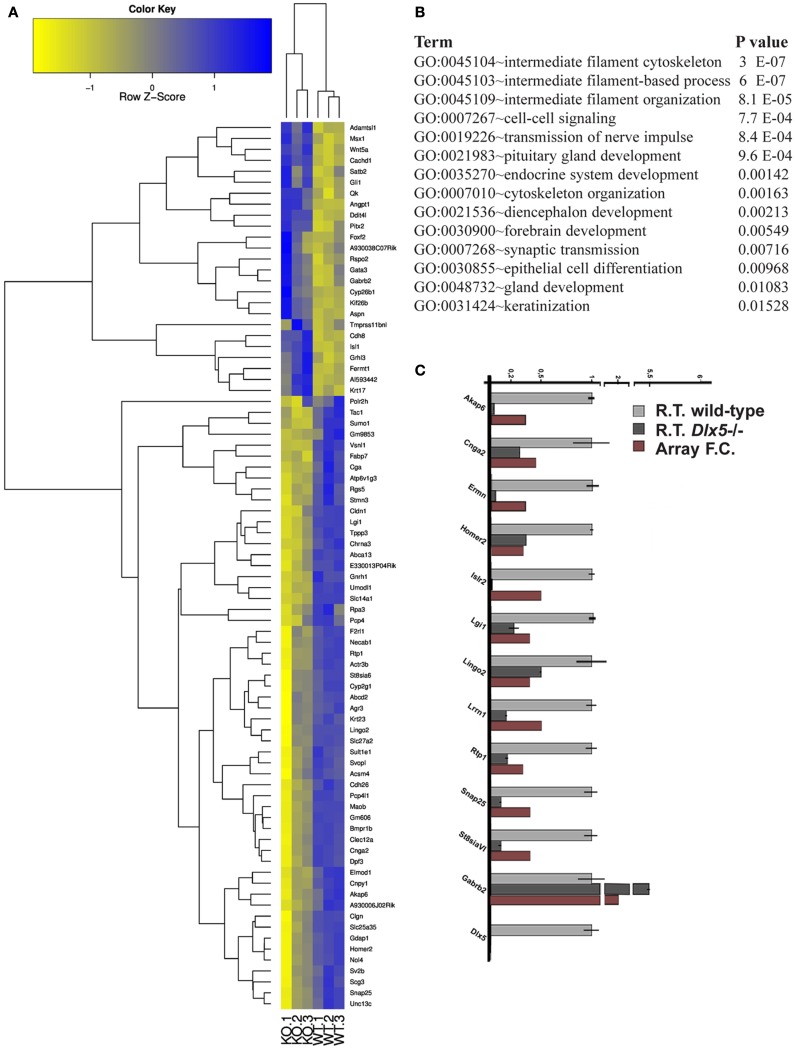
**Genes differentially expressed in the *Dlx5*^−^*^/−^ O*E vs. wild-type OE**. **(A)** Heatmap and cluster analyses on the MicroArray data, after elimination of non-annotated genes and of the OR genes. The triplicate *Dlx5^−^*^/−^ (KO) and wild-type (WT) samples are indicated at the bottom. **(B)** Gene Ontology to detect over-represented functional categories. **(C)** Validation by Real-Time qPCR on selected down-regulated genes, done on independently collected samples. The abundance in the wild-type tissue is set = 1. For each gene, the fold-change determined by MicroArray hybridization is also reported.

The *Dlx5^−^*^/−^ mutant mice represent a fully penetrant model of KS (14, 16, 47). Triplicates of the OE and VNO tissues were collected from WT and *Dlx5^−^*^/−^ embryos at the age E12.5, total RNA was extracted and hybridized on the GeneChip^®^ Exon 1.0 ST Arrays. Using the indicated statistical parameters (see Materials and Methods) we detected 121 down- and 25 up-regulated genes in the *Dlx5^−^*^/−^ OE vs. the WT, not counting the non-annotated probes and the OR genes (Figure 3A; Table S13 in Supplementary Material). Again, the OR genes were removed (provided in Table S14 in Supplementary Material) prior to conducting functional categorization analysis by G.O. We detected: intermediate filament/cytoskeletal organization, endocrine system development, forebrain development, cell–cell signaling, and epithelial cell differentiation (Figure 3B).

We carried out a technical validation of the microarray results, by selecting 12 down- and 4 up-regulated DEGs and quantifying their expression on independently collected samples, by Real-Time qPCR. Of these DEGs, 11 down- and 2 up-regulated were confirmed (Figure 3C).

Next we verified whether the identified DEGs are expressed in the OE, in the adjacent mesenchyme, or in both, by consulting the on-line expression databases GenePaint and Eurexpress. We classified DEGs as either not expressed (−), expressed in the neurepithelium (NEp), expressed in the mesenchyme (Mes), expressed in the respiratory epithelium (Res) or ubiquitously expressed (Ub). We assumed that the OR were all expressed in NEp, and in any case they were excluded. Among the 72 down-modulated DEGs considered, 50 (69%) have a NEp expression, 14 (19%) are not expressed in the OE, 4 (5%) have a Mes expression, 3 (4%) have a Res expression, and 2 (3%) are ubiquitously expressed. Thus, conclude that the majority of down-modulated genes are expressed in the OE.

Then we examined whether the DEGs were differentially expressed also in other mouse tissues upon loss of *Dlx5*, specifically the inner ear and the pharyngeal arches (68, 69). No common DEG was found, indicating that Dlx5 targets are strongly tissue-specific. Next we examined whether the olfactory DEGs we detected were also differentially expressed in other published mouse models of KS, i.e., the *Klf7* and the *Emx2* mutants (70, 71). Three genes were found commonly differentially expressed the three models, namely *stathmin-like 3, synaptotagmin 1*, and *calmegin*, all expressed in the embryonic OE. Fifty genes were in common between Dlx5 and Emx2 datasets, seven were in common between *Emx2* and *Klf7*, and one was in common between Dlx5 and Klf7. However it should be noted that the profiles of the *Emx2* and *Klf7* mutants were obtained from the OB and not the OE.

The DEGs up-regulated in the absence of *Dlx5* are enriched in generic terms: biosynthesis, metabolic processes, morphogenesis. Of the 27 DEGs considered, 14 (52%) are expressed in the Mes, 6 (22%) are not expressed in the OE/VNO, 3 (11%) have a Res expression, 2 (7.5%) are expressed in the OE, and 2 (7.5%) are ubiquitously expressed. Thus, we conclude that most of the up-regulated DEGs are not expressed in the OE. Since the Dlx proteins are generally considered transcription activators (72, 73), the interest in these DEGs is low and they were not further considered.

### Genome-wide prediction of Dlx5 binding sites and transcriptional targets

Using the consensus PWM for Dlx5 (56) (Figure S3 in Supplementary Material) we screened conserved regions of the vertebrate genome and detected putative Dlx5 binding sites. We attributed to each site a score that reflects the number of species in which the site is conserved. We then associated the sites to an associated Refseq transcripts and found 3,426 RefSeq targets, corresponding to 2,683 unique Entrez-IDs [see Materials and Methods, and Ref. (57)]. The top scoring RefSeq are reported in Table S15 in Supplementary Material. We then categorized the predicted Dlx5 targets by ClueGO (54), and detected an enrichment categories such as neuronal differentiation, brain development, etc. as expected [there is ample literature on this; see Ref. (73)].

We then intersected the best predicted Dlx5 targets (having at least one binding site conserved in at least three mammalian species, and located <10 kb from the TSS) with the list of DEGs obtained comparing *Dlx5^−^*^/−^ vs. WT OE, and we found that 16% of the down-regulated DEGs (19/121; *p* = 0.0003) were indeed predicted target of *Dlx5*, while 40% of up-regulated DEGs (9/21; *p* = 0.00019) were predicted targets. In both cases statistical significance was reached. This suggests that the prediction algorithm we have used is sensitive and sound. To restrict the number of candidate genes we intersected the profile datasets with: (a) embryonic expression databases, (b) conserved co-regulations, (c) predicted Dlx5 sites and target RefSeq, (d) data from published literature, in order to assign a score value to each DEG (Tables 1A–E). The expression of these putative *Dlx5* targets in the embryonic OE and nasal region, by *in situ* hybridization (see footnote text 4) is reported in Figure S5 in Supplementary Material. Some of the most functionally relevant genes are briefly described below:

**Table 1:** **Best *Dlx5* target gene, selected combining the profiling results with PWM-based site prediction and embryonic expression**.

Gene title	Gene symbol	log2.FC.	Dlx site	Express	Score	Notes
**(A) SURFACE RECEPTORS/ADHESION MOLECULES OR MODIFIERS**						
Leucine-rich repeat and Ig domain containing 2	*Lingo2*	−1.3995539	+	N Ep	5	Structure similar to other Receptor Tyrosine Kinases, such as Trk. Associated to higher risk of tremor and Parkinson. Lingo1 is a component of the NOGO-66 receptor and may play a role in neurite outgrowth and oligodendrocyte differentiation
Leucine-rich repeat LGI family, member 1	*Lgi1*	−1.355411	+	N Ep	5	Secreted molecule of the SLIT family, promotes formation of stress fibers. Inhibits cell movement and invasion. Enhances growth of neuronal processes on myelin-based substrates. Its receptor forms complexes with Adam22
Leucine-rich repeat protein 1, neuronal	*Lrrn1*	−1.032383	+	N Ep	5	Transmembrane protein of unclear function. Regulates neurite growth
Ig superfamily containing leucine-rich repeat 2	*Islr2*	−0.9967503	+	Not/migr cell	4	Also known as Linx, could be a Receptor Tyrosine Kinase evolutionarily related to Trk receptor. Modulates axon extension and guidance
ST8 α-*N*-acetyl-neuraminide α-2,8-sialyltransferase VI	*St8siaVI*	−1.3472121	+	N Ep	4	Sialo-transferase expressed by neurons, essential for surface functions during neurite growth and neuronal migration
**(B) SCAFFOLD INTRACELLULAR PROTEINS**						
A kinase anchor protein 6	*Akap6*	−1.5186358	+	N Ep	5	Protein Kinase A-anchoring proteins. Serves as scaffold to bring together PKA and PDE and coordinate the timing and intracellular localization of cAMP signaling. Also binds to- and modulates-signaling through ERK, MAPK, and PP2A
Dual adaptor for phosphotyrosine and 3-phosphoinositides 1	*Dapp1*	−1.2094534		N Ep	4	Signaling adapter molecule, coordinates timing and location of signaling by PIP3 and PIP2 with that of ERK. Also binds F-actin and Rac
RIKEN cDNA 9330120H11 gene	*9330120H11Rik*	−1.1589186		N Ep	4	Also known as HOMER 2, present at post-synaptic density, involved in receptor clustering, trafficking, and in calcium homeostasis
**(C) SYNAPTIC PROTEINS**						
Synaptosomal-associated protein 25	*Snap25*	−1.3481758	+	N Ep	5	Controls membrane trafficking and fusion at the growth cone and at the synapse. Implicated in neuroblast migration and neuritogenesis during development. Forms complex with p140CAP which also binds to p130 CAS
γ-Aminobutyric acid (GABA) A receptor, subunit β 2	*Gabrb2*	1.0063619	+	N Ep	5	Receptor subunit for GABA. GABA-b receptors mediate signals inhibitory for olfactory axon elongation
Receptor transporter protein 1	*Rtp1*	−1.6548021		N Ep	4	Chaperon, required for the efficient translocation of OR molecules to the membrane. Interacts with the OR and with Homer
RIKEN cDNA 9330120H11 gene	*9330120H11Rik*	−1.1589186		N Ep	4	Also known as *Homer2*, present at post-synaptic density, involved in regulation of calcium fluxes and homeostasis
**(D) AXON-GLIA INTERACTION PROTEINS**						
Fatty acid binding protein 7, brain	*Fabp7*	−1.9620307		N Ep-Gliale	4	Known as BLBP in human. Controls surface functions that are required for axon-Schwann cell interaction. May be involved in peripheral axon elongation and regeneration
Ermin, ERM-like protein	*Ermn*	−1.5033487		N Ep/Sust cell	4	Also known as Juxtanodin. Expressed in sustentacular cells, binds to F-actin and stabilizes the actin cytoskeleton. In the CNS promotes myelination
Ganglioside-induced differentiation-associated-protein 1	*Gdap1*	−1.1935825	+	N Ep/Res	4	Involved in the Charcot-Marie tooth disease, in particular those forms with axonal deficits. Cellular function unclear
UDP Galactosyltransferase 8A	*Ugt8a*	−1.1139671	+		4	Important for the biosynthesis of galacto-lipids and in myelin formation
**(E) CALCIUM-REGULATION**						
Cyclic nucleotide gated channel α 2	*Cnga2*	−1.129349	+	N Ep	5	Regulate axon extension and glomerular formation. KO mice have behavioral defects possibly linked to olfactory functions
Visinin-like 1	*Vsnl1*	−1.3860936	+	N Ep	4	Also known as GP2. Calcium-regulated guanylate cyclase transduction system. Play a role in adaptation. Inhibits the formation of cAMP. May affect dendrite and growth cone arborization

*Genes are sub-divided in five general categories (A–E)*.

*Lrrn1* codes for a transmembrane protein related to Drosophila TRN/CAPS proteins, known play a role in neuromuscular target recognition, and to mediate interactions between incoming axons and the targets, possibly via homophilic adhesion. *Lrrn1* is expressed in the mouse embryonic OE.

*Lingo2* (also known as *Lrrn6c*) codes for a transmembrane protein, expressed in the OE and in the ventricular region of the embryonic forebrain. Lingo proteins interact with the NOGO receptor and are able to modulate the NOGO pathway (74), however their precise functions are poorly known.

*Lgi1* codes for a leucine-rich repeat secreted molecule of the SLIT family, involved in growth of neuronal processes on myelin substrates (75, 76).

*St8siaVI* is expressed by olfactory neurons and might be implicated in polysialylation the N-CAM to confer anti-adhesive properties to neuronal surfaces (77–79).

*Homer2* codes for a protein present at post-synaptic density, likely to be involved in receptor clustering and trafficking, as well as calcium homeostasis (80). Recently, a role of Homer2 in tuning the activity of G protein-coupled receptors (such as ORs) has been reported (81). *Homer2* is expressed in the OE of the mouse embryo, however its function is unknown.

### Testing Dlx5, Dlx5 targets, and KS genes in zebrafish strains: The olfactory axons

The development of olfactory system is well conserved during vertebrate evolution (27, 35, 82) and consists of two independent components: the main OE for detecting chemical compounds (odorants) and the VNO-accessory system for detecting pheromones. Fishes and primates lack a VNO organ and present only one olfactory organ, the OE (83). Within the OE of the fish, all ORNs project their axons to the OB – at different region in a mutually exclusive manner (60) – but display distinct properties with respect to their morphology, relative position in the OE, and molecular expression. The ciliated OSNs with long dendrites are situated in the deep layer of the OE, whereas microvillous ORNs with short dendrites are located in the superficial layer. The ciliated and microvillous ORNs are reported to express OR-type and V2R-type receptors, respectively (84, 85).

We opted to use *Danio rerio* (zebrafish) as a model to functionally examine *in vivo* the identified DEGs for their role in olfactory/GnRH development. We used two transgenic zebrafish strains expressing distinct fluorescent proteins in the fish olfactory neurons (59–61). In one strain the *CFP* reporter is expressed under the control of *OMP* promoter, which marks the majority of basal-layer ORN, projecting their axons to the dorsal OB. In the other strain, the *Venus* (YFP) reporter is expressed under the control of the *Trpc2* promoter, which marks a sub-population of apical-layer ORN, projecting to the ventro-lateral OB (scheme in Figure S4 in Supplementary Material) (60). The CFP+ and the Venus+ (YFP+) neurons are thought to correspond, respectively, to the OE and VNO receptors of the mammalian system. Since the reporter fluorescent proteins are efficiently translocated in the ORN axons, these two strains visualize the peripheral olfactory pathway.

**Figure 4:**
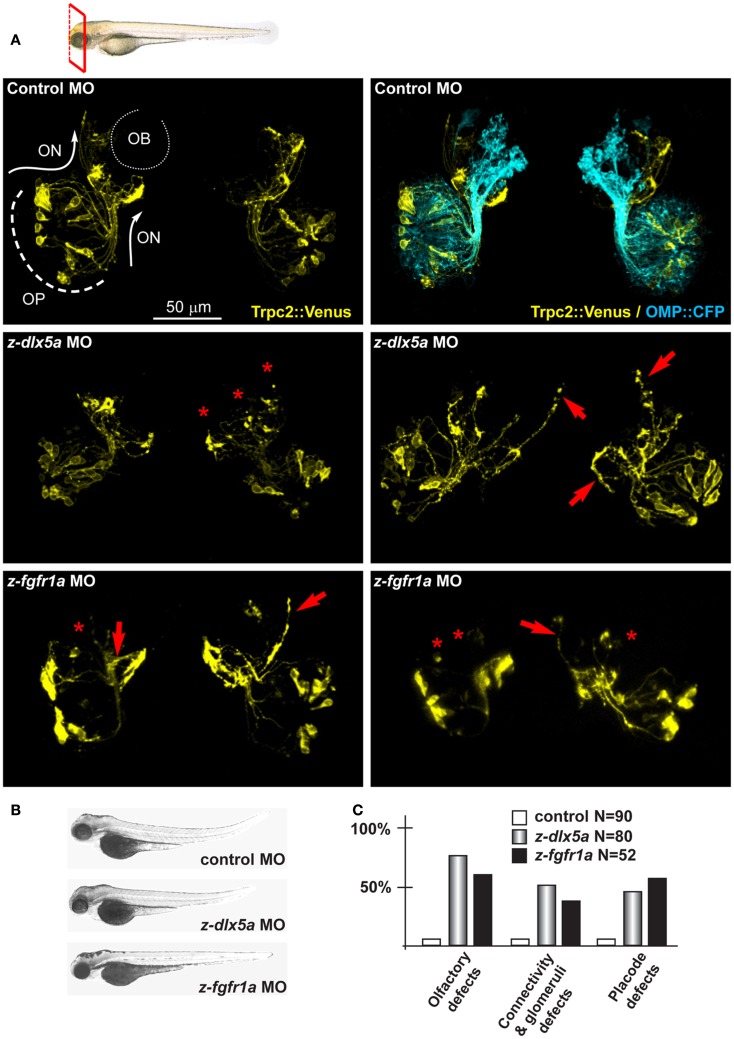
**Depletion of endogenous *z-dlx5a* and *z-fgfr1a* in zebrafish embryos, to image the olfactory axons**. **(A)** Micrographs of *Trpc2:Venus* (YFP, yellow fluorescence) and *OMP:CFP* (cyan fluorescence) fish embryos injected with a control MO (top panels), injected with anti-*z-dlx5a* MO (middle panels) or injected with anti*-z-fgfr1a* MO (bottom panel). White arrows and lines indicate the normal axonal pathway in control embryos. Red asterisks indicate absence of glomeruli. Red arrows indicate altered axonal trajectories. **(B)** Whole-mount bright field micrographs of injected embryo, showing an overall normal embryonic morphology and growth rate in the injected embryos, compared to control injected ones. **(C)** Proportions of embryos showing either placode defects (OPL disorganization, altered neuron distribution), or connectivity/glomeruli defects (altered axon trajectory, altered fasciculation, reduced or absent glomeruli), or both, upon injection of control (open bars), anti-*z-dlx5a* (gray bars), or anti-*z-fgfr1a* (solid black bars) MOs.

We tested z-*fgfr1a*, the fish ortholog of mammalian *FGFR1*, to establish whether its depletion recapitulates the hallmarks of KS. Notably, mice hypomorphic for *FGF8* expression show distinctive signs of a KS phenotype, i.e., impaired migration of GnRH+ neurons and defects in olfactory development (41, 42). We injected *z-fgfr1a* MOs in 1-cell embryos of the *OMP:CFP* and the *Trpc2:Venus* strains, and 72 hpf we examined the number of fluorescent embryos, the organization of the OPL, the fasciculation, extension and glomeruli formation. In 61% (32/52) of the embryos we observed an altered morphogenesis of the OPL and an abnormal distribution of the CFP+ and the Venus+ neurons within the OLP (Figure 4); we defined this phenotype as “placode defect.” In 30% (16/52) of cases we observed bundles of OE-type and VNO-type axons either overshooting past the OB or taking a misguided route (arrows in Figure 4). We also observed lack or impairment of connection with the OB, as indicated by the absence of typical glomerulus structures or their disorganized position at the OB (asterisks in Figure 4). We collectively defined these phenotypes as “connectivity and glomeruli defect.” None of these phenotypes were seen in control embryos.

*z-dlx5a* is the fish ortholog of mammalian *Dlx5*, in fact the embryonic expression territory is similar (86), and its knock-down causes craniofacial and neuronal phenotypes resembling the *Dlx5^−^*^/−^ phenotype in mice (87, 88). We depleted *z-dlx5a* in zebrafish embryos using a combination of two MO, and examined the organization of olfactory axons. Following MO injection, 72 hpf we recovered about 50% of CFP+ embryos (95% of the control injected) and about 72% of Venus+ embryos (78% of the controls). In 45% of cases (of 80 examined) we observed OPL defects, while in 54% of cases we observed OE-type and VNO-type axons targeting abnormal regions of the head near the OBs, often overshooting past the OB (arrows in Figure 4). We also observed impaired axon-OB connections, as judged by the absence of glomeruli-like bundles or their disorganized position (asterisks in Figure 4). None of these phenotypes were seen in non-injected or control MO-injected embryos. Thus, the depletion of *z-dlx5a* causes defects that recapitulates key aspects of the *Dlx5^−^*^/−^ phenotype (14, 16, 47).

**Figure 5:**
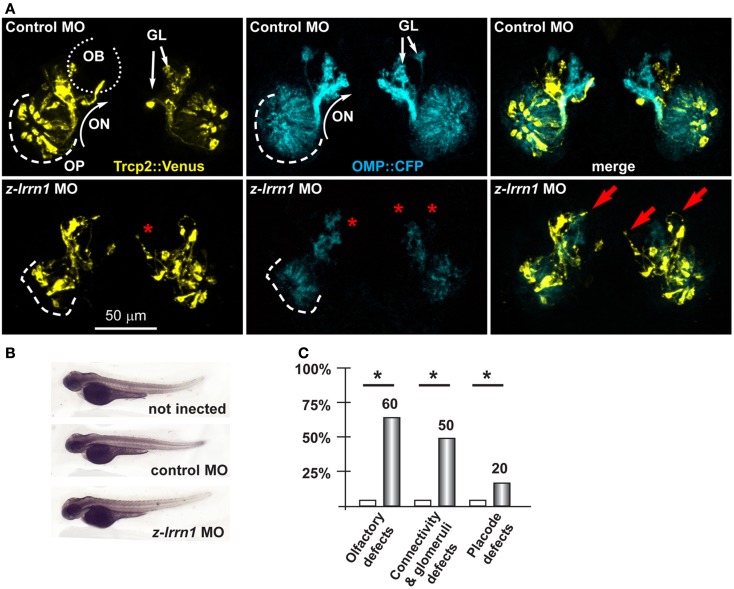
**Depletion of endogenous *z-lrrn1* in zebrafish embryos, to image the olfactory axons**. **(A)** Micrographs of *Trpc2:Venus* (YFP, yellow fluorescence) and *OMP:CFP* (cyan fluorescence) zebrafish embryos injected with control (top panels) or with anti-*z-lrrn1* (bottom panels) MOs. The control MO did not cause any significant alteration. White arrows indicate the normal axonal pathway and glomeruli in the control embryos. Red asterisks indicate absence of glomeruli. Red arrows indicate altered axonal trajectories. **(B)** Whole-mount bright field micrographs of injected embryo, showing normal embryonic morphology and growth rate. **(C)** Proportions of embryos showing either OPL disorganization, or olfactory axon mistargeting, or both (last bars) upon injection of control (open bars) or anti-*z-lrrn1* (gray bars) MOs.

Next we focused on the putative Dlx5 targets *Lrrn1, Lingo2, Islr1, St8siaVI*, and *Homer2*, whose embryonic expression in the brain and olfactory system is reported in Figure S5 in Supplementary Material. We depleted z-*lrrn1* in 1-cell zygotes by MO injection. Of the injected embryos, only approximately 50% were recovered and positive for OMP:CFP (vs. 85% in the control injected), and in a majority of these (75% of a total of 62 examined) we observed a reduction of the CFP+ signal intensity. On the contrary, we recovered a not significantly different percentage of Venus+ embryos (71 vs. 78% in the control injected) and these occasionally (20%) showed a reduced YFP fluorescent signal (Figure 5). Twenty percent of *z-lrrn1* MO-injected embryos displayed placode defects, consisting in a reduced size, altered shape, and mispositioned neuron. Half of the *z-lrrn1* MO-injected embryos displayed an altered pattern of olfactory axon fasciculation and extension, with axons overshooting or taking an ectopic route (arrows) and reduced or absent glomeruli (asterisks). Thus, the depletion of *z-lrrn1* results in a delayed differentiation of the OMP+ type olfactory neurons and altered olfactory axons trajectory and connectivity, hallmarks of the phenotypes observed in *Dlx5^−^*^/−^ mice and in *z-dlx5a* fish morphants.

**Figure 6:**
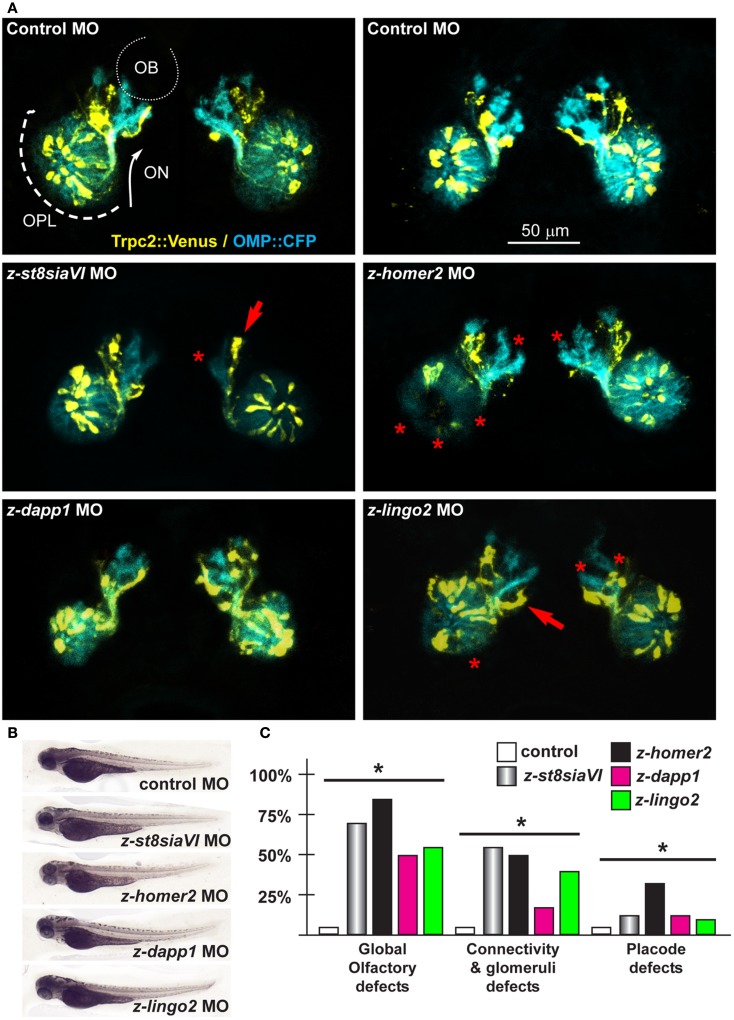
**Olfactory and VNO axons, upon depletion of endogenous *z-St8siaVI, z-lingo2*, and *z-homer2* in zebrafish embryos**. **(A)** Micrographs of *Trpc2:Venus* (yellow fluorescence) and *OMP:CFP* (cyan fluorescence) embryos injected with control MO (top panels), or injected with anti-*z-st8siaVI*, anti-*z-homer2*, anti-*z-dapp1*, and anti-*z-lingo2* MOs, as indicated on top of each image. The control MO did not cause significant alterations. Arrows indicate altered axonal trajectory, asterisks indicate absence of glomeruli or altered OPL organization. Asterisks indicate the regions of reduced fluorescence intensity. **(B)** Whole-mount bright field micrographs of injected embryo, showing normal morphology and growth rate. **(C)** Proportions of embryos showing either placode defects, connectivity and glomeruli defects, or both, upon injection of the MOs indicated above (colored bars), compared to control MO (open bars). Asterisks indicate statistical significance.

Next we depleted *z-lingo2* in reporter zebrafish embryos. Injection of the anti*-z-lingo2* MO in 1-cell embryos caused minor OP defects, consisting in altered organization and shape, while axon trajectory and glomeruli formation appeared normal (Figure 6). Next we depleted *z-st8SiaVI* in zebrafish embryos. Injection of the anti-*z-st8siaVI* MO in 1-cell embryos resulted in a phenotype affecting axon extension, trajectory, and glomeruli formation (Figure 6). Next we depleted *Homer2* in the reporter fish embryos. Injection of the anti*-z-homer2* MO in 1-cell embryos resulted in defects of OP organization and axonal targeting, plus also resulted in a reduced expression of *Trpc2*, seen as reduced YFP fluorescent signal (Figure 6). This last result might indicated that *z*-*homer2* is involved in the differentiation of the VNO-type neurons, and its depletion may delay this process. Finally, the depletion of z-*islr1* yielded no appreciable phenotype affecting the olfactory pathway (data not shown). This gene is not expressed in the embryonic OE (Figure S5 in Supplementary Material).

### Testing Dlx5, Dlx5 targets, and KS genes in zebrafish strains: The GnRH neurons

**Figure 7:**
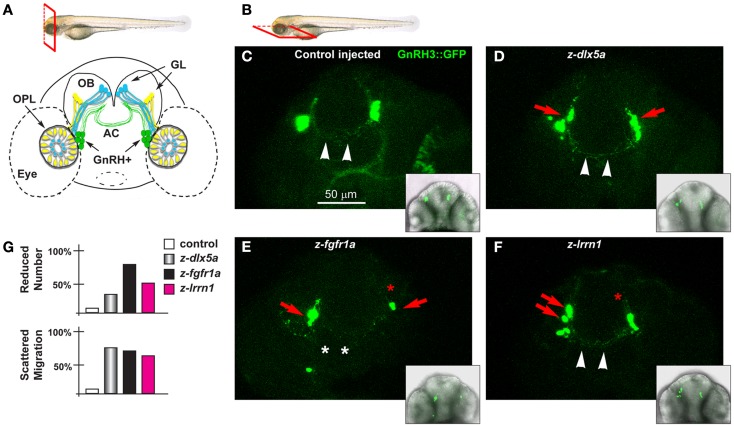
**The GnRH3 neurons upon depletion of endogenous *z-dlx5a, z-fgfr1a*, and *z-lrrn1* in zebrafish embryos**. **(A)** Scheme showing the positions of the GnRH3:GFP+ neurons (green cells), relative to the OPL, the OB, and the olfactory nerves (yellow and blue), in a frontal view. The anterior commissure is shown at the basis of the OB. On top, a scheme illustrating the view plane (frontal) used in **(A)**. **(B)** Scheme illustrating the view plane (ventral) used for the fluorescent images in **(C–F)**. **(C–F)** Micrographs of *GnRH3:GFP* zebrafish embryos, at 60 hpf, injected at the 1-cell stage with either a control MO **(C)**, with anti-*z-dlx5a* MO **(D)**, with anti-z-*fgfr1a* MO **(E)**, or with anti-*z-lrrn1* MO **(F)**. Insets on the lower right of each panel is a low-magnification merged micrograph (bright field and GFP fluorescence) of the higher magnification one. Red asterisks indicate reduced number of cells, red arrowheads indicate scattering and delayed cell migration, white arrowheads indicate the anterior commissure, white asterisks indicate absence of anterior commissure. **(G)** Quantification of the observed phenotypes, as percent over the total number of GFP+ embryos examined with each MO.

To determine whether some of the DEGs that emerged from transcription profiling of *Dlx5* mutants had some function of GnRH neuronal migration and neurite organization, we used the *GnRH3:GFP* transgenic zebrafish strain, previously reported (62–64). In these animals the GFP reporter is expressed under the transcription control of a fragment of the fish *GnRH3* promoter. The *GnRH3*-GFP+ neurons have been widely characterized, and they consist in a population of terminal nerve associated GnRH+ neurons, thought to represent the mammalian hypothalamic neurons with olfactory origin (27, 62–64, 89) (Figures 7A,B).

We depleted *z-dlx5a, z-fgfr1a/b*, and *z-lrrn1* in the *GnRH3:GFP* 1-cell zygotes, and examined the effect on the number, position, neurite organization, and commissure formation of the GFP3+ neurons associated to the terminal nerves. The depletion of *z-dlx5a* resulted in a reduced number of GFP+ neurons in 30% of cases, and in 70% of cases clearly appeared mispositioned (40 morphants examined) (Figures 7C,D; quantifications in 7G). However, the depletion of *z-dlx5a* did not affect the ability of GFP+ axons to cross the midline at the anterior commissure. Thus, a reduction of *z-dlx5a* in the fish model recapitulates (some of) the GnRH phenotype observed in the mouse model (16, 47).

The depletion of *z-fgfr1a/b* resulted in a reduced number of GFP+ neurons in 80% of cases (a total of 40 morphants examined), and in 22% of cases these neurons were clearly mispositioned, and had shorter neurites (Figure 7E). In 35% of cases the GnRH+ neurites failed to properly cross the midline in the anterior commissure. This phenotype recapitulates that seen upon depletion of *z-kal1a/b* (45, 46), thus we conclude that, based on the results of two well-established KS/nCHH genes and one KS-causing gene in the mouse, the use of MO in the *GnRH3:GFP* strain is a valid approach to examine the KS phenotype *in vivo*, and assures that future analyses on this subject will be informative.

The depletion of *z-lrrn1* in the *GnRH3:GFP* fish embryo caused a reduction in the number of GFP+ neurons in 45% of cases, and in 65% of cases caused their misposition along the terminal nerve (40 morphants examined) (Figure 7F). We also observed a reduction of their neurite length, but little of no defect of the anterior commissure. Thus also one Dlx5 target is involved in the organization and the maturation of the olfactory/GnRH system.

### Bioinformatic prediction/prioritization of new kallmann disease genes

A large set of genes has been found mutated, alone or in combination, in KS/nCHH patients, by classical mutation search approaches. However, with the exception of *KAL1* and *FGFR1*, each of them is mutated in a small fraction of the patients, and together account for no more than 40% of KS/nCHH cases. Five novel genes, functionally linked to FGF8, have been recently identified using predictive bioinformatics followed by mutation search in patients’ DNAs (7). With the exception of some genes evidently linked (*Prok2* and *ProkR2*; *FGF8* and *FGFR1*; *GnRH* and *GnRH-R*) the remaining genes appear to be unrelated, or distantly related on a functional basis. We reasoned that relationships might exist between the KS-disease genes that are not obvious, or not easily detected, or that genes may have pleiotropic functions, not know as yet. Tools have been developed that search for such relationships in databases or newly generated data, and can be used to propose candidate disease genes (90).

### Human network

**Figure 8:**
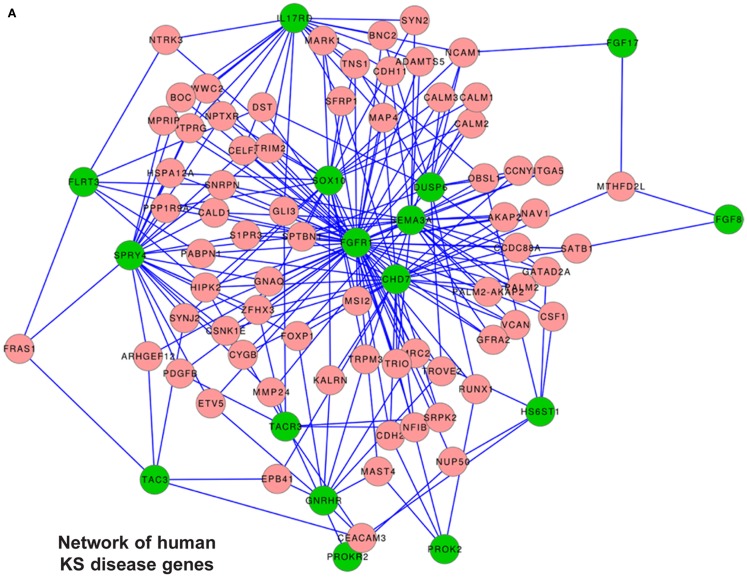

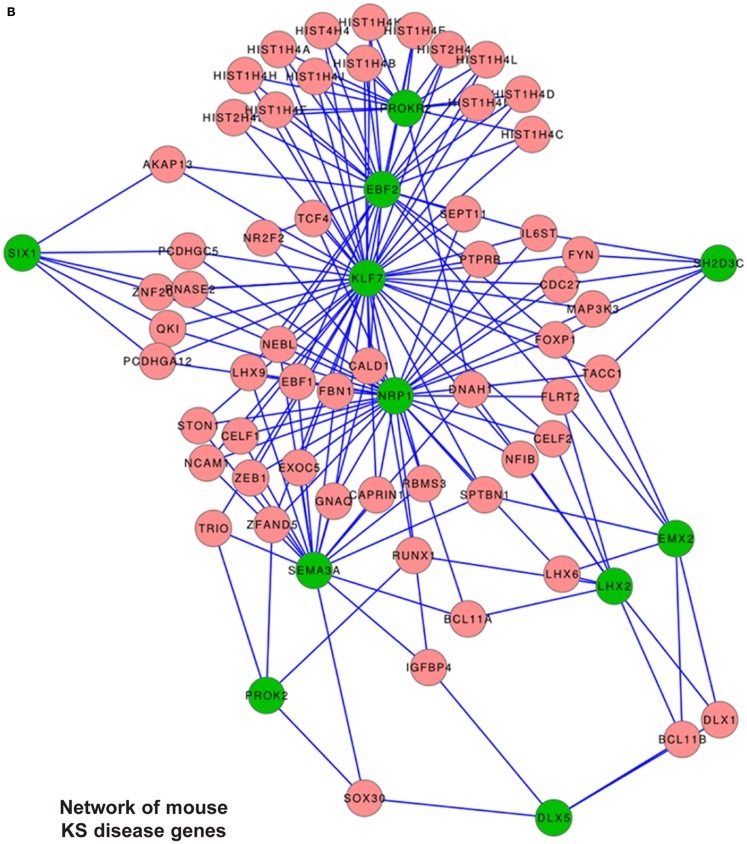
**Disease gene networks for KS**. **(A)** Position of the human KS-causing genes within the global conserved co-expression network, as computed with the TS-CoExp algorithm. **(B)** Position of the genes causing a KS-like phenotype in mice, within the global conserved co-expression network. The lists of the input (human and mouse) “disease” genes used for these analyses are in the text. For simplicity, only the genes connected with at least three input genes are shown; the genes connected with “at least one” or “at least two” input genes are available upon request. Green circles represent the input genes, pink circles represent the connected genes, lines represent statistically significant co-regulations.

We compiled a list of genes known to cause KS, or KS and nCHH, excluding those causing only nCHH; the list included *FGFR1, FGF8, KAL1, PROK-2, PROKR2, CHD7, GnRH, GnRH-R, HS6ST1, TAC3, TACR3, SOX10 e SEMA3a*. We also included *FLRT3, IL17RD, FGF17, SPRY4, DUSP6*, members of the “FGF8 synexpression” group (7) and named all these “human reference genes.” First we searched for the presence of the reference genes among the DEGs from the *Dlx5^−^*^/−^ OE vs. WT, however none of them was found. Likewise, we searched for the presence of these genes among the DEGs from the time course of the normal OE and VNO. With the exception of *GnRH*, none of the other genes was found. Next, we positioned the “human reference genes” within the global conserved co-expression network, using TS-CoExp (with the exception of *KAL1/anosmin1* that lacks a mouse ortholog and for which the conservation criterium cannot be applied) (Figure 8A).

The network representation does not consent *per se* to derive relevant information. Instead, from the data we extracted those genes connected with at least six (*N* = 2), at least five (*N* = 3), at least four (*N* = 10), at least three (*N* = 45), at least two (*N* = 317), and at least one (*N* = 1977) reference genes. We then categorized these genes by G.O. and detected an enrichment of the following G.O. categories: phosphoproteins, kinase/transmembrane receptors, cell adhesion, cell junctions, regulation of cytoskeleton, cell migration/motility, neuronal projection. Among the most connected ones we did not find any gene causing KS in mice, but we identify *TRIM2, GATAD2A, SNRPN*, and *CDH2*. Being expressed in the embryonic OE (Figure S6 in Supplementary Material), these represent most interesting genes.

Next, we used the disease gene prediction tool of TS-CoExp to identify novel candidate KS genes: the “human input genes” were taken as reference to independently prioritize the following DEG lists: (a) *Dlx5^−^*^/−^ OE vs. WT at E12; (b) EPI OE 14 vs. OPL E11 (WT); (c) EPI VNO 14 vs. OPL E11 (WT); (d) MES OE14 vs. OPL E11 (WT); (e) MES VNO 14 vs. OPL E11 (WT). From the DEG list (a) we found nine genes significantly associated with the KS phenotype, three of which (*RGS5, F2RL1*, and *DPF3*) are expressed in the embryonic OE, while two (*GATA3* and *ADAMTS5*) are expressed in the olfactory mesenchyme (Figure S6 in Supplementary Material).

From the DEG lists (b) and (c) we found 21 and 73 genes respectively, significantly associated with the KS phenotype, 19 of which are present in both lists, and the majority of these are expressed in the nasal mesenchymal. Notably, the search predicted two genes known to cause KS in the mouse, namely *Ebf2* and *Nrp1*, confirming that our analysis is in principle correct. From the DEG lists (d) and (e) we found 47 and 189 genes respectively, significantly associated with the KS phenotype, 27 of which are present in both lists. Among these, 50% show expression in the embryonic OE (*ACAN, AKAP6, ATF5, KRT18, MYT1L, NDRG1, NRXN1, SYT1*, and *TPD52*) and 50% in the olfactory-associated mesenchyme (*ANXA1, DCN, FCGRT, PAPS2, PTRF, RUNX1, S100b*, and *TGM2*). Notably, the search predicted two genes known to cause KS in the mouse, namely *Ebf2* and *Nrp1*. Furthermore, we found genes such as *AKAP6, LINGO2, LGI1*, and *LGI2* that were found among the *Dlx5* targets in the OE, and *SEMA3C* and *MET*, known to play a role in axon guidance and cell migration, respectively.

### Mouse network

We applied the approach previously used to those genes causing a KS-like phenotype in mice; the list included *Dlx5, Emx2, Klf7, Fezf1, Six1, Prok2, ProkR2, Lhx2, Shep1, Ebf2, Nrp1*, and *Sema3a*. None of them was found in the DEG lists from the *Dlx5^−^*^/−^ OE vs. wild-type, and none was found in the DEG list from the time course of the normal OE and VNO, with the exception of *Lhx2* and *GnRH*. We positioned the mouse reference genes within the conserved co-expression network (with the exception of *Fezf1* for which no result was obtained) (Figure 8B), and extracted lists of genes connected with at least five (*N* = 1), at least four (*N* = 3), at least three (*N* = 33), at least two (*N* = 261), and at least one (*N* = 1850) of them. These genes were then categorized by G.O. and we detected these over-represented terms: phosphoproteins, cell adhesion/cell junctions, neuronal projection, cell motility, cytoskeleton regulation, transcription regulation.

Next, for each of the four DEG list (from the profiling results, see above), we used the disease gene prediction tool in TS-CoExp to identify those genes most likely to be involved in KS, using the mouse reference genes as input. From the (a) list we found 13 genes, two of which (*Scn3B* and *Sv2B*) are expressed in the OE, while two (*Adamts5* and *Wnt5a*) are expressed in the olfactory mesenchyme (Figure S6 in Supplementary Material). From the (b) and (c) lists we found 19 and 65 genes respectively, 17 of which are present in both lists. Most are expressed in the nasal mesenchyme. Contrary to the human network, the mouse network did not predict any human KS gene. From the (d) and (e) lists we found 65 and 41 genes respectively, 17 of which are present in both lists and most of these are expressed in the nasal mesenchyme. Also in this case, we could not predicted any human KS genes. We found two genes: *Dcx* and *Lrrtm*, the first is relevant for migration of immature neurons, the second codes for a leucine-rich repeat protein similar to Lrrn1.

Finally, considering both the human and the mouse reference genes, five genes were found in at least three lists of genes associated with KS, namely *Dcn* (*Decorin*), *FGF7, Aspn, Ptfr*, and *Ntrk2*. Three of these, *Ntrk2, Dcn*, and *Ptfr* are clearly expressed in the juxta-OE and VNO mesenchyme, *Aspn* is ubiquitously expressed and the expression of *FGF7* is unclear. *FGF7* codes for a growth factor related to FGF22, the literature reports indications that it functions as pre-synaptic organizing molecule during hippocampal development (91, 92) and is needed for migration of enteric neurons (93). Its function in the developing OE is unknown. In conclusion, the most promising predicted KS genes are mesenchymally expressed. This is not surprising, considering that in all our profiling results, the prevalent categories are cell–cell and cell-matrix interactions, remodeling, signaling, etc…

Note: all gene lists, categorizations or tables not included in the manuscript or as supplementary material are available on request. All data are deposited at GEO repository, N° GSE52800.

## Discussion

Kallmann syndrome and nCHH are developmental/pediatric conditions phenotypically well characterized, however less well understood molecularly. Despite the number of genes found mutated in KS/nCHH patients, the majority of them still await a molecular definition. Thus, there is a strong basis to predict that many additional disease loci remain to be identified. Furthermore, the mutations found in KS patients, once thought to act alone, are now recognized as cooperating mutations, and the prevalent notion states that most KS/nCHH cases should be a bi-genic or oligo-genic disease (21, 22). This raises hopes that a more exhaustive knowledge of cooperating genes and mutations, should consent a better prognosis and possibly personalized therapies.

Methods and algorithms have been proposed to identify/prioritize novel disease genes, based on (meta)-analyses of specific profiling data, co-expression networks, genome locations, functional categorizations, protein-protein interactions, etc. (90, 94). These methods have several advantages over direct whole exome sequencing of large panels of DNAs from KS/nCHH patients (95–97). In this study, on one side we have uncovered functional classes, possible networks, and individual genes involved in the olfactory/GnRH developmental, and validated some of them in the zebrafish model recapitulating the KS phenotype. On the other side we positioned known human genes causing KS and mouse models with a KS-like phenotype in gene-co-expression networks, in order to identify genes potentially relevant for the process and candidate KS-disease genes.

Embryonic development of the olfactory connection and the migration of immature GnRH neurons are anatomically and functionally linked. Since olfactory detection is a primary sensory system in most vertebrates, and sexual maturation/reproduction is essential for the species, it is not surprising the developmental process is highly conserved and is controlled by multiple – partially redundant – networks of molecular regulations. The high degree of conservation among vertebrates justifies the use of the zebrafish embryo for *in vivo* testing (98): not only its general anatomy has not greatly changed, but also the migration of GnRH neurons along the terminal nerve, in association with the VNO axons has been overall maintained (83).

We have generated profiling data, comparing the mouse normal OE and VNO at three developmental time-points, and comparing the normal vs. a mutant model characterized by a KS-like phenotype. The data have been used to identify novel gene categories involved in the development of the olfactory system, to identify Dlx5 target genes in the OE, and to intersect this wealth of information with data from other sources. As a further step, it might be useful to generate profiles from other models of KS in the mouse (i.e., *Prok2, Fezf1*, etc.) and intersect the results searching for common patterns and co-regulations. We have attempted this, however with little success, most likely because we specifically profiled the olfactory epithelia, while data form the *Emx2* and *Klf7* models were generated from the OB. Likewise, it would be useful to intersect our profiles from the *Dlx5* model with datasets from freshly dissociated embryonic GnRH neurons.

Categories that emerge from the “time course” profiles strongly implicate extracellular matrix remodeling, cell adhesion, and cell–cell signaling molecules. This is true both for the OE and VNO development, that after all appear more “similar” than “different.” The profiles of the “pathologic” condition, i.e., the *Dlx5* knock-out model, indentified a number specific molecules in the categories of membrane receptors/adhesion molecules, axon-glia interaction molecules, but nothing specifically related to “axon elongation.” This suggests that the cell-autonomous properties of the olfactory/VNO axons to establish connections, provided (directly or indirectly) by the transcription factor Dlx5, reflects cytoskeletal properties and cell surface events, mediated by receptors, scaffold proteins and cell adhesion (see below).

Since profile data may easily lead to false positives, functional validations are mandatory; we show that the zebrafish embryo can be effectively used either to examine the trajectory and of the olfactory axons, or the status of the GnRH3 neurons. We have functionally tested five genes for olfactory axons, and three genes for GnRH3 neurons, and the results clearly indicate that the chosen genes do affect axonal trajectory and GnRH3 migration. Previous works have shown that the depletion of *z-kal1a/kal1b* in the fish embryo also causes KS-like phenotypes (45, 46), thus the use of the reporter zebrafish strains we have adopted appears to be a valid approach in which to examine new KS-causing genes in human, or genes causing a KS-like phenotype when mutated in mice. Work is continuing in this direction. The following interesting genes/categories emerge from the profile data.

### The leucine-rich repeat proteins

We identified three leucine-rich repeat transmembrane protein genes among the *Dlx5^−^*^/−^ targets, namely *Lrrn1, Lingo2*, and *Lgi1*. We functionally tested two of these using zebrafish embryos, and the results clearly show that these proteins participate in the development of the olfactory pathway. *Lrrn1* was also tested in GnRH3:GFP fish embryos, and the results show that it is required for correct GnRH neuron migration. Furthermore, *Lingo1, Lingo2*, and *Lgi* were prioritized with the human network, and *Lrrtm* (another member of this family) emerged from the mouse network.

Lrrn1 is a glycosylated single-pass transmembrane protein with 12 external leucine-rich repeats, a fibronectin domain, an immunoglobulin domain and short intracellular tails capable of mediating protein-protein interaction. Lrrn1 is closely related to drosophila tartan/capricious (trn/caps) proteins. Differential expression of trn/caps promotes an affinity difference and boundary formation between adjacent compartments in a number of contexts. The regulated embryonic expression and cellular location of these proteins suggest important roles during mouse development in the control of cell adhesion, movement, or signaling (99). Indeed, Lrrn1 has been identified as a positive and negative regulator of neurite growth (100). Lrrn1 appears to be a key regulator of the process of generating distinct cells at the midbrain-hindbrain boundary of the brain. In the chick embryo *Lrrn1* is dynamically expressed, the timing of its down-regulation correlates closely with the activation of signaling molecule expression at boundary regions. Cells over-expressing *Lrrn1* violate the boundary and this result in a loss of cell restriction at the midbrain-hindbrain boundary (101). Lrrn1 may regulate the subcellular localization of specific components of signaling or cell–cell recognition pathways in neuroepithelial cells (102).

Lingo2 is an exclusively neuronal transmembrane protein (103), containing 12 extracellular leucine-rich repeats, an immunoglobulin C2 domain and a short intracellular tail, and with a predicted structure similar to the Trk Receptor Tyrosine Kinases. In human Lingo2 been linked both to essential tremor and to Parkinson’s disease (104). Interestingly, the combination of leucine-rich repeat and immunoglobulin-like domains is found in the domain architecture of the Trk neurotrophin receptor protein. In the mouse embryo *Lingo2* is expressed in a the olfactory neurepithelium and in various areas of the adult brain (99). Lingo1, another neuron-specific member of the same family, has been shown to be a component of the Nogo66 receptor/p75 signaling complex (74). This ternary complex confers responsiveness to oligodendrocyte myelin glycoprotein, as measured by RhoA activation. Such responsiveness is linked to the inhibition of axon regeneration of neurons in the adult brain, by myelin. Thus, Lingo proteins are likely to play a role in neurite outgrowth and oligodendrocyte differentiation.

Lgi1 is a leucine-rich repeat molecule, found to be down-regulated in the absence of Dlx5. This is a secreted molecule of the SLIT family, promotes formation of stress fibers, inhibits cell movement and invasion, and enhances growth of neuronal processes on myelin-based substrates (75, 76). At the moment we have no functional data on the possible role of Lgi1 in olfactory development, yet should be explored.

### Multi-adaptor scaffold molecules

Among the *Dlx5* targets we note the presence of a set of scaffold-adaptor proteins, including *Akap6, Dapp1* (also known as *BAM32*), and *Homer2*. Akap6 belongs to a class of protein kinase A-anchoring proteins, serving as scaffolds to cluster PKA and PDE and to coordinate the timing/intracellular localization of cAMP signaling. Akap proteins also bind to- and modulate-signaling through ERK, MAPK, and PP2A (105, 106). The potential importance of this class of molecules is suggested by the fact that *Akap6* (expressed in olfactory neurons) and *Akap2* (expressed in the mesenchyme) emerge as predicted/prioritized disease genes from the human network. *Akap6* is absent in the zebrafish genome and could not be tested.

*Dapp1* codes for a signaling adapter molecule, much studied in B lymphocyte activation, in which it coordinates timing and location of signaling by PIP3 and PIP2 with that of ERK. Dapp1 also binds F-actin and Rac (107–109). *Dapp1* is not apparently expressed in the embryonic OE, nevertheless when *Dapp1* was depleted in the fish model a mild effect on axonal trajectory and OPL organization have been observed. It appears very likely that lipid signaling is involved in axonal trajectory and connectivity during olfactory development.

*Homer2* is a post-synaptic scaffold molecule, involved in receptor clustering and modulating their downstream signaling. However, recently a role for Homer2 in tuning the activity of G-proteins coupled receptors (such as the ORs) by controlling calcium influxes has been demonstrated. We carried out functional experiments depleting Homer2 in zebrafish embryos: the results provide evidence for its involvement in olfactory axonal development. Considering the established importance of the OR for olfactory axon connectivity and guidance during embryonic development, much before their role in odor perception, an embryonic role of Homer2 can be envisioned, and our results with zebrafish clearly show this.

#### The p130CAS – Shep1 regulation

Mouse embryos null for *Shep1* show retarded OE differentiation, lack of primary axonal connections with the OB and retention of GnRH neurons in the nasal mesenchyme (110). These defects are accompanied by a reduced phosphorylation of the multi-adapter scaffold molecule p130CAS in the olfactory neurons and axons. Shep1 promotes Src-dependent phosphorylation of the multi-adapter molecule p130CAS, *in vitro* (111). These data implicate the phosphorylation of p130CAS in the establishment of olfactory contacts and in GnRH neuron migration, in line with previous studies suggesting that p130CAS is required for neurite outgrowth and axon guidance (112–114). p130CAS belongs to a family of multi-adaptor and scaffold molecules that spatially and temporally collect, integrate, and modulate signals coming from RTKs and adhesion receptors (115–117), undergoing changes in phosphorylation and interacting with a large set of effectors proteins. In light of the phenotype of *Shep1^−^*^/−^ mice, the involvement of *p130CAS* in olfactory development and GnRH neuron migration is a likely possibility to be explored. Since *p130CAS* null mice are embryonic lethal (118), this study will have to be pursued via conditional deletion of *p130CAS* in the olfactory system.

*St8siaVI* codes for a sialyl-transferase, expressed by olfactory neurons. The highly related St8siaII and St8siaIV proteins are required for polysialylation the N-CAM, confer to this neuronal surface molecule anti-adhesive properties and thereby promote neurite elongation and cell migration (77–79). Thus a role for this protein in OE development is conceivable, and supported by the presented data in fish embryos.

*EphA3* codes for a receptor for the guidance molecules EphrinA3 and EphrinA5, which are expressed by VNO axons and have a preference for interacting with EphA expressing cells in the Accessory OB. Alterations of this pathway leads to abnormal topography, i.e., guidance defects, of the olfactory and VNO axons (119), indicating the EphrinA-EphA system is a positive guidance cue. *Dlx5* is co-expressed with *EphrinA3* and *EphrinA5* in the VNO, while *EphA3* is expressed in the mesenchyme near the VNO (Figure S7 in Supplementary Material). The link between Dlx5 and EphA signaling should be deeply explored.

### Genes emerging from the bioinformatic analyses

A recent work has succeeded to use bioinformatics to prioritize candidate KS genes, focusing on the FGF8 co-expression and functional network (7). Inspired by this work, we opted for an un-biased approach that simultaneously searches for links between genes apparently unrelated. Limiting our search to co-regulations, we strongly introduce the notion of conservation, reasoning that the olfactory/GnRH development is highly conserved within vertebrates. Indeed, in our work we have attempted to use also mouse KS-disease genes to run the search. The advantage is the possibility to use all the disease genes, instead of focusing only on those logically related. An additional advantage of the present work derives from combining bioinformatic predictions, putative gene functions, phenotype descriptions, and information from the literature with “wet” profiling data *specifically* obtained from embryonic olfactory tissues.

The “human” network was able to predict few mouse KS gene (*Ebf2* and *Nrp1*), providing an evidence that the algorithm is effective. The outcome, both in terms of individual genes and the G.O. classes, assures that the pipeline works. The addition of protein-protein interaction data (when made available) or other data to carry out meta-analyses will certainly refine the results. On the contrary, it appears that the “mouse” gene network is little informative, i.e., less able to predict the human KS genes. This might be due to the fact that the definition of mouse input gene is based on accurate phenotypic analyses on the olfactory system, reported in the literature, that scientist don’t routinely conduct (we may miss many other genes) or it is incomplete and does not examine olfactory axons but only hypothalamic GnRH neurons.

The predicted/prioritized genes emerging from our analyses may represent a novel set of KS-causing genes, or genes that might contribute when co-mutated with others. While the use of modern sequencing approach (WES) on KS patients’ DNAs is the straight-forward approach to define their role in the human disease, additional filters may be needed to further prioritize these genes, i.e., testing their function on GnRH3+ neurons fish embryos.

*TRIM2 – tripartite motif containing 2*, codes for an E3 ubiquitin-protein ligase that has been implicated in ubiquitination of neurofilament light chains. TRIM2 controls the dynamic of neuronal cytoskeleton, by which determines the specification of the choice of the axonal vs. dendritic projection in hippocampal neurons (120).

*CDH2 – cadherin-2*, also known as *N-cadherin*, codes for a well known calcium-dependent neuronal cell adhesion molecule that contributes to the formation of neural circuits by regulating growth cone migration and synapse formation. In the mammalian embryonic neocortex, radial migration is instructed by several signals that include homophilic interactions mediated by Cdh2 (121), and the fish embryo Cdh2 is involved in neuroblast migration within the hindbrain (122, 123). Chd2 function is required for guidance of afferent fibers of cranial sensory neurons (124) and regulates motor axon growth and branching, in fish embryos (125). During olfactory development, Cdh2 is expressed by receptor neurons and closely parallels expression of γ-catenin in neuronal axons (126), thus Cdh2 is positioned to underlie the formation of olfactory primary olfactory connections.

*ADAMTS5* codes for a disintegrin-like and metallopeptidase extracellular protease, with thrombospondin-like motif. Adamts5 plays a role in the specification and patterning of progenitor cells in the lateral and medial ganglionic eminences (127). The proteolytic cleavage of astrocyte-derived proteoglycan, exerted by Adamts5, loosens the matrix environment and promotes neurite outgrowth (128). Being predicted by both the human and the mouse disease-gene networks, *Adamts5* appears to be a very interesting candidate.

*RGS5 – regulator of G-protein signaling 5*, codes for a protein that accelerates the inactivation of Gα-dependent signaling in various cells types. Down-regulation of *RGS5* induces GPCR-mediated signaling pathways and promotes migration of vascular and cancer cells (129, 130). A role of this protein in promoting the migration of GnRH neurons is possible, although RGS5 null mice don’t show any obvious phenotype (131).

*DPF3 – D4, zinc* and *double PHD fingers, family 3*, codes for a component of the BAF chromatin remodeling protein, and acts a transcription co-activator in SWI/SNF complex-activation (132). DPF3 functions to activate transcription of the target genes *Pitx2* and *Jmjd1c* in association with the BAF complex, and binds histone H3 and H4 in an acetylation-dependent manner (133, 134). How this could be relevant for olfactory development, GnRH neuron migration and KS, is unclear.

*FGF7* has been proposed to act as a pre-synaptic organizing molecule in the mammalian brain, and in particular during hippocampal development. Indeed FGF7-deficiency impairs inhibitory synapse formation, which results in mossy fiber sprouting and enhanced neurogenesis (91, 92). Neutralization of FGF7 inhibits pre-synaptic differentiation of mossy fibers at contact with granule cells, and inactivation of FGFR2 has similar effects (92). In neurons, FGFs and cell adhesion molecules stimulate neurite outgrowth via activation of FGF receptors. A role for FGF7 for the migration of enteric neuroblasts has been suggested from analyses of CAMs and FGFs expression in Hirschsprung Disease patients (93).

## Conclusion

The molecular control over the ability of olfactory axons to contact the anterior forebrain, and/or the ability of GnRH neurons to efficiently migrate and home to the hypothalamus, entails numerous proteins of various functional classes, many of which appear to be directly and indirectly involved in matrix remodeling and signaling. Indeed, the data indicate that the navigation of OE and VNO axons is mostly governed by cell–cell and cell-matrix cues, rather than intrinsic properties of the axons. These include a set of scaffold molecules that, for their nature, are strong candidates for playing a key role in guiding axonal elongation-guidance and connectivity, as well as for GnRH neuron migration and homing. These molecules will be of great interest for developmental biologists.

Perturbations in the expression and sequence (mutations) of these molecules and in their associated gene networks may cause phenotypes similar to KS, a possibility that can be rapidly tested in zebrafish strains, and eventually in the mouse. Human geneticists should consider these molecules for mutation screens. This opens the possibility to test them in the mammalian model and to search for mutations in large collections of DNAs from KS/nCHH patients, hereditary, or sporadic, with the hope to find mutations, alone or in combination with mutations in known KS/nCHH genes.

Finally we show the validity of approaches based on high-throughput data generation and predictive bioinformatics to identify genes potentially relevant for specific developmental processes, and ultimately for disease. Indeed, we have uncover a set of molecules that might be candidate disease genes, to be tested in future mutation screens.

## Conflict of Interest Statement

The authors declare that the research was conducted in the absence of any commercial or financial relationships that could be construed as a potential conflict of interest.

## Supplementary Material

The Supplementary Material for this article can be found online at http://www.frontiersin.org/Journal/10.3389/fendo.2013.00203/abstract

## Footnotes

^1^www.affymetrix.com

^2^http://david.abcc.ncifcrf.gov/

^3^http://www.genome.jp/kegg/pathway.html

^4^www.genepaint.org

^5^www.eurexpress.org

^6^http://www.mbcunito.it/cbu/ts-coexp

^7^http://www.ensembl.org/index.html

^8^http://genome.ucsc.edu

^9^http://www.ncbi.nlm.nih.gov/RefSeq

^10^http://www.informatics.jax.org/

^11^http://www.omim.org/
