# Supplementary material for: Profiling, Bioinformatic, and Functional Data on the Developing Olfactory/GnRH System Reveal Cellular and Molecular Pathways Essential for This Process and Potentially Relevant for the Kallmann Syndrome
Source: Front Endocrinol (Lausanne). 2013 Dec 31;4:203. doi: 10.3389/fendo.2013.00203 (PMC3876029; doi:10.3389/fendo.2013.00203)

Supplementary Figure 1. Collection of olfactory tissue samples from E14.5 embryos. Embryonic heads were visually recognized for being *Dlx5*-/- or wild-type, included in low-melting point agarose and sectioned (200 μm thick) with a vibratome. Sections were collected in RNAse-free PBS and manually microdissected with fine pins to remove the Olfactory Epithelium (OE), the Vomero-Nasal Organ (VNO) (drawn in pink), or the Mesenchyme adjacent to either the OE or the VNO.

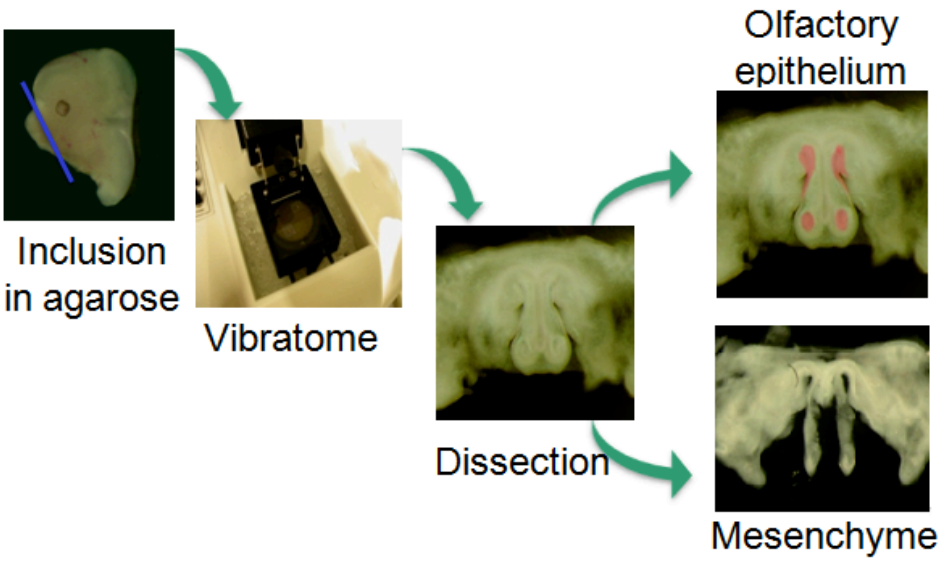

Supplementary Figure 2.

A. Real-Time qPCR to quantify the abundance of mRNAs of purely EPI genes (*FoxJ1*, *Fmo2* and *Ehf*), measured in OE (mixed samples) or in purely mesenchymal (MES) samples. In the MES samples, we estimated an average of less than 8% of EPI tissue.

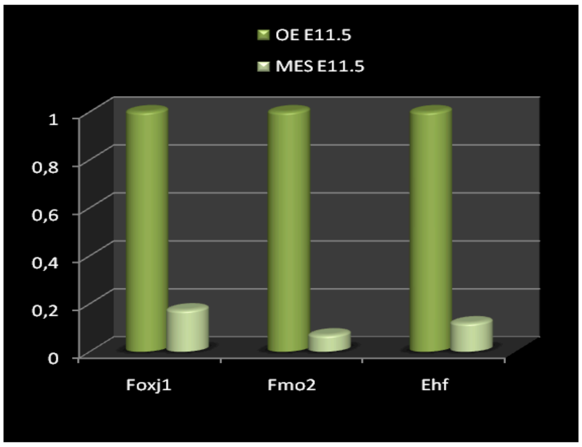

B. Real-Time qPCR to quantify the abundance of mRNAs of purely MES genes (*Sp7* and *Lect1*), measured in OE (mixed samples) and in purely mesenchymal (MES) samples. In the OE samples we estimated an average of 15% of MES tissue.

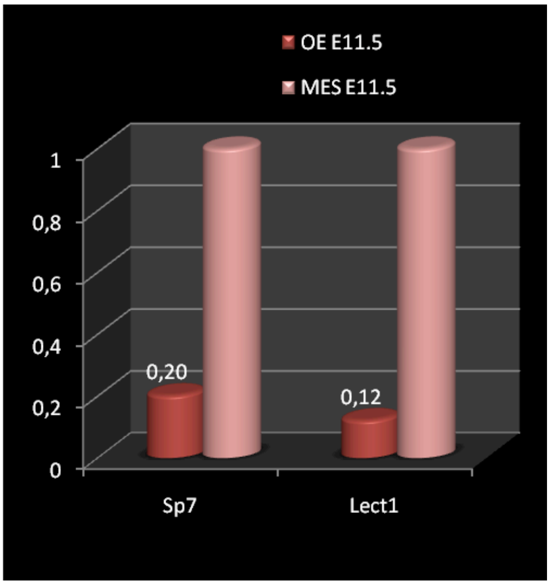

Supplementary Figure 3. The Dlx5 Position Weight Matrix of Dlx5 extracted from the Jaspar data base. This matrix was used to predict Dlx5 sites in the human and mouse genome, using conservation as a filtering criterium. More information in the Materials and Methods section and in Vieux-Rochas et al. PLoS One 2013.

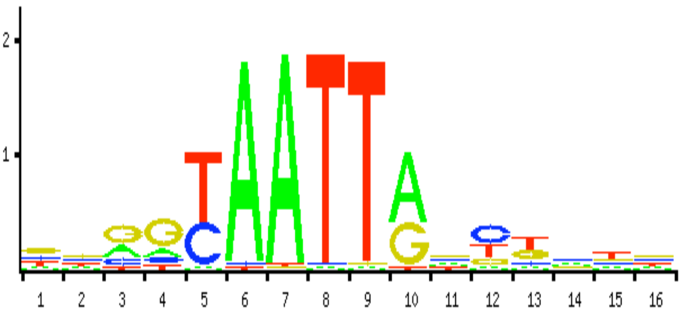

Supplementary Figure 4. A. Scheme showing the organization of the olfactory-type and VNO-type axons in the zebrafish embryos, relative to the olfactory placodes (OPL), the olfactory bulbs (OB), in frontal view. The olfactory nerve (ON) and the glomeruli (GL) are also indicated.
B. Fluorescent micrographs to image the OE-type and the VNO-type axons by the use of, respectively, the *OMP::CFP* and the *Trpc2::Venus* transgenic fish strains.

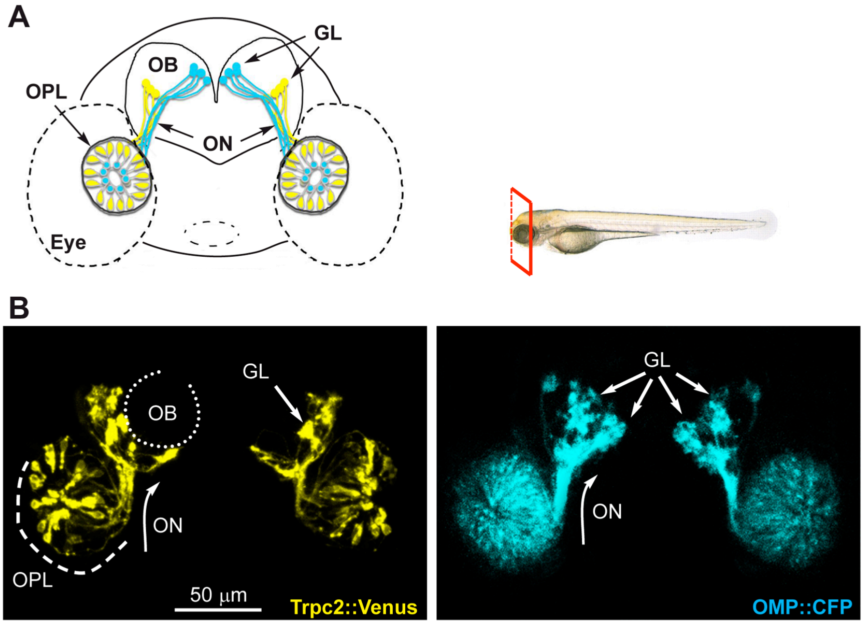

Supplementary Figure 5. Expression of *Dlx5* and selected *Dlx5* targets (indicated on the top left of each micrograph) in E14.5 mouse embryos, by in situ hybridization. Images were obtained from www.genepaint.org. Red arrows indicate expression in the olfactory neuroepithelium. Red asterisks indicate lack of expression.

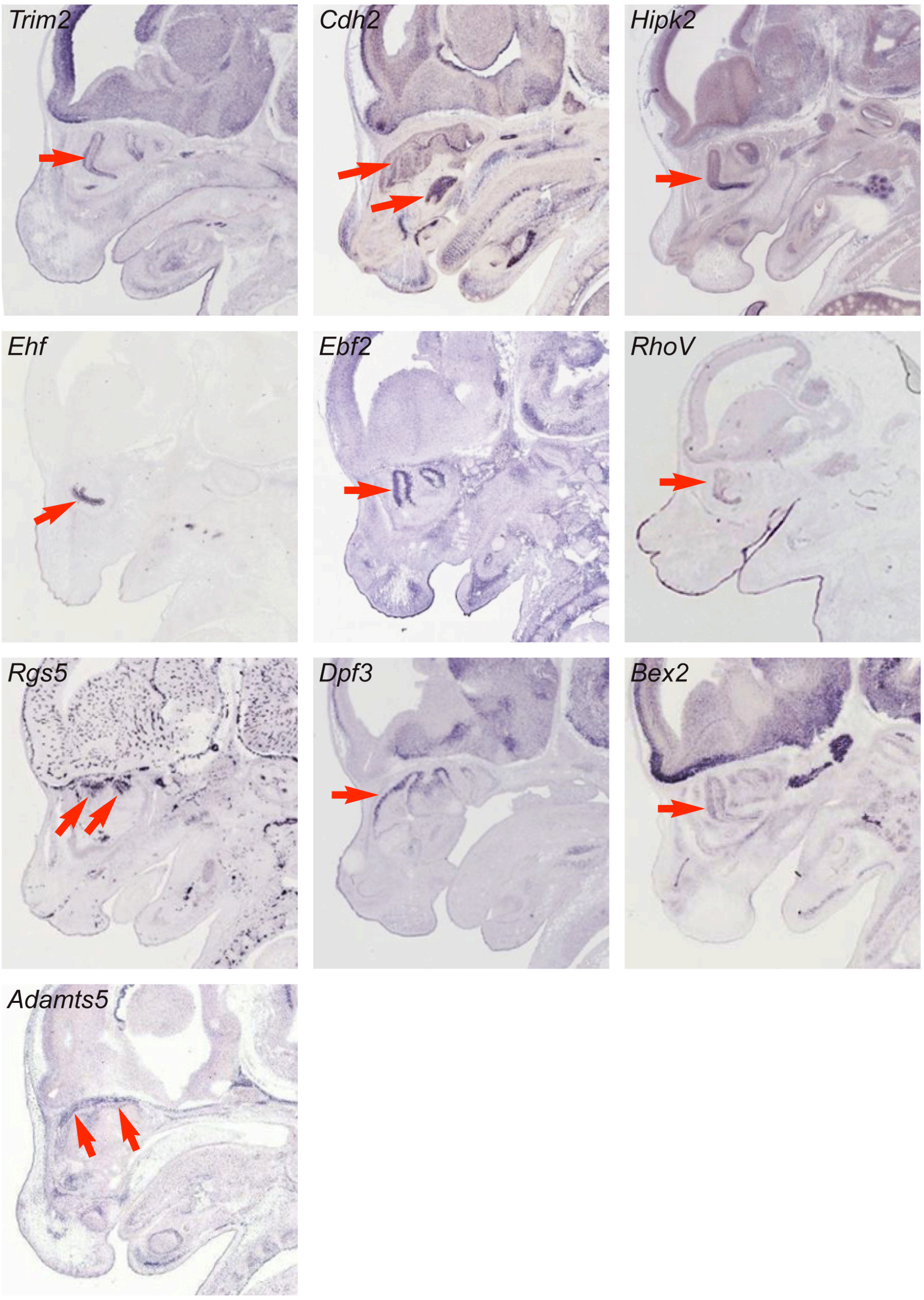

Supplementary Figure 6. Expression of genes (indicated on the top left of each micrograph) predicted/prioritized from the known human and mouse KS-disease genes, in E14.5 mouse embryos, by in situ hybridization. Images were obtained from www.genepaint.org. Red arrows indicate expression in the olfactory neuroepithelium, with the exception of *Adamts5*, whose expression is mesenchymal.

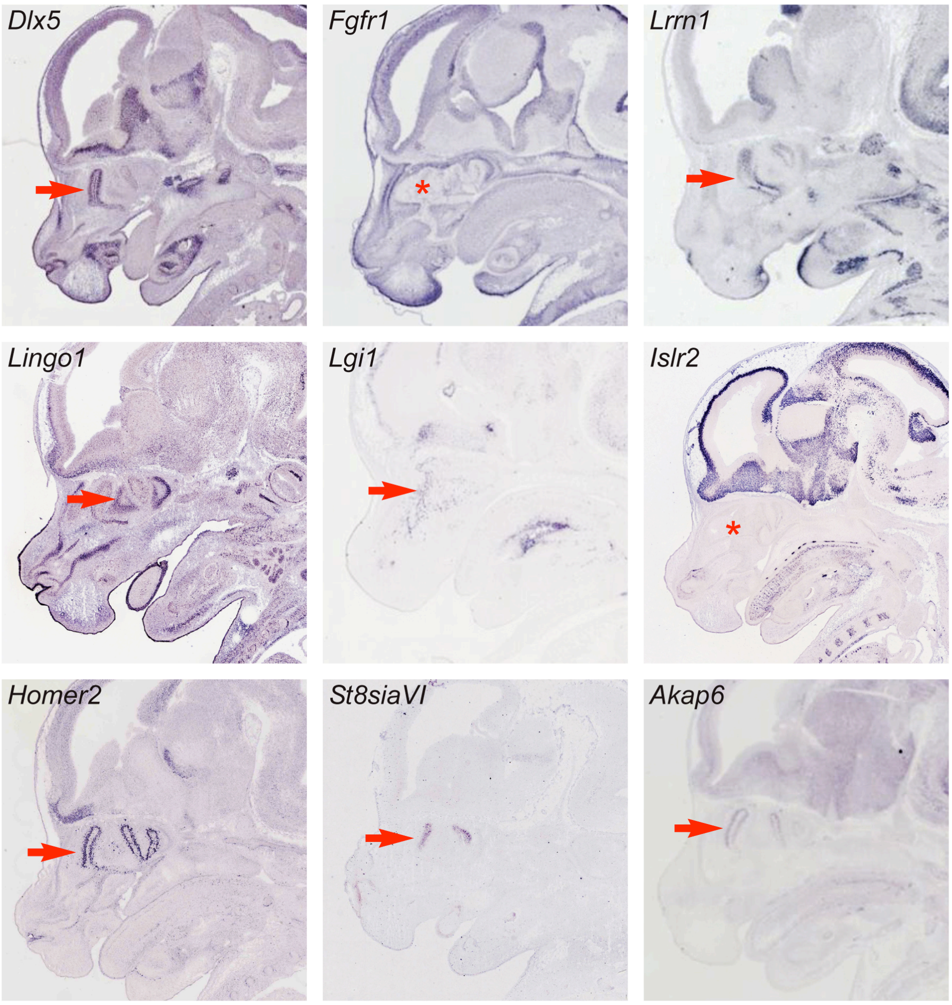

Supplementary Figure 7. Detailed expression of *EphA3* receptor, *EphrinA3, EphrinA5* and *Dlx5* in the olfactory region (OE, OB and VNO) of the mouse embryo. Except for *Dlx5*, in situ hybridization images were obtained from the publicly available database www.genepaint.org

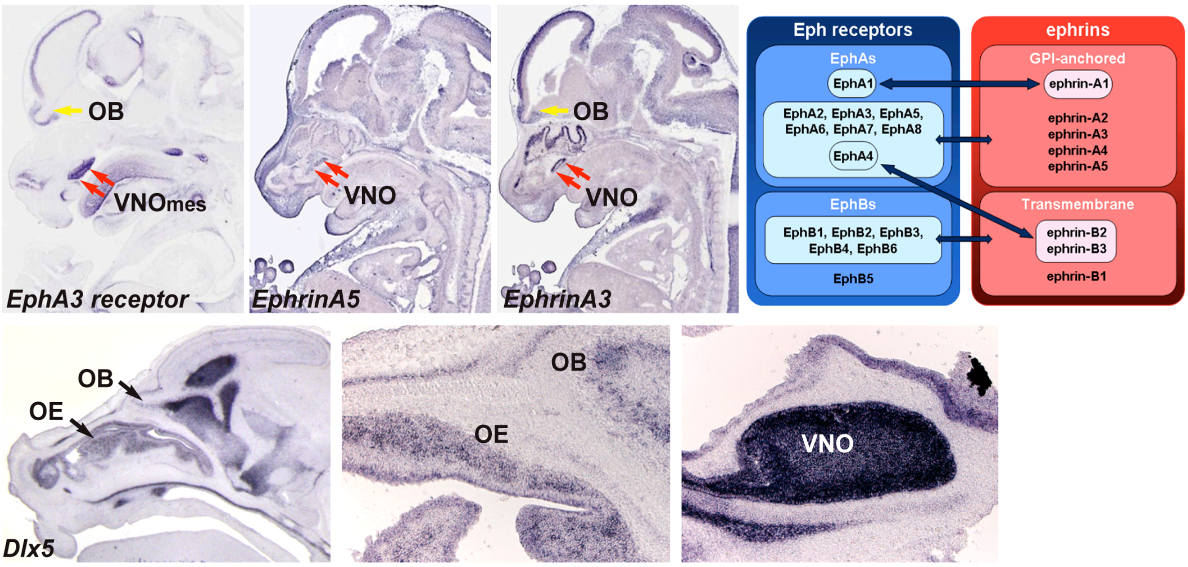

Supplement: Supplementary file 1 [file 76323_Merlo_Presentation1.PDF]
