# Supplementary material for: Profiling, Bioinformatic, and Functional Data on the Developing Olfactory/GnRH System Reveal Cellular and Molecular Pathways Essential for This Process and Potentially Relevant for the Kallmann Syndrome
Source: Front Endocrinol (Lausanne). 2013 Dec 31;4:203. doi: 10.3389/fendo.2013.00203 (PMC3876029; doi:10.3389/fendo.2013.00203)
Supplement: Supplementary file 2 [file 76323_Merlo_Presentation2.PDF]

Supplementary Table I Sequence of primers used for Real-Time qPCR (mouse)

Akap6	F	CGGTCGCGTCATTTTCTC
	R	GGGTTATCTTCACACACCATGA
Cnga2	F	AGGGAAAGGGCACCAAAA
	R	GCAATGACAAACAACCAAGC
Dlx5	F	CTGGTGACTGTGGCGAGTTA
	R	CAGAAGAGTCCCAAGCATCC
Ermn	F	GAAGACATGGCTTTCCGAGA
	R	CGCATCTCCTCTCTGCTCA
Homer2	F	GGAACAGCTATCGGATCATCA
	R	TCGGGGTGATAGTGCTGTTT
Islr2	F	AGGCAGAGCTGGAGGAGAC
	R	GATCTGCAGGGATCTGACCT
Lgi1	F	CAAAGGCCTGGATTCCTTAAC
	R	CCACAGTTGCATTGGTATGG
Lingo2	F	GGTACAGCAAAAGGAGTGCAT
	R	GAGTAACACCACAGCCAGACC
Lrrn1	F	GTCAGCCCAGCCTTAGGAC
	R	CTTGAACCGTATCACTCAATGC
Rtp1	F	CCCAACCTCAAGCACAATG
	R	AGCACCAGGAACAGTGGAAC
Snap25	F	CGGAATTCGTGGATGAACGGGAGCAGA
	R	CGGAATTCGAATTTTCCTAGGTCCGTCAAA
St8siaVI	F	TCCCCATTCGAGAGAACATT
	R	CACACTGGTTATAGGGATAGTCCA
Gabrb2	F	CGATGGCACTGTCCTGTATG
	R	ATACCGCCTTAGGTCCATCA
Abcd2	F	GCGCGGATGTTTTACCATAA
	R	GATGCTCACGGCACTGGTA
Dapp1	F	TCCAATGGACGTGATGGTAG
	R	GTTTGACAGAGTCTTTGGCTCTC
Ehf	F	TGTGATAGCTTCCTGCCT TCT
	R	CACCACTTCCTTCCAGAATCA
FoxJ1	F	GAACTTCTTCCAGAACCTTCCTC
	R	CCTCCGAACACGAATGTGA
Fmo2	F	TGATGGCAGACATTGTTGAAA
	R	CAACGTAGTTGGTCTGCAGTATCT
Lect1	F	TCTTCGTAGTTAGCTGGCATGA
	R	CAGTGACCAAGCAGAGCATC
Sp7	F	AGAGATCTGAGCTGGGTAGAGG
	R	AAGAGAGCCTGGCAAGAGG
GAPDH	F	TGTCAGCAATGCATCCTGCA
	R	TGTATGCAGGGATGATGTTC

Supplementary Table II. Sequences of Morpholino Oligonucleotides used for depletion of specific proteins in zebrafish embryos.

Gene	Description	Zfish homolog	Sequence	Morpholino targeting	Phenotype
**Dlx5**	distall-less homeobox 5	***dlx5a***	5'-CGAATACTCCAGTCATAGTTTGGAT-3'	ATG blocking	olfactory sensory neuron axon mistargeting, glomerular map formation delay, olfactory placode anatomical malformation, olfactory sensory neuron differentiation delay
			5'-TTATATAACAACTAACCTTGCGGGC-3'	EX1-in1 splice junct.	
**Lrrn1**	leucine rich repeat protein 1, neuronal	***lrrn1***	5'-AAGAAAGTCCCTCTAGCCATTCTGA-3'	ATG blocking	olfactory sensory neuron axon mistargeting, glomerular map formatio delay, olfactory placode anatomical malformation
**Islr2**	immunoglobulin superfamily containing leucine-rich repeat 2	***islr2***	5'-AGGTATTTGGTTGCCATTTTCAGCT-3'	ATG blocking	no phenotype
**Homer2**	homer homolog 2	***homer2***	5'-CATGTATCTGTCTGACCTTTGTTCC-3'	EX1-in1 splice junct.	olfactory sensory neuron differentiation delay
**DAPP1**	dual adaptor for phosphotyrosine and 3-phosphoinositides 1	***dapp1***	5'-TGCTCTGAAAAAAGCGTGGACATGA-3'	in2-EX3 splice junct.	no phenotype
**Lingo2**	leucine rich repeat and Ig domain containing 2	***lingo2***	5'-CTCAAACAGTCCACCATACTGTCAT -3'	ATG blocking	olfactory sensory neuron axon mistargeting, glomerular map formatio delay, olfactory placode anatomical malformation
**St8siaIV**	ST8 alpha-N-acetyl-neuraminide alpha-2,8-sialyltransferase 6	***st8sia6***	5'-AAGAGTCCTCATAACGCGCATCCAC-3'	ATG blocking	olfactory sensory neuron axon mistargeting, glomerular map formatio delay, olfactory placode anatomical malformation
**Fgfr1**	Fibroblast growth factor receptor 1	***fgfr1a***	5’-GCAGCAGCGTGGTCTTCATTATCAT-3’<br>5’-CAAGAGAGCGCATGCTGCTTACGTA-3’	ATG blocking<br>Splice junction	olfactory sensory neuron axon mistargeting, glomerular map formatio delay, olfactory placode anatomical malformation

Supplementary Table III. Differentially Expressed Genes in OE E14.5 vs. OPL 11.5. Raw EPI data subtracted of the MES data. The gene symbols, gene names, Entrez gene ID, fold-change and significance are reported. DEGs are ranked according to the "fold-change". For simplicity, the non-annotated probes and the *OR* genes have been eliminated from the Table.

entrez ID	symbol	gene name	M	FDR
18843	Plunc	palate, lung, and nasal epithelium associated	6,8106	0
66695	Aspn	asporin	6,5216	0
15891	Ibsp	integrin binding sialoprotein	6,2385	0
13108	Cyp2g1	cytochrome P450, family 2, subfamily g, polypeptide 1	6,0329	0
16840	Lect1	leukocyte cell derived chemotaxin 1	5,9658	0
19285	Ptrf	polymerase I and transcript release factor	5,7112	0
12409	Cbr2	carbonyl reductase 2	5,5542	0
244180	E030002O03Rik	RIKEN cDNA E030002O03 gene	5,3691	0
13179	Dcn	decorin	5,3554	0
66957	Serpinb11	serine (or cysteine) peptidase inhibitor, clade B (ovalbumin), member 11	5,3173	0
19695	Reg3g	regenerating islet-derived 3 gamma	5,2387	0
11475	Acta2	actin, alpha 2, smooth muscle, aorta	5,1567	0
13107	Cyp2f2	cytochrome P450, family 2, subfamily f, polypeptide 2	5,1613	0
20753	Sprr1a	small proline-rich protein 1A	4,9551	0
17180	Matn1	matrilin 1, cartilage matrix protein	5,0287	0
12842	Col1a1	collagen, type I, alpha 1	4,8225	0
12839	Col9a1	collagen, type IX, alpha 1	4,6579	0
21826	Thbs2	thrombospondin 2	4,513	0,0011
11522	Adh1	alcohol dehydrogenase 1 (class I)	4,3679	0,001
13717	Eln	elastin	4,1558	0,001
213436	Zcchc5	zinc finger, CCHC domain containing 5	4,2173	9,00E-04
11595	Acan	aggrecan	4,1607	9,00E-04
13849	Ephx1	epoxide hydrolase 1, microsomal	4,0575	0,0012
16952	Anxa1	annexin A1	4,0471	0,0012
12818	Col14a1	collagen, type XIV, alpha 1	4,0688	0,0012
14261	Fmo1	flavin containing monooxygenase 1	3,9655	0,0011
18212	Ntrk2	neurotrophic tyrosine kinase, receptor, type 2	3,9969	0,0011
21817	Tgm2	transglutaminase 2, C polypeptide	4,0017	0,001
13661	Ehf	ets homologous factor	4,0068	0,001
13516	Epyc	epiphycan	3,9413	0,001
17183	Matn4	matrilin 4	3,9273	9,00E-04

18295	Ogn	osteoglycin	3,9689	8,00E-04
16667	Krt17	keratin 17	3,9571	8,00E-04
22117	Tst	thiosulfate sulfurtransferase, mitochondrial	3,8494	8,00E-04
16678	Krt1	keratin 1	4,1192	0,0013
74145	F13a1	coagulation factor XIII, A1 subunit	3,7981	0,0012
55990	Fmo2	flavin containing monooxygenase 2	3,8158	0,0012
20256	Clec11a	C-type lectin domain family 11, member a	3,8322	0,0012
66166	S100a14	S100 calcium binding protein A14	3,7382	0,002
258609	Olfr305	olfactory receptor 305	3,8145	0,0022
16948	Lox	lysyl oxidase	3,6659	0,0023
11922	Neurod6	neurogenic differentiation 6	3,7896	0,0023
18994	Pou3f4	POU domain, class 3, transcription factor 4	3,6952	0,0022
13732	Emp3	epithelial membrane protein 3	3,676	0,0026
116872	Serpinb7	serine (or cysteine) peptidase inhibitor, clade B, member 7	3,7282	0,0025
12833	Col6a1	collagen, type VI, alpha 1	3,5989	0,0027
20259	Scin	scinderin	3,687	0,0028
105785	Kdelr3	KDEL (Lys-Asp-Glu-Leu) endoplasmic reticulum protein retention receptor 3	3,5068	0,0028
18032	Nfix	nuclear factor I/X	3,5088	0,0027
16669	Krt19	keratin 19	3,521	0,0027
114332	Lyve1	lymphatic vessel endothelial hyaluronan receptor 1	3,4986	0,0028
18121	Nog	noggin	3,5417	0,0028
20379	Sfrp4	secreted frizzled-related protein 4	3,5261	0,0029
17112	Tm4sf1	transmembrane 4 superfamily member 1	3,4764	0,0028
17182	Matn3	matrilin 3	3,5359	0,0029
19132	Prph	peripherin	3,5105	0,0029
11828	Aqp3	aquaporin 3	3,433	0,0029
213043	Aox3l1	aldehyde oxidase 3-like 1	3,4515	0,0028

11676	Aldoc	aldolase C, fructose-bisphosphate	3,3714	0,003 4
258206	Olfr1264	olfactory receptor 1264	3,3876	0,003 3
12950	Hapln1	hyaluronan and proteoglycan link protein 1	3,5629	0,003 4
11858	Rnd2	Rho family GTPase 2	3,3233	0,004 3
81799	C1qtnf3	C1q and tumor necrosis factor related protein 3	3,3245	0,004 3
21923	Tnc	tenascin C	3,4312	0,004 6
21924	Tnnc1	troponin C, cardiac/slow skeletal	3,2681	0,004 9
117167	Steap4	STEAP family member 4	3,2714	0,004 8
21824	Thbd	thrombomodulin	3,2804	0,004 7
12816	Col12a1	collagen, type XII, alpha 1	3,308	0,005 3
56429	Dpt	dermatopontin	3,3497	0,005 3
170798	AY036118	cDNA sequence AY036118	3,5887	0,005 4
17968	Ncam2	neural cell adhesion molecule 2	3,452	0,005 5
53315	Sult1d1	sulfotransferase family 1D, member 1	3,2404	0,005 6
98660	Atp1a2	ATPase, Na+/K+ transporting, alpha 2 polypeptide	3,2008	0,006 1
13809	Enpep	glutamyl aminopeptidase	3,1669	0,006 8
12577	Cdkn1c	cyclin-dependent kinase inhibitor 1C (P57)	3,1487	0,006 8
76365	Tbx18	T-box18	3,2393	0,007 8
14238	Foxf2	forkhead box F2	3,2965	0,008 2
14132	Fcgrt	Fc receptor, IgG, alpha chain transporter	3,1056	0,008 5
12824	Col2a1	collagen, type II, alpha 1	3,1369	0,009 2
330216	Mblac1	metallo-beta-lactamase domain containing 1	3,1697	0,009 1
18013	Neurod2	neurogenic differentiation 2	3,0808	0,009
13522	Adam28	a disintegrin and metallopeptidase domain 28	3,0551	0,009 2
22268	Upk1b	uroplakin 1B	3,0951	0,009 5
12643	Chad	chondroadherin	3,0823	0,010 1
11752	Anxa8	annexin A8	3,144	0,010 2

19737	Rgs5	regulator of G-protein signaling 5	3,0171	0,0102
16803	Lbp	lipopolysaccharide binding protein	3,0588	0,0102
18378	Omp	olfactory marker protein	3,0711	0,0105
170574	Sp7	Sp7 transcription factor 7	3,0507	0,0104
246048	Chodl	chondrolectin	3,1474	0,0112
110012	Gm16517	predicted gene, Gm16517	2,9729	0,0114
107250	Kazald1	Kazal-type serine peptidase inhibitor domain 1	2,9538	0,012
110935	Atp6v1b1	ATPase, H+ transporting, lysosomal V1 subunit B1	2,9426	0,012
18979	Pon1	paraoxonase 1	2,9912	0,0124
54156	Egfl6	EGF-like-domain, multiple 6	2,9814	0,0123
67295	Rab3c	RAB3C, member RAS oncogene family	3,0176	0,0122
19737	Rgs5	regulator of G-protein signaling 5	2,9203	0,0124
21375	Tbr1	T-box brain gene 1	2,9949	0,0124
208890	Slc26a7	solute carrier family 26, member 7	2,9244	0,0126
13390	Dlx1	distal-less homeobox 1	3,0231	0,0125
15375	Foxa1	forkhead box A1	2,8913	0,0129
20888	Sult1c1	sulfotransferase family, cytosolic, 1C, member 1	2,9774	0,0131
20203	S100b	S100 protein, beta polypeptide, neural	2,9801	0,0138
16905	Lmna	lamin A	2,8903	0,0137
16665	Krt15	keratin 15	2,9492	0,0136
21743	Inmt	indolethylamine N-methyltransferase	2,8677	0,0142
77125	Il33	interleukin 33	2,863	0,0142
56213	Htra1	HtrA serine peptidase 1	2,8479	0,0141
56188	Fxyd1	FXYD domain-containing ion transport regulator 1	2,8289	0,0149
16661	Krt10	keratin 10	2,9642	0,015
20193	S100a1	S100 calcium binding protein A1	2,8266	0,0151
14859	Gsta3	glutathione S-transferase, alpha 3	2,8324	0,0151
15229	Foxd1	forkhead box D1	2,8256	0,015

210622	Pamr1	peptidase domain containing associated with muscle regeneration 1	2,8474	0,0149
259300	Ehd2	EH-domain containing 2	2,8161	0,0157
97440	B3gnt9-ps	UDP-GlcNAc:betaGal beta-1,3-N-acetylglucosaminyltransferase 9, pseudogene	2,8099	0,0162
74186	Ccdc3	coiled-coil domain containing 3	2,8122	0,0163
14406	Gabrg2	gamma-aminobutyric acid (GABA) A receptor, subunit gamma 2	2,9812	0,018
11826	Aqp1	aquaporin 1	2,7757	0,0183
259057	Olfr649	olfactory receptor 649	2,9959	0,0192
207596	Thsd4	thrombospondin, type I, domain containing 4	2,7367	0,0196
14623	Gjb6	gap junction protein, beta 6	2,742	0,0206
17533	Mrc1	mannose receptor, C type 1	2,7065	0,0207
63873	Trpv4	transient receptor potential cation channel, subfamily V, member 4	2,7292	0,0215
223272	Itgbl1	integrin, beta-like 1	2,698	0,0215
14560	Gdf10	growth differentiation factor 10	2,75	0,0217
21384	Tbx15	T-box 15	2,7377	0,0217
14178	Fgf7	fibroblast growth factor 7	2,7574	0,0218
67951	Tubb6	tubulin, beta 6	2,6896	0,0219
75600	Calml4	calmodulin-like 4	2,7024	0,022
20724	Serpinb5	serine (or cysteine) peptidase inhibitor, clade B, member 5	2,8605	0,0222
17181	Matn2	matrilin 2	2,6677	0,0223
73121	Fam101a	family with sequence similarity 101, member A	2,6827	0,0224
232400	BC048546	cDNA sequence BC048546	2,6505	0,0231
13507	Dsc3	desmocollin 3	2,845	0,0232
12837	Col8a1	collagen, type VIII, alpha 1	2,6671	0,0239
14184	Fgfr3	fibroblast growth factor receptor 3	2,6721	0,0244
50916	Irx4	Iroquois related homeobox 4 (Drosophila)	2,6654	0,0242
18858	Pmp22	peripheral myelin protein 22	2,6395	0,0246
67405	Nts	neurotensin	2,6388	0,0247

258387	Olfr720	olfactory receptor 720	2,7039	0,024<br>6
17313	Mgp	matrix Gla protein	2,6652	0,024<br>8
224055	Rtp2	receptor transporter protein 2	2,6201	0,025<br>3
243339	Tmem130	transmembrane protein 130	2,7805	0,025<br>3
12789	Cnga2	cyclic nucleotide gated channel alpha 2	2,6075	0,025<br>3
239114	Il17d	interleukin 17D	2,6017	0,026<br>1
12389	Cav1	caveolin 1, caveolae protein	2,6117	0,026<br>2
12834	Col6a2	collagen, type VI, alpha 2	2,5924	0,026
13482	Dpp4	dipeptidylpeptidase 4	2,6034	0,025<br>9
244923	Klhl31	kelch-like 31 (Drosophila)	2,5703	0,026<br>8
11459	Acta1	actin, alpha 1, skeletal muscle	2,6017	0,026<br>7
211480	Kcnj14	potassium inwardly-rectifying channel, subfamily J, member 14	2,6884	0,026<br>8
12815	Col11a2	collagen, type XI, alpha 2	2,5905	0,027<br>6
15223	Foxj1	forkhead box J1	2,5385	0,028<br>6
17022	Lum	lumican	2,5526	0,028<br>6
12835	Col6a3	collagen, type VI, alpha 3	2,5771	0,028<br>5
11551	Adra2a	adrenergic receptor, alpha 2a	2,5268	0,028<br>4
329941	Col8a2	collagen, type VIII, alpha 2	2,5388	0,028<br>5
13038	Ctsk	cathepsin K	2,5566	0,028<br>8
26878	B3galt2	UDP-Gal:betaGlcNAc beta 1,3-galactosyltransferase, polypeptide 2	2,54	0,028<br>9
320563	Islr2	immunoglobulin superfamily containing leucine-rich repeat 2	2,7171	0,028<br>9
17395	Mmp9	matrix metallopeptidase 9	2,5449	0,029<br>1
18619	Penk	preproenkephalin	2,5451	0,029
23967	Osr1	odd-skipped related 1 (Drosophila)	2,5474	0,029<br>7
74342	Lrrtm1	leucine rich repeat transmembrane neuronal 1	2,5185	0,029<br>7
406186	Olfr142	olfactory receptor 142	2,6741	0,029<br>7
12642	Ch25h	cholesterol 25-hydroxylase	2,4873	0,031<br>9

11829	Aqp4	aquaporin 4	2,516	0,0321
12870	Cp	ceruloplasmin	2,5221	0,0325
226278	Prlhr	prolactin releasing hormone receptor	2,6642	0,0324
20750	Spp1	secreted phosphoprotein 1	2,5246	0,0335
13730	Emp1	epithelial membrane protein 1	2,4814	0,0343
116847	Prelp	proline arginine-rich end leucine-rich repeat	2,4648	0,0351
68631	Cryl1	crystallin, lambda 1	2,445	0,0353
216350	Tspan8	tetraspanin 8	2,4445	0,0363
107581	Col16a1	collagen, type XVI, alpha 1	2,4454	0,0365
71145	Scara5	scavenger receptor class A, member 5 (putative)	2,4389	0,0364
218215	Rnf144b	ring finger protein 144B	2,4658	0,0364
67606	Fibin	fin bud initiation factor homolog (zebrafish)	2,4629	0,0363
192166	Sardh	sarcosine dehydrogenase	2,4235	0,0369
16859	Lgals9	lectin, galactose binding, soluble 9	2,4375	0,0367
17470	Cd200	CD200 antigen	2,4547	0,0371
109042	Prkcdbp	protein kinase C, delta binding protein	2,4282	0,0373
11600	Angpt1	angiopoietin 1	2,4703	0,0376
114301	Palmd	palmdelphin	2,4497	0,0377
53945	Slc40a1	solute carrier family 40 (iron-regulated transporter), member 1	2,4048	0,0376
338320	Mia2	melanoma inhibitory activity 2	2,5517	0,0374
14004	Chchd2	coiled-coil-helix-coiled-coil-helix domain containing 2	2,4331	0,0373
14395	Gabra2	gamma-aminobutyric acid (GABA) A receptor, subunit alpha 2	2,519	0,0387
12832	Col5a2	collagen, type V, alpha 2	2,4177	0,0387
16870	Lhx2	LIM homeobox protein 2	2,4201	0,0389
73835	Ifitm5	interferon induced transmembrane protein 5	2,4327	0,0393
72169	Trim29	tripartite motif-containing 29	2,4836	0,0405
239766	Rtp1	receptor transporter protein 1	2,3863	0,040

				5
22402	Wisp1	WNT1 inducible signaling pathway protein 1	2,3934	0,0405
243771	Parp12	poly (ADP-ribose) polymerase family, member 12	2,3813	0,0405
12259	C1qa	complement component 1, q subcomponent, alpha polypeptide	2,385	0,0405
12831	Col5a1	collagen, type V, alpha 1	2,3832	0,0405
12306	Anxa2	annexin A2	2,4058	0,0409
259086	Olfr609	olfactory receptor 609	2,6194	0,0411
16682	Krt4	keratin 4	2,3786	0,0416
11981	Atp9a	ATPase, class II, type 9A	2,3707	0,0413
18133	Nov	nephroblastoma overexpressed gene	2,3624	0,0413
68775	Atp6v1c2	ATPase, H+ transporting, lysosomal V1 subunit C2	2,3572	0,0414
11749	Anxa6	annexin A6	2,374	0,0414
26427	Creb3l1	cAMP responsive element binding protein 3-like 1	2,3902	0,0413
244179	Ubqlnl	ubiquilin-like	2,3786	0,0411
13733	Emr1	EGF-like module containing, mucin-like, hormone receptor-like sequence 1	2,3595	0,0417
12741	Cldn5	claudin 5	2,4016	0,0418
57814	Kcne4	potassium voltage-gated channel, Isk-related subfamily, gene 4	2,3708	0,0418
228543	Rhov	ras homolog gene family, member V	2,3587	0,0421
258361	Olfr495	olfactory receptor 495	2,397	0,0433
234964	Ccdc67	coiled-coil domain containing 67	2,3939	0,0435
12405	Cbln2	cerebellin 2 precursor protein	2,358	0,0438
320712	Abi3bp	ABI gene family, member 3 (NESH) binding protein	2,3873	0,044
237213	Glra2	glycine receptor, alpha 2 subunit	2,5082	0,0446
259119	Olfr578	olfactory receptor 578	2,3555	0,0444
14241	Foxl1	forkhead box L1	2,4117	0,0448
319713	Ablim3	actin binding LIM protein family, member 3	2,3996	0,045
23876	Fbln5	fibulin 5	2,3476	0,0448
216643	Gabrp	gamma-aminobutyric acid (GABA) A receptor, pi	2,3732	0,044

				8
16521	Kcnj5	potassium inwardly-rectifying channel, subfamily J, member 5	2,3747	0,0447
56338	Txnip	thioredoxin interacting protein	2,3305	0,0449
66864	Clec14a	C-type lectin domain family 14, member a	2,3277	0,0464
192734	AI646023	expressed sequence AI646023	2,3422	0,0464
16545	Kera	keratocan	2,3846	0,0462
72393	Faim2	Fas apoptotic inhibitory molecule 2	2,3246	0,0464
208098	Panx3	pannexin 3	2,4504	0,0464
66141	Ifitm3	interferon induced transmembrane protein 3	2,3032	0,0465
21345	Tagln	transgelin	2,3061	0,0464
20183	Rxrg	retinoid X receptor gamma	2,3055	0,0465
16598	Klf2	Kruppel-like factor 2 (lung)	2,3048	0,0464
75379	4930599N23Rik	RIKEN cDNA 4930599N23 gene	2,3861	0,047
12978	Csf1r	colony stimulating factor 1 receptor	2,2875	0,047
22772	Zic2	zinc finger protein of the cerebellum 2	2,4039	0,0469
20834	Znrf4	zinc and ring finger 4	2,458	0,0468
12508	Cd53	CD53 antigen	2,3094	0,0467
56710	Dbc1	deleted in bladder cancer 1 (human)	2,292	0,0467
68303	Fam114a1	family with sequence similarity 114, member A1	2,3013	0,0471
11815	Apod	apolipoprotein D	2,2898	0,048
53870	Cntn6	contactin 6	2,3367	0,0481
23796	Aplnr	apelin receptor	2,2923	0,0479
50781	Dkk3	dickkopf homolog 3 (Xenopus laevis)	2,3137	0,0479
18331	Olfr32	olfactory receptor 32	2,5132	0,0479
71233	Enkur	enkurin, TRPC channel interacting protein	2,2984	0,0479
72693	Zcchc12	zinc finger, CCHC domain containing 12	2,3699	0,0478
18166	Npy1r	neuropeptide Y receptor Y1	2,3196	0,0477

Supplementary Table IV. Differentially Expressed Genes (DEG) in OE E14.5 vs. OPL 11.5. Raw EPI data subtracted of the MES data. The gene symbols, gene names, Entrez gene ID, fold-change and significance are reported. DEGs are ranked according to the "fold-change". The non-annotated probes and the *OR* genes have been eliminated.

probe	gene ID	symbol	gene name	M	FDR
6887661	74742	5830411J07Rik	RIKEN cDNA 5830411J07 gene	-4,7547	0,0054
6899100	17261	Mef2d	myocyte enhancer factor 2D	-4,5338	0,0085
6840199	210126	Lpp	LIM domain containing preferred translocation partner in lipoma	-4,2936	0,0092
6998072	19823	Rnf7	ring finger protein 7	-3,8283	0,015
6753536	320138	A130050O07Rik	RIKEN cDNA A130050O07 gene	-3,6939	0,0158
6964377	319527	B230325K18Rik	RIKEN cDNA B230325K18 gene	-3,6349	0,017
6926344	69902	Mrto4	MRT4, mRNA turnover 4, homolog (S. cerevisiae)	-3,5704	0,0179
6870583	26930	Ppnr	per-pentamer repeat gene	-3,6277	0,0183
7015993	22294	Uxt	ubiquitously expressed transcript	-3,5627	0,0182
6815491	68189	5330431K02Rik	RIKEN cDNA 5330431K02 gene	-3,5109	0,0181
6860035	448986	Pnet-ps	prenatal ethanol induced mRNA, pseudogene	-3,4621	0,0195
6989101	414109	9830163H01Rik	RIKEN cDNA 9830163H01 gene	-3,1012	0,0334
6895460	80892	Zfhx4	zinc finger homeodomain 4	-3,0306	0,036
7013844	319963	9330119M13Rik	RIKEN cDNA 9330119M13 gene	-2,9064	0,0408
6846799	320756	9330155M09Rik	RIKEN cDNA 9330155M09 gene	-3,0642	0,0429
6889440	320687	A130004G07Rik	RIKEN cDNA A130004G07 gene	-2,8244	0,0441
6962951	319522	D930046H04Rik	RIKEN cDNA D930046H04 gene	-2,8115	0,0458
6983790	319911	D830024N08Rik	RIKEN cDNA D830024N08 gene	-2,7145	0,0476

Supplementary Table V. Differentially Expressed Genes (DEG) in VNO E14.5 vs. OPL 11.5. Raw EPI data subtracted of the MES data. The gene symbols, gene names, Entrez gene ID, fold-change and significance are reported. DEGs are ranked according to the "fold-change". The non-annotated probes and the *OR* genes have been eliminated.

entrez ID	symbol	gene name	M	FDR
15891	Ibsp	integrin binding sialoprotein	8,319	0
18843	Plunc	palate, lung, and nasal epithelium associated	7,9589	0
66695	Aspn	asporin	7,1264	0
19695	Reg3g	regenerating islet-derived 3 gamma	6,466	0
13107	Cyp2f2	cytochrome P450, family 2, subfamily f, polypeptide 2	6,2299	0
12409	Cbr2	carbonyl reductase 2	6,075	0
11475	Acta2	actin, alpha 2, smooth muscle, aorta	5,9577	0
66957	Serpinb11	serine (or cysteine) peptidase inhibitor, clade B (ovalbumin), member 11	5,7992	0
13108	Cyp2g1	cytochrome P450, family 2, subfamily g, polypeptide 1	5,6303	0
13179	Dcn	decorin	5,5046	0
16840	Lect1	leukocyte cell derived chemotaxin 1	5,3702	9,00E-04
21924	Tnnc1	troponin C, cardiac/slow skeletal	5,1952	8,00E-04
244180	E030002O03Rik	RIKEN cDNA E030002O03 gene	5,0783	7,00E-04
21826	Thbs2	thrombospondin 2	4,9636	7,00E-04
16667	Krt17	keratin 17	4,9583	6,00E-04
19285	Ptrf	polymerase I and transcript release factor	4,9515	6,00E-04
55990	Fmo2	flavin containing monooxygenase 2	4,6778	6,00E-04
16952	Anxa1	annexin A1	4,6554	5,00E-04
170574	Sp7	Sp7 transcription factor 7	4,6282	5,00E-04
20256	Clec11a	C-type lectin domain family 11, member a	4,6037	5,00E-04
116872	Serpinb7	serine (or cysteine) peptidase inhibitor, clade B, member 7	4,6	5,00E-04
11522	Adh1	alcohol dehydrogenase 1 (class I)	4,5541	4,00E-04
16545	Kera	keratocan	4,502	4,00E-04
246048	Chodl	chondrolectin	4,4821	4,00E-04
208098	Panx3	pannexin 3	4,4815	4,00E-04
12842	Col1a1	collagen, type I, alpha 1	4,4439	4,00E-04
13661	Ehf	ets homologous factor	4,3905	7,00E-04
18295	Ogn	osteoglycin	4,372	7,00E-04
21923	Tnc	tenascin C	4,3651	7,00E-04
11459	Acta1	actin, alpha 1, skeletal muscle	4,3085	6,00E-04
20753	Sprr1a	small proline-rich protein 1A	4,428	6,00E-04
17386	Mmp13	matrix metallopeptidase 13	4,2758	6,00E-04
12818	Col14a1	collagen, type XIV, alpha 1	4,2776	6,00E-04
20750	Spp1	secreted phosphoprotein 1	4,2134	6,00E-04
21952	Tnni1	troponin I, skeletal, slow 1	4,1747	5,00E-04
66166	S100a14	S100 calcium binding protein A14	4,229	5,00E-04
16669	Krt19	keratin 19	4,1039	5,00E-04
12839	Col9a1	collagen, type IX, alpha 1	4,0467	0,001
18979	Pon1	paraoxonase 1	4,0517	0,001
13717	Eln	elastin	4,004	9,00E-04

12833	Col6a1	collagen, type VI, alpha 1	3,9862	9,00E-04
73835	Ifitm5	interferon induced transmembrane protein 5	4,0228	9,00E-04
11752	Anxa8	annexin A8	3,9369	9,00E-04
13732	Emp3	epithelial membrane protein 3	3,8846	0,001
16948	Lox	lysyl oxidase	3,8826	0,001
20379	Sfrp4	secreted frizzled-related protein 4	3,8954	0,0012
17928	Myog	myogenin	3,794	0,0012
22117	Tst	thiosulfate sulfurtransferase, mitochondrial	3,7899	0,0011
18121	Nog	noggin	3,8771	0,0011
14261	Fmo1	flavin containing monooxygenase 1	3,786	0,0011
17180	Matn1	matrilin 1, cartilage matrix protein	3,7559	0,0011
213436	Zcchc5	zinc finger, CCHC domain containing 5	3,7582	0,001
117167	Steap4	STEAP family member 4	3,6862	0,0012
13849	Ephx1	epoxide hydrolase 1, microsomal	3,6717	0,0011
19132	Prph	peripherin	3,6111	0,0011
56429	Dpt	dermatopontin	3,6106	0,0011
27047	Omd	osteomodulin	3,5998	0,0011
237831	Slc13a5	solute carrier family 13 (sodium-dependent citrate transporter), member 5	3,6316	0,0012
11828	Aqp3	aquaporin 3	3,5957	0,0012
107250	Kazald1	Kazal-type serine peptidase inhibitor domain 1	3,5974	0,0012
14178	Fgf7	fibroblast growth factor 7	3,6034	0,0012
20888	Sult1c1	sulfotransferase family, cytosolic, 1C, member 1	3,5615	0,0014
11472	Actn2	actinin alpha 2	3,5682	0,0014
18212	Ntrk2	neurotrophic tyrosine kinase, receptor, type 2	3,5696	0,0015
17901	Myl1	myosin, light polypeptide 1	3,5174	0,0015
12350	Car3	carbonic anhydrase 3	3,5575	0,0015
12373	Casq2	calsequestrin 2	3,5126	0,0015
81799	C1qtnf3	C1q and tumor necrosis factor related protein 3	3,5115	0,0015
16870	Lhx2	LIM homeobox protein 2	3,5004	0,0016
11595	Acan	aggrecan	3,4659	0,0017
17907	Mylpf	myosin light chain, phosphorylatable, fast skeletal muscle	3,4515	0,0017
105785	Kdelr3	KDEL (Lys-Asp-Glu-Leu) endoplasmic reticulum protein retention receptor 3	3,4531	0,0016
70935	Speer4f	spermatogenesis associated glutamate (E)-rich protein 4f	3,6705	0,0016
16665	Krt15	keratin 15	3,4316	0,0016
17395	Mmp9	matrix metallopeptidase 9	3,4301	0,0018
53315	Sult1d1	sulfotransferase family 1D, member 1	3,4441	0,0019
21817	Tgm2	transglutaminase 2, C polypeptide	3,4274	0,0021
15375	Foxa1	forkhead box A1	3,4155	0,0021
12816	Col12a1	collagen, type XII, alpha 1	3,3891	0,0022
20259	Scin	scinderin	3,403	0,0023
13507	Dsc3	desmocollin 3	3,4065	0,0024
11600	Angpt1	angiopoietin 1	3,3645	0,0025
110935	Atp6v1b1	ATPase, H+ transporting, lysosomal V1 subunit B1	3,3527	0,0024
18378	Omp	olfactory marker protein	3,363	0,0024
16905	Lmna	lamin A	3,3328	0,0025
12835	Col6a3	collagen, type VI, alpha 3	3,3277	0,0024

18032	Nfix	nuclear factor I/X	3,2964	0,0029
20724	Serpinb5	serine (or cysteine) peptidase inhibitor, clade B, member 5	3,2907	0,0029
14859	Gsta3	glutathione S-transferase, alpha 3	3,2834	0,0029
74186	Ccdc3	coiled-coil domain containing 3	3,2605	0,0029
12577	Cdkn1c	cyclin-dependent kinase inhibitor 1C (P57)	3,2673	0,0029
74442	Sgms2	sphingomyelin synthase 2	3,2628	0,0029
258296	Olfr745	olfactory receptor 745	3,2682	0,0029
213043	Aox3l1	aldehyde oxidase 3-like 1	3,2372	0,003
20716	Serpina3n	serine (or cysteine) peptidase inhibitor, clade A, member 3N	3,2778	0,003
244923	Klhl31	kelch-like 31 (Drosophila)	3,2279	0,0032
67951	Tubb6	tubulin, beta 6	3,2335	0,0031
319875	Tmprss11bnl	transmembrane protease, serine 11b N terminal like	3,1997	0,0031
13809	Enpep	glutamyl aminopeptidase	3,1947	0,0031
58994	Smpd3	sphingomyelin phosphodiesterase 3, neutral	3,1939	0,0031
170798	AY036118	cDNA sequence AY036118	3,5388	0,0032
11676	Aldoc	aldolase C, fructose-bisphosphate	3,1832	0,0032
13038	Ctsk	cathepsin K	3,1828	0,0032
22268	Upk1b	uroplakin 1B	3,1774	0,0033
74145	F13a1	coagulation factor XIII, A1 subunit	3,1624	0,0034
12389	Cav1	caveolin 1, caveolae protein	3,1604	0,0034
56188	Fxyd1	FXYD domain-containing ion transport regulator 1	3,118	0,0037
70008	Ace2	angiotensin I converting enzyme (peptidyl-dipeptidase A) 2	3,1023	0,0038
239790	Ostn	osteocrin	3,1154	0,0038
54156	Egfl6	EGF-like-domain, multiple 6	3,094	0,0038
22270	Upk3a	uroplakin 3A	3,0881	0,0038
320712	Abi3bp	ABI gene family, member 3 (NESH) binding protein	3,0946	0,0037
12837	Col8a1	collagen, type VIII, alpha 1	3,0848	0,004
234964	Ccdc67	coiled-coil domain containing 67	3,0488	0,0046
11826	Aqp1	aquaporin 1	3,0495	0,0046
11529	Adh7	alcohol dehydrogenase 7 (class IV), mu or sigma polypeptide	3,051	0,0046
13522	Adam28	a disintegrin and metallopeptidase domain 28	3,0495	0,0046
207596	Thsd4	thrombospondin, type I, domain containing 4	3,0162	0,005
74342	Lrrtm1	leucine rich repeat transmembrane neuronal 1	3,0149	0,0054
12834	Col6a2	collagen, type VI, alpha 2	2,9851	0,0056
14238	Foxf2	forkhead box F2	3,0465	0,006
22402	Wisp1	WNT1 inducible signaling pathway protein 1	2,9505	0,006
13516	Epyc	epiphycan	2,9322	0,0063
114301	Palmd	palmdelphin	2,9363	0,0063
17183	Matn4	matrilin 4	2,9254	0,0064
14132	Fcgrt	Fc receptor, IgG, alpha chain transporter	2,9266	0,0064
66139	Tmem8c	transmembrane protein 8C	2,9123	0,0067
17112	Tm4sf1	transmembrane 4 superfamily member 1	2,8987	0,0068
15223	Foxj1	forkhead box J1	2,891	0,0071
110012	Gm16517	predicted gene, Gm16517	2,8384	0,0079

53870	Cntn6	contactin 6	2,8376	0,0079
68680	Fitm1	fat storage-inducing transmembrane protein 1	2,8442	0,0081
330216	Mblac1	metallo-beta-lactamase domain containing 1	2,9319	0,008
21824	Thbd	thrombomodulin	2,8138	0,008
11829	Aqp4	aquaporin 4	2,819	0,008
208890	Slc26a7	solute carrier family 26, member 7	2,7984	0,0079
259300	Ehd2	EH-domain containing 2	2,8024	0,008
11647	Alpl	alkaline phosphatase, liver/bone/kidney	2,7842	0,0081
21743	Inmt	indolethylamine N-methyltransferase	2,7751	0,0087
56401	Lepre1	leprecan 1	2,7559	0,0089
13346	Des	desmin	2,7648	0,0088
53945	Slc40a1	solute carrier family 40 (iron-regulated transporter), member 1	2,7302	0,0087
16682	Krt4	keratin 4	2,7343	0,0087
17181	Matn2	matrilin 2	2,7315	0,0087
12832	Col5a2	collagen, type V, alpha 2	2,7238	0,009
18858	Pmp22	peripheral myelin protein 22	2,7132	0,0093
258702	Olfr410	olfactory receptor 410	2,6896	0,0095
19220	Ptgfr	prostaglandin F receptor	2,701	0,0096
13512	Dsg3	desmoglein 3	2,708	0,0095
20309	Cxcl15	chemokine (C-X-C motif) ligand 15	2,6925	0,0098
12306	Anxa2	annexin A2	2,6855	0,0098
17022	Lum	lumican	2,6822	0,01
244416	Ppp1r3b	protein phosphatase 1, regulatory (inhibitor) subunit 3B	2,6628	0,0099
72169	Trim29	tripartite motif-containing 29	2,6562	0,0101
97440	B3gnt9-ps	UDP-GlcNAc:betaGal beta-1,3-N-acetylglucosaminyltransferase 9, pseudogene	2,6663	0,0102
99543	Olfml3	olfactomedin-like 3	2,6591	0,0102
12638	Cftr	cystic fibrosis transmembrane conductance regulator homolog	2,65	0,0111
11435	Chrna1	cholinergic receptor, nicotinic, alpha polypeptide 1 (muscle)	2,64	0,0112
26878	B3galt2	UDP-Gal:betaGlcNAc beta 1,3-galactosyltransferase, polypeptide 2	2,6346	0,0114
13730	Emp1	epithelial membrane protein 1	2,6063	0,0119
17203	Mc5r	melanocortin 5 receptor	2,6196	0,0118
18741	Pitx2	paired-like homeodomain transcription factor 2	2,6338	0,0119
20193	S100a1	S100 calcium binding protein A1	2,616	0,0119
12950	Hapln1	hyaluronan and proteoglycan link protein 1	2,634	0,0119
12180	Smyd1	SET and MYND domain containing 1	2,5939	0,0122
23876	Fbln5	fibulin 5	2,6057	0,0122
12772	Ccr2	chemokine (C-C motif) receptor 2	2,5975	0,0125
11749	Anxa6	annexin A6	2,5762	0,0125
13876	Erg	avian erythroblastosis virus E-26 (v-ets) oncogene related	2,583	0,0128
109042	Prkcdbp	protein kinase C, delta binding protein	2,5709	0,013
12491	Cd36	CD36 antigen	2,5717	0,013
19737	Rgs5	regulator of G-protein signaling 5	2,547	0,0139
259015	Olfr1038-ps	olfactory receptor 1038, pseudogene	2,6472	0,0138

14560	Gdf10	growth differentiation factor 10	2,5552	0,0139
14164	Fgf1	fibroblast growth factor 1	2,5293	0,0138
21345	Tagln	transgelin	2,543	0,0139
12643	Chad	chondroadherin	2,5715	0,0138
17533	Mrc1	mannose receptor, C type 1	2,5395	0,0139
63873	Trpv4	transient receptor potential cation channel, subfamily V, member 4	2,5332	0,0138
20308	Ccl9	chemokine (C-C motif) ligand 9	2,5461	0,0141
14184	Fgfr3	fibroblast growth factor receptor 3	2,5242	0,014
54420	Cldn8	claudin 8	2,5488	0,014
67606	Fibin	fin bud initiation factor homolog (zebrafish)	2,5163	0,0143
13193	Dcx	doublecortin	2,5107	0,0137
227753	Gsn	gelsolin	2,5023	0,0149
12505	Cd44	CD44 antigen	2,4828	0,0161
12821	Col17a1	collagen, type XVII, alpha 1	2,4778	0,0162
99899	Ifi44	interferon-induced protein 44	2,4821	0,0169
18573	Pde1a	phosphodiesterase 1A, calmodulin-dependent	2,4845	0,0169
114332	Lyve1	lymphatic vessel endothelial hyaluronan receptor 1	2,4834	0,0168
20324	Sdpr	serum deprivation response	2,5532	0,0169
23796	Aplnr	apelin receptor	2,4793	0,0168
107581	Col16a1	collagen, type XVI, alpha 1	2,4649	0,017
12789	Cnga2	cyclic nucleotide gated channel alpha 2	2,4598	0,0176
258767	Olfr1176	olfactory receptor 1176	2,4652	0,0175
231997	Fkbp14	FK506 binding protein 14	2,4656	0,0175
51801	Ramp1	receptor (calcitonin) activity modifying protein 1	2,4827	0,0174
68303	Fam114a1	family with sequence similarity 114, member A1	2,4427	0,0174
17313	Mgp	matrix Gla protein	2,4624	0,0176
228543	Rhov	ras homolog gene family, member V	2,4654	0,0179
66042	Sostdc1	sclerostin domain containing 1	2,4358	0,0178
77125	Il33	interleukin 33	2,4354	0,0177
23967	Osr1	odd-skipped related 1 (Drosophila)	2,4752	0,0177
18012	Neurod1	neurogenic differentiation 1	2,4451	0,0177
319713	Ablim3	actin binding LIM protein family, member 3	2,4137	0,0178
56338	Txnip	thioredoxin interacting protein	2,4235	0,018
75379	4930599N23Rik	RIKEN cDNA 4930599N23 gene	2,4345	0,0181
64103	Tnmd	tenomodulin	2,4089	0,0181
216725	Adamts2	a disintegrin-like and metallopeptidase (reprolysin type) with thrombospondin type 1 motif, 2	2,4051	0,0183
67896	Ccdc80	coiled-coil domain containing 80	2,4007	0,0186
12831	Col5a1	collagen, type V, alpha 1	2,4066	0,0186
69642	2310046A06Rik	RIKEN cDNA 2310046A06 gene	2,3949	0,019
108075	Ltbp4	latent transforming growth factor beta binding protein 4	2,3849	0,0193
51812	Mcrs1	microspherule protein 1	2,4971	0,0197
216350	Tspan8	tetraspanin 8	2,3798	0,0197
258564	Olfr1015	olfactory receptor 1015	2,3973	0,0199
66180	1110036O03Rik	RIKEN cDNA 1110036O03 gene	2,3653	0,0203
234356	Csgalnact1	chondroitin sulfate N-acetylgalactosaminyltransferase 1	2,3625	0,0203
20429	Shox2	short stature homeobox 2	2,3709	0,0202

17873	Gadd45b	growth arrest and DNA-damage-inducible 45 beta	2,3669	0,0206
17700	Mstn	myostatin	2,3478	0,0206
11622	Ahr	aryl-hydrocarbon receptor	2,3517	0,0207
20319	Sfrp2	secreted frizzled-related protein 2	2,344	0,0212
11684	Alox12	arachidonate 12-lipoxygenase	2,3423	0,0213
13506	Dsc2	desmocollin 2	2,3696	0,0212
211480	Kcnj14	potassium inwardly-rectifying channel, subfamily J, member 14	2,4518	0,0218
329941	Col8a2	collagen, type VIII, alpha 2	2,3334	0,0219
66864	Clec14a	C-type lectin domain family 14, member a	2,3439	0,0225
68631	Cryl1	crystallin, lambda 1	2,3221	0,0228
14241	Foxl1	forkhead box L1	2,3553	0,0231
71233	Enkur	enkurin, TRPC channel interacting protein	2,3242	0,0233
67776	Vwa5a	von Willebrand factor A domain containing 5A	2,3307	0,0233
57814	Kcne4	potassium voltage-gated channel, Isk-related subfamily, gene 4	2,3493	0,0234
63913	Fam129a	family with sequence similarity 129, member A	2,305	0,0238
14004	Chchd2	coiled-coil-helix-coiled-coil-helix domain containing 2	2,3181	0,0243
210622	Pamr1	peptidase domain containing associated with muscle regeneration 1	2,338	0,0242
338368	Fam109b	family with sequence similarity 109, member B	2,3178	0,0242
24084	Tekt2	tektin 2	2,2989	0,0245
13731	Emp2	epithelial membrane protein 2	2,2964	0,0245
21956	Tnnt2	troponin T2, cardiac	2,3052	0,0249
26427	Creb3l1	cAMP responsive element binding protein 3-like 1	2,308	0,025
20203	S100b	S100 protein, beta polypeptide, neural	2,3444	0,0254
52377	Rcn3	reticulocalbin 3, EF-hand calcium binding domain	2,2855	0,0255
67198	Spats2l	spermatogenesis associated, serine-rich 2-like	2,2846	0,0259
18619	Penk	preproenkephalin	2,2864	0,0259
16780	Lamb3	laminin, beta 3	2,285	0,0258
227618	Lrrc26	leucine rich repeat containing 26	2,2789	0,026
105349	Akr1c18	aldo-keto reductase family 1, member C18	2,2877	0,0261
12022	Barx1	BarH-like homeobox 1	2,3026	0,0262
19737	Rgs5	regulator of G-protein signaling 5	2,301	0,0265
269784	Cntn4	contactin 4	2,3043	0,0265
20723	Serpinb9	serine (or cysteine) peptidase inhibitor, clade B, member 9	2,281	0,0265
224055	Rtp2	receptor transporter protein 2	2,3041	0,0268
105298	Epdr1	ependymin related protein 1 (zebrafish)	2,2618	0,0268
13837	Epha3	Eph receptor A3	2,3365	0,0271
16155	Il10rb	interleukin 10 receptor, beta	2,2669	0,0272
54399	Bet1l	blocked early in transport 1 homolog (S. cerevisiae)-like	2,2583	0,027
243771	Parp12	poly (ADP-ribose) polymerase family, member 12	2,2531	0,0273
68659	Fam198b	family with sequence similarity 198, member B	2,2447	0,0274
18772	Pkp1	plakophilin 1	2,2469	0,0275
75472	1700009P17Rik	RIKEN cDNA 1700009P17 gene	2,2626	0,0275
16521	Kcnj5	potassium inwardly-rectifying channel, subfamily J, member 5	2,2693	0,0275

13482	Dpp4	dipeptidylpeptidase 4	2,2805	0,0274
239337	Adamts12	a disintegrin-like and metallopeptidase (reprolysin type) with thrombospondin type 1 motif, 12	2,2468	0,0275
17927	Myod1	myogenic differentiation 1	2,247	0,0275
59083	Fetub	fetuin beta	2,2396	0,0274
20725	Serpinb8	serine (or cysteine) peptidase inhibitor, clade B, member 8	2,2359	0,0275
20556	Slfn2	schlafen 2	2,2858	0,0275
218215	Rnf144b	ring finger protein 144B	2,2508	0,0277
16009	Igfbp3	insulin-like growth factor binding protein 3	2,2291	0,028
20893	Bhlhe40	basic helix-loop-helix family, member e40	2,2315	0,0279
13036	Ctsh	cathepsin H	2,2496	0,0279
12824	Col2a1	collagen, type II, alpha 1	2,2396	0,0279
209512	Taar2	trace amine-associated receptor 2	2,246	0,0281
74782	Glt8d2	glycosyltransferase 8 domain containing 2	2,2521	0,0284
50781	Dkk3	dickkopf homolog 3 (Xenopus laevis)	2,2549	0,0282
11449	Chrng	cholinergic receptor, nicotinic, gamma polypeptide	2,2108	0,0283
60596	Gucy1a3	guanylate cyclase 1, soluble, alpha 3	2,2365	0,0285
21687	Tek	endothelial-specific receptor tyrosine kinase	2,2045	0,0293
239766	Rtp1	receptor transporter protein 1	2,1989	0,0296
223272	Itgbl1	integrin, beta-like 1	2,2143	0,0301
16985	Lsp1	lymphocyte specific 1	2,19	0,0302
12671	Chrm3	cholinergic receptor, muscarinic 3, cardiac	2,3051	0,0303
109272	Mybpc1	myosin binding protein C, slow-type	2,1881	0,0303
13040	Ctss	cathepsin S	2,1724	0,0314
12741	Cldn5	claudin 5	2,1948	0,0314
75465	Dynlrb2	dynein light chain roadblock-type 2	2,2216	0,0314
246316	Lgi2	leucine-rich repeat LGI family, member 2	2,1943	0,0315
244954	Prss35	protease, serine, 35	2,1802	0,0316
12759	Clu	clusterin	2,1794	0,0316
69585	Hfe2	hemochromatosis type 2 (juvenile) (human homolog)	2,168	0,0316
67483	1700028P14Rik	RIKEN cDNA 1700028P14 gene	2,2145	0,0321
14587	Gfra3	glial cell line derived neurotrophic factor family receptor alpha 3	2,1665	0,032
107585	Dio3	deiodinase, iodothyronine type III	2,1971	0,032
18452	P4ha2	procollagen-proline, 2-oxoglutarate 4-dioxygenase (proline 4-hydroxylase), alpha II polypeptide	2,1731	0,0322
30878	Apln	apelin	2,1789	0,0323
66141	Ifitm3	interferon induced transmembrane protein 3	2,1675	0,0324
16859	Lgals9	lectin, galactose binding, soluble 9	2,1501	0,0331
21828	Thbs4	thrombospondin 4	2,1382	0,0347
239336	Rxfp3	relaxin family peptide receptor 3	2,2078	0,0346
13655	Egr3	early growth response 3	2,1535	0,0347
11551	Adra2a	adrenergic receptor, alpha 2a	2,164	0,0348
57442	Kcne3	potassium voltage-gated channel, Isk-related subfamily, gene 3	2,2155	0,035
109323	C1qtnf7	C1q and tumor necrosis factor related protein 7	2,2027	0,0352
258609	Olfr305	olfactory receptor 305	2,2702	0,0352

18186	Nrp1	neuropilin 1	2,1138	0,0371
192166	Sardh	sarcosine dehydrogenase	2,1191	0,0377
20405	Sh3gl1	SH3-domain GRB2-like 1	2,1093	0,038
56213	Htra1	HtrA serine peptidase 1	2,1018	0,038
21813	Tgfbr2	transforming growth factor, beta receptor II	2,101	0,0384
67917	Zcchc3	zinc finger, CCHC domain containing 3	2,0874	0,0385
406186	Olfr142	olfactory receptor 142	2,2661	0,0399
12508	Cd53	CD53 antigen	2,0872	0,04
23959	Nt5e	5' nucleotidase, ecto	2,1099	0,0399
75533	Nme5	non-metastatic cells 5, protein expressed in (nucleoside-diphosphate kinase)	2,0956	0,0406
11858	Rnd2	Rho family GTPase 2	2,1019	0,0407
259108	Olfr550	olfactory receptor 550	2,1633	0,0405
71145	Scara5	scavenger receptor class A, member 5 (putative)	2,0717	0,0406
27280	Phlda3	pleckstrin homology-like domain, family A, member 3	2,0819	0,0405
13390	Dlx1	distal-less homeobox 1	2,07	0,0404
214704	Iqub	IQ motif and ubiquitin domain containing	2,0748	0,0404
69693	Pof1b	premature ovarian failure 1B	2,0851	0,0406
12843	Col1a2	collagen, type I, alpha 2	2,0963	0,0405
21898	Tlr4	toll-like receptor 4	2,0596	0,0423
329659	E130311K13Rik	RIKEN cDNA E130311K13 gene	2,0636	0,0427
64074	Smoc2	SPARC related modular calcium binding 2	2,0651	0,044
11816	Apoe	apolipoprotein E	2,0458	0,0442
13733	Emr1	EGF-like module containing, mucin-like, hormone receptor-like sequence 1	2,0438	0,0446
107587	Osr2	odd-skipped related 2 (Drosophila)	2,0323	0,0453
320736	E130203B14Rik	RIKEN cDNA E130203B14 gene	2,0395	0,0459
239789	Gm606	predicted gene 606	2,0671	0,0459
20465	Sim2	single-minded homolog 2 (Drosophila)	2,0334	0,0463
19354	Rac2	RAS-related C3 botulinum substrate 2	2,0459	0,0464
20503	Slc16a7	solute carrier family 16 (monocarboxylic acid transporters), member 7	2,0474	0,0467
121021	Cspg4	chondroitin sulfate proteoglycan 4	2,02	0,0472
216616	Efemp1	epidermal growth factor-containing fibulin-like extracellular matrix protein 1	2,0224	0,0479
209195	Clic6	chloride intracellular channel 6	2,0254	0,0484
56843	Trpm5	transient receptor potential cation channel, subfamily M, member 5	2,0079	0,0484
20183	Rxrg	retinoid X receptor gamma	2,0168	0,0486
68792	Srpx2	sushi-repeat-containing protein, X-linked 2	2,0182	0,0487
22413	Wnt2	wingless-related MMTV integration site 2	2,0179	0,0487
231633	Tmem119	transmembrane protein 119	2,0177	0,0487
12053	Bcl6	B-cell leukemia/lymphoma 6	2,0257	0,0496
19227	Pthlh	parathyroid hormone-like peptide	2,015	0,0496
56277	Tmem45a	transmembrane protein 45a	2,0081	0,0497

Supplementary Table VI. Differentially Expressed Genes (DEG) in VNO E14.5 vs. OPL 11.5. Raw EPI data subtracted of the MES data. The gene symbols, gene names, Entrez gene ID, fold-change and significance are reported. DEGs are ranked according to the "fold-change". The non-annotated probes and the *OR* genes have been eliminated.

**probe**	**gene ID**	**symbol**	**gene name**	**M**	**FDR**
6922864	76455	2310067E19Rik	RIKEN cDNA 2310067E19 gene	-4,9009	0,0017
6964377	319527	B230325K18Rik	RIKEN cDNA B230325K18 gene	-4,2047	0,0031
6815491	68189	5330431K02Rik	RIKEN cDNA 5330431K02 gene	-3,2445	0,0126
6837413	21378	Tbrg3	transforming growth factor beta regulated gene 3	-3,197	0,0145
6887661	74742	5830411J07Rik	RIKEN cDNA 5830411J07 gene	-2,9288	0,0227
6753536	320138	A130050O07Rik	RIKEN cDNA A130050O07 gene	-2,9571	0,0232
7016827	319912	A630012P03Rik	RIKEN cDNA A630012P03 gene	-2,8408	0,0269
6959326	22718	Zfp60	zinc finger protein 60	-2,6314	0,0339
6822216	320268	B930095G15Rik	RIKEN cDNA B930095G15 gene	-2,4508	0,0445
6977778	21672	Prdx2	peroxiredoxin 2	-2,4614	0,0447
6860035	448986	Pnet-ps	prenatal ethanol induced mRNA, pseudogene	-2,4162	0,0483
6768075	12140	Fabp7	fatty acid binding protein 7, brain	-2,4223	0,0493
6779838	15126	Hba-x	hemoglobin X, alpha-like embryonic chain in Hba complex	-2,3843	0,0491

Supplementary Table VII *OR* genes up-regulated comparing OE E14.5 vs. OPL E11.5

Entrez Gene ID	Gene Symbol
258206	Olfr1264
406186	Olfr142
258609	Olfr305
18331	Olfr32
258361	Olfr495
259119	Olfr578
259086	Olfr609
259057	Olfr649
258387	Olfr720

Supplementary Table VIII. *OR* genes up-regulated comparing VNO E14.5 vs. OPL 11.5

Entrez Gene ID	Gene Symbol
258564	Olfr1015
259015	Olfr1038-ps
258767	Olfr1176
406186	Olfr142
258609	Olfr305
258702	Olfr410
259108	Olfr550
258296	Olfr745

Two *OR* genes (Olfr142 and Olfr305) that are common DEGs in the OE and in the VNO.

Supplementary Table IX. Genes up-regulated in MES adjacent to OE E14.5 vs. OPL 11.5. The gene symbols, gene names, Entrez gene ID, fold-change and significance are reported. DEGs are ranked according to the "fold-change". The non-annotated probes have been eliminated.

log2_FC	P value	Gene Name	Symbol	Entrez ID
7,020926	0	asporin	Aspn	66695
6,878321	0	integrin binding sialoprotein	Ibsp	15891
6,333516	0	UDP glucuronosyltransferase 2 family, polypeptide A1 /// UDP glucuronosyltransferase 2 family, polypeptide A2	Ugt2a1 /// Ugt2a2	94215 /// 552899
5,740818	0	cytochrome P450, family 2, subfamily g, polypeptide 1	Cyp2g1	13108
4,6878	0	sulfotransferase family 1E, member 1	Sult1e1	20860

4,506353	0	thrombospondin 2	Thbs2	21826
4,257045	0	matrix Gla protein	Mgp	17313
4,235144	0	decorin	Dcn	13179
4,221623	0	leukocyte cell derived chemotaxin 1	Lect1	16840
4,110293	0	carboxypeptidase A3, mast cell	Cpa3	12873
4,092956	0	alcohol dehydrogenase 1 (class I)	Adh1	11522
4,0613	0	sulfotransferase family, cytosolic, 1C, member 1	Sult1c1	20888
4,056491	0	pannexin 3	Panx3	208098
4,030325	0	single-minded homolog 1 (Drosophila)	Sim1	20464
3,92139	0	aggrecan	Acan	11595
3,871971	0	follistatin-like 5	Fstl5	213262
3,818071	0	chondrolectin	Chodl	246048
3,708396	0	secreted phosphoprotein 1	Spp1	20750
3,700873	0	contactin 4	Cntn4	269784
3,662004	0	regenerating islet-derived 3 gamma	Reg3g	19695
3,599462	0	matrilin 1, cartilage matrix protein	Matn1	17180
3,534496	0	solute carrier family 27 (fatty acid transporter), member 2	Slc27a2	26458
3,450084	0	aldehyde oxidase 3-like 1	Aox3l1	213043
3,442222	0	matrix metallopeptidase 13	Mmp13	17386
3,349603	0	carbonyl reductase 2	Cbr2	12409
3,317606	0	annexin A1	Anxa1	16952
3,30187	0	acyl-CoA synthetase medium-chain family member 4	Acsm4	233801
3,290533	0	cytochrome P450, family 2, subfamily f, polypeptide 2	Cyp2f2	13107
3,259806	0	ermin, ERM-like protein	Ermn	77767
3,180495	0	G protein-coupled receptor 126	Gpr126	215798
3,158429	0	aquaporin 1	Aqp1	11826
3,137965	0	E74-like factor 5	Elf5	13711
3,102837	0	sodium channel, voltage-gated, type IX, alpha	Scn9a	20274
3,091725	0	osteoglycin	Ogn	18295
3,072179	0	matrix metallopeptidase 9	Mmp9	17395
3,0613	0	SV2 related protein homolog (rat)-like	Svopl	320590
3,052895	0	matrilin 4	Matn4	17183
3,025618	0	periostin, osteoblast specific factor	Postn	50706
3,010425	0	serine/threonine kinase 32A	Stk32a	269019
2,993092	0	palate, lung, and nasal epithelium associated	Plunc	18843
2,990796	0	zinc finger, CCHC domain containing 5	Zcchc5	213436
2,952322	0	collagen, type XIV, alpha 1	Col14a1	12818
2,925769	0	tripartite motif-containing 66	Trim66	330627
2,912673	0	cadherin 12	Cdh12	215654
2,910502	0	sulfotransferase family 1D, member 1	Sult1d1	53315
2,894322	0	keratin 1	Krt1	16678
2,891108	0	C-type lectin domain family 11, member a	Clec11a	20256
2,874084	0	ets homologous factor	Ehf	13661
2,867752	0	alkaline phosphatase, liver/bone/kidney	Alpl	11647
2,841663	0	trace amine-associated receptor 7D /// trace amine-associated receptor 7E /// trace amine-associated receptor 7A /// trace amine-associated receptor 7B /// trace amine-associated receptor 7F	Taar7d /// Taar7e /// Taar7a /// Taar7b /// Taar7f	435206 /// 276742 /// 215856 /// 209517 /// 435207
2,832385	0	testis expressed gene 15	Tex15	104271
2,798868	0	Sp7 transcription factor 7	Sp7	170574
2,771027	0	glutamyl aminopeptidase	Enpep	13809
2,743714	0	flavin containing monooxygenase 1	Fmo1	14261

2,669327	0	sphingomyelin synthase 2	Sgms2	74442
2,663831	0	elastin	Eln	13717
2,647467	0	fibroblast growth factor 7	Fgf7	14178
2,642054	0	coiled-coil domain containing 3	Ccdc3	74186
2,630394	0	nescient helix loop helix 1	Nhlh1	18071
2,617941	0	serine (or cysteine) peptidase inhibitor, clade B (ovalbumin), member 11	Serpinb11	66957
2,558942	0	actin, alpha 2, smooth muscle, aorta	Acta2	11475
2,517875	0	solute carrier family 13 (sodium-dependent citrate transporter), member 5	Slc13a5	237831
2,515398	0	3'-phosphoadenosine 5'-phosphosulfate synthase 2	Papss2	23972
2,512102	0	serine (or cysteine) peptidase inhibitor, clade B, member 7	Serpinb7	116872
2,483581	0	secreted frizzled-related protein 4	Sfrp4	20379
2,482775	0	B-cell leukemia/lymphoma 6	Bcl6	12053
2,478748	0	ATP-binding cassette, sub-family A (ABC1), member 8a	Abca8a	217258
2,475535	0	paraoxonase 1	Pon1	18979
2,454032	0	nuclear factor I/X	Nfix	18032
2,451662	0	sphingomyelin phosphodiesterase 3, neutral	Smpd3	58994
2,441438	0	neurexin I	Nrxn1	18189
2,438307	0	predicted gene, ENSMUSG00000067698 /// RIKEN cDNA 5031410I06 gene /// spermatogenesis associated glutamate (E)-rich protein 4a	ENSMUSG00000067698 /// 5031410I06Rik /// Speer4a	434689 /// 381622 /// 75657
2,423526	0	potassium large conductance calcium-activated channel, subfamily M, alpha member 1	Kcnma1	16531
2,41889	0	polymerase I and transcript release factor	Ptrf	19285
2,414268	0	lipase, member I	Lipi	320355
2,400492	0	collagen, type XII, alpha 1	Col12a1	12816
2,39973	0	CD44 antigen	Cd44	12505
2,377819	0	creatine kinase, mitochondrial 1, ubiquitous	Ckmt1	12716
2,375572	0	odd-skipped related 1 (Drosophila)	Osr1	23967
2,368849	0	ABI gene family, member 3 (NESH) binding protein	Abi3bp	320712
2,363642	0	caveolin 1, caveolae protein	Cav1	12389
2,362158	0	synaptosomal-associated protein 25	Snap25	20614
2,334971	0	mal, T-cell differentiation protein 2	Mal2	105853
2,316169	0	CD36 antigen	Cd36	12491
2,306859	0	epithelial membrane protein 3	Emp3	13732
2,296191	0,0001	interferon induced transmembrane protein 5	Ifitm5	73835
2,291945	0	ATPase, Na+/K+ transporting, beta 1 polypeptide	Atp1b1	11931
2,289122	0,0001	Purkinje cell protein 4	Pcp4	18546
2,266038	0,0001	integrin, beta-like 1	Itgbl1	223272
2,266038	0,0001	aquaporin 3	Aqp3	11828
2,264651	0,0001	RIKEN cDNA E030002O03 gene	E030002O03Rik	244180
2,259116	0,0001	keratin 23	Krt23	94179
2,254289	0,0001	tenascin C	Tnc	21923
2,235824	0,0001	dermatopontin	Dpt	56429
2,233786	0,0001	collagen, type VI, alpha 1	Col6a1	12833
2,231752	0,0001	CD55 antigen	Cd55	13136
2,231752	0,0001	scinderin	Scin	20259
2,231075	0,0001	glial cell line derived neurotrophic factor family receptor alpha 2	Gfra2	14586
2,222296	0,0001	activating transcription factor 5	Atf5	107503
2,213571	0,0001	flavin containing monooxygenase 2	Fmo2	55990
2,212902	0,0001	N-myc downstream regulated gene 1	Ndrg1	17988

2,18968	0,0001	cholesterol 25-hydroxylase	Ch25h	12642
2,188365	0,0001	sema domain, immunoglobulin domain (Ig), short basic domain, secreted, (semaphorin) 3D	Sema3d	108151
2,18705	0,0001	collagen, type VIII, alpha 1	Col8a1	12837
2,176577	0,0001	cAMP responsive element binding protein 3-like 1	Creb3l1	26427
2,173319	0,0001	carboxypeptidase A6	Cpa6	329093
2,172669	0,0001	Rho GDP dissociation inhibitor (GDI) gamma	Arhgdig	14570
2,172018	0,0001	S100 calcium binding protein A14	S100a14	66166
2,171368	0,0001	tenomodulin	Tnmd	64103
2,170719	0,0001	deiodinase, iodothyronine type III	Dio3	107585
2,155213	0,0001	sparc/osteonectin, cwcv and kazal-like domains proteoglycan 1	Spock1	20745
2,148161	0,0001	odd-skipped related 2 (Drosophila)	Osr2	107587
2,144967	0,0001	matrilin 3	Matn3	17182
2,142417	0,0001	prostaglandin F receptor	Ptgfr	19220
2,139871	0,0001	collagen, type VI, alpha 3	Col6a3	12835
2,137965	0,0001	EGF-like-domain, multiple 6	Egfl6	54156
2,117787	0,0001	nephroblastoma overexpressed gene	Nov	18133
2,113411	0,0001	hyaluronan and proteoglycan link protein 1	Hapln1	12950
2,10036	0,0001	leucine rich repeat and Ig domain containing 2	Lingo2	242384
2,081308	0,0001	regulator of G-protein signaling 5	Rgs5	19737
2,079478	0,0001	olfactory receptor 24	Olfr24	18322
2,07765	0,0001	receptor transporter protein 1	Rtp1	239766
2,057091	0,0002	olfactory receptor 1475 /// olfactory receptor 1474 /// olfactory receptor 1471 /// olfactory receptor 1472	Olfr1475 /// Olfr1474 /// Olfr1471 /// Olfr1472	258298 /// 258123 /// 258231 /// 258685
2,056491	0,0001	epoxide hydrolase 1, microsomal	Ephx1	13849
2,054692	0,0001	elongation factor RNA polymerase II-like 3	Ell3	269344
2,026205	0,0001	potassium voltage-gated channel, Isk-related subfamily, gene 4	Kcne4	57814
2,022097	0,0002	calsyntenin 2	Clstn2	64085
2,02034	0,0002	N-terminal EF-hand calcium binding protein 1	Necab1	69352
2,01917	0,0002	myelin transcription factor 1-like	Myt1l	17933
2,016833	0,0002	connective tissue growth factor	Ctgf	14219
2,011588	0,0002	small proline-rich protein 1A	Sprr1a	20753
2,004045	0,0002	otoraplin	Otor	57329
2,003467	0,0002	transient receptor potential cation channel, subfamily V, member 4	Trpv4	63873
1,990796	0,0002	cadherin 8	Cdh8	12564
1,98736	0,0002	ELAV (embryonic lethal, abnormal vision, Drosophila)-like 3 (Hu antigen C)	Elavl3	15571
1,975964	0,0002	thrombospondin, type I, domain containing 7B	Thsd7b	210417
1,975964	0,0002	C1q and tumor necrosis factor related protein 3	C1qtnf3	81799
1,972563	0,0002	alcohol dehydrogenase 7 (class IV), mu or sigma polypeptide	Adh7	11529
1,971431	0,0002	KDEL (Lys-Asp-Glu-Leu) endoplasmic reticulum protein retention receptor 3	Kdelr3	105785
1,957916	0,0002	stefin A3	Stfa3	20863
1,957916	0,0002	phosphate regulating gene with homologies to endopeptidases on the X chromosome (hypophosphatemia, vitamin D resistant rickets)	Phex	18675
1,954557	0,0002	glutamate receptor, ionotropic, kainate 1	Grik1	14805
1,953998	0,0002	nuclear receptor binding protein 2	Nrbp2	223649
1,946194	0,0002	angiotensin I converting enzyme (peptidyl-dipeptidase A) 2	Ace2	70008

1,945082	0,0002	Ca2+-dependent secretion activator	Cadps	27062
1,935117	0,0002	SPARC related modular calcium binding 2	Smoc2	64074
1,932361	0,0002	osteomodulin	Omd	27047
1,926317	0,0002	coagulation factor XIII, A1 subunit	F13a1	74145
1,921937	0,0002	aldehyde dehydrogenase family 1, subfamily A1	Aldh1a1	11668
1,91648	0,0002	carboxylesterase 3	Ces3	104158
1,913216	0,0003	basic helix-loop-helix family, member e41	Bhlhe41	79362
1,905088	0,0003	microtubule associated monoxygenase, calponin and LIM domain containing 2	Mical2	320878
1,901311	0,0003	neurogenic differentiation 1	Neurod1	18012
1,899695	0,0003	growth differentiation factor 10	Gdf10	14560
1,899695	0,0003	transient receptor potential cation channel, subfamily M, member 5	Trpm5	56843
1,898081	0,0003	leucine zipper protein 2	Luzp2	233271
1,894322	0,0003	CDC42 effector protein (Rho GTPase binding) 3	Cdc42ep3	260409
1,892714	0,0003	matrilin 2	Matn2	17181
1,8847	0,0003	a disintegrin and metallopeptidase domain 28	Adam28	13522
1,8864	0,0092	early B-cell factor 2	Ebf2	13592
1,883635	0,0003	chondroitin sulfate N-acetylgalactosaminyltransferase 1	Csgalnact1	234356
1,881507	0,0003	piccolo (presynaptic cytomatrix protein)	Pclo	26875
1,874613	0,0003	spondin 1, (f-spondin) extracellular matrix protein	Spon1	233744
1,86986	0,0003	angiopoietin 1	Angpt1	11600
1,8604	0,0003	RIKEN cDNA 8030451F13 gene	8030451F13Rik	109272
1,858829	0,0003	STEAP family member 4	Steap4	117167
1,85726	0,0003	stathmin-like 2	Stmn2	20257
1,854649	0,0003	RIKEN cDNA 4932418E24 gene	4932418E24Rik	329366
1,847881	0,0003	chloride intracellular channel 6	Clic6	209195
1,846324	0,0003	adenylate cyclase 2	Adcy2	210044
1,843733	0,0003	osteocrin	Ostn	239790
1,842698	0,0003	troponin C, cardiac/slow skeletal	Tnnc1	21924
1,841663	0,0003	RIKEN cDNA 9530019H20 gene	9530019H20Rik	320999
1,838048	0,0003	RAB25, member RAS oncogene family	Rab25	53868
1,831358	0,0003	collagen, type I, alpha 1	Col1a1	12842
1,829306	0,0003	lamin A	Lmna	16905
1,825721	0,0004	lectin, galactose binding, soluble 9	Lgals9	16859
1,822146	0,0004	histidine decarboxylase	Hdc	15186
1,818071	0,0003	calcium/calmodulin-dependent protein kinase ID	Camk1d	227541
1,817562	0,0004	grainyhead-like 1 (Drosophila)	Grhl1	195733
1,815022	0,0004	regulator of G-protein signaling 5	Rgs5	19737
1,81046	0,0004	keratin 17	Krt17	16667
1,802889	0,0004	fatty acid binding protein 7, brain	Fabp7	12140
1,802386	0,0004	glycoprotein m6a	Gpm6a	234267
1,798366	0,0004	a disintegrin-like and metallopeptidase (reprolysin type) with thrombospondin type 1 motif, 12	Adamts12	239337
1,791857	0,0004	olfactory receptor 495	Olfr495	258361
1,791358	0,0004	chondroadherin	Chad	12643
1,787368	0,0004	mitogen-activated protein kinase 10	Mapk10	26414
1,777443	0,0004	runt related transcription factor 1	Runx1	12394
1,776454	0,0004	mucin 4	Muc4	140474
1,772505	0,0005	melanocortin 5 receptor	Mc5r	17203
1,770535	0,0004	Fas apoptotic inhibitory molecule 2	Faim2	72393
1,765621	0,0005	cyclic nucleotide gated channel alpha 2	Cnga2	12789
1,765131	0,0005	visinin-like 1	Vsnl1	26950

1,76366	0,0004	very low density lipoprotein receptor	Vldlr	22359
1,75877	0,0004	collagen, type XI, alpha 1	Col11a1	12814
1,755844	0,0005	claudin 9	Cldn9	56863
1,750009	0,0005	forkhead box A1	Foxa1	15375
1,749038	0,0005	cDNA sequence BC048546	BC048546	232400
1,745648	0,0005	olfactory receptor 550	Olfr550	259108
1,744197	0,0005	leucine-rich repeat LGI family, member 2	Lgi2	246316
1,739372	0,0005	coagulation factor C homolog (Limulus polyphemus)	Coch	12810
1,736485	0,0006	keratin 15	Krt15	16665
1,734083	0,0005	lumican	Lum	17022
1,733603	0,0005	protein tyrosine phosphatase, receptor type, V	Ptprv	13924
1,732165	0,0005	Fc receptor, IgG, alpha chain transporter	Fcgrt	14132
1,731685	0,0005	keratin 18	Krt18	16668
1,730249	0,0005	Ca2+-dependent activator protein for secretion 2	Cadps2	320405
1,728813	0,0005	solute carrier family 44, member 5	Slc44a5	242259
1,727857	0,0005	monoamine oxidase B	Maob	109731
1,721183	0,0005	cysteine-rich protein 1 (intestinal)	Crip1	12925
1,717857	0,0005	neuropilin 1	Nrp1	18186
1,717382	0,0005	secreted frizzled-related protein 2	Sfrp2	20319
1,710284	0,0006	fibroblast growth factor 12	Fgf12	14167
1,708868	0,0006	serine/threonine kinase 32B	Stk32b	64293
1,708396	0,0006	cytoplasmic polyadenylation element binding protein 1	Cpeb1	12877
1,7051	0,0006	mast cell protease 2	Mcpt2	17225
1,70275	0,0006	cytochrome b reductase 1	Cybrd1	73649
1,70275	0,0006	sema domain, immunoglobulin domain (Ig), short basic domain, secreted, (semaphorin) 3C	Sema3c	20348
1,700404	0,0006	dual specificity phosphatase 10	Dusp10	63953
1,693854	0,0007	tubulin tyrosine ligase-like family, member 6	Ttll6	237930
1,692921	0,0006	leptin receptor	Lepr	16847
1,691523	0,0006	aldolase C, fructose-bisphosphate	Aldoc	11676
1,688264	0,0006	suppressor of cytokine signaling 2	Socs2	216233
1,685942	0,0007	transmembrane protein 45a	Tmem45a	56277
1,68455	0,0007	thrombospondin, type I, domain containing 4	Thsd4	207596
1,682233	0,0007	collagen, type VI, alpha 2	Col6a2	12834
1,663831	0,0008	tescalcin	Tesc	57816
1,663374	0,0007	peripheral myelin protein 22	Pmp22	18858
1,660635	0,0007	transglutaminase 2, C polypeptide	Tgm2	21817
1,658356	0,0007	V-set and transmembrane domain containing 2A	Vstm2a	211739
1,657445	0,0007	nucleolar protein 4	Nol4	319211
1,654263	0,0008	epithelial membrane protein 1	Emp1	13730
1,647016	0,0009	DNA segment, Chr 12, ERATO Doi 647, expressed	D12Ertd647e	52668
1,640254	0,0008	tandem C2 domains, nuclear	Tc2n	74413
1,638543	0,0407	neuregulin 1	Nrg1	211323
1,636212	0,0008	sparc/osteonectin, cwcv and kazal-like domains proteoglycan 2	Spock2	94214
1,629947	0,0009	wingless-related MMTV integration site 2	Wnt2	22413
1,628162	0,0008	paired-like homeodomain transcription factor 2	Pitx2	18741
1,628162	0,0008	met proto-oncogene	Met	17295
1,628162	0,0009	calmodulin-like 4	Calml4	75600
1,625934	0,0009	kelch-like 31 (Drosophila)	Klhl31	244923
1,625044	0,0009	Kazal-type serine peptidase inhibitor domain 1	Kazald1	107250
1,624154	0,0009	deleted in bladder cancer 1 (human)	Dbc1	56710
1,623265	0,0009	trinucleotide repeat containing 4	Tnrc4	78784

1,623265	0,0009	glycosyltransferase 8 domain containing 4	Glt8d4	232313
1,622376	0,0009	N-myc downstream regulated gene 2	Ndrg2	29811
1,618384	0,0009	interferon induced transmembrane protein 3	Ifitm3	66141
1,609112	0,0009	solute carrier family 26, member 7	Slc26a7	208890
1,606474	0,001	tumor protein D52	Tpd52	21985
1,65223	0,0055	neural cell adhesion molecule 2	Ncam2	17968
1,606035	0,0009	carbonic anhydrase 3	Car3	12350
1,604718	0,001	immunoglobulin-like domain containing receptor 1	Ildr1	106347
1,60165	0,001	CD9 antigen	Cd9	12527
1,59942	0,0029	peripherin	Prph	19132
1,59031	0,001	S100 protein, beta polypeptide, neural	S100b	20203
1,589008	0,001	RAS-like, estrogen-regulated, growth-inhibitor	Rerg	232441
1,58814	0,001	early growth response 1	Egr1	13653
1,585973	0,001	RALBP1 associated Eps domain containing protein 2	Reps2	194590
1,585107	0,0011	preproenkephalin 1	Penk1	18619
1,584241	0,0011	bone morphogenetic protein 5	Bmp5	12160
1,579922	0,0011	RIKEN cDNA D930020B18 gene	D930020B18Rik	216393
1,57906	0,0011	RNA binding motif protein 47	Rbm47	245945
1,576906	0,0011	sclerostin domain containing 1	Sostdc1	66042
1,575615	0,0011	CD200 antigen	Cd200	17470
1,573896	0,0011	dynein, axonemal, heavy chain 11	Dnahc11	13411
1,572179	0,0012	ribosomal protein L39-like	Rpl39l	68172
1,564478	0,0011	contactin associated protein-like 5B	Cntnap5b	241175
1,562346	0,0012	synaptic vesicle glycoprotein 2 b	Sv2b	64176
1,56192	0,0012	coagulation factor II (thrombin) receptor-like 1	F2rl1	14063
1,560983	0,0389	LIM homeobox protein 2	Lhx2	16870
1,560643	0,0012	synaptotagmin I	Syt1	20979
1,557667	0,0012	a disintegrin and metallopeptidase domain 12 (meltrin alpha)	Adam12	11489
1,556818	0,0012	beta-1,4-N-acetyl-galactosaminyl transferase 3	B4galnt3	330406
1,555121	0,0012	tenascin N	Tnn	329278
1,553426	0,0012	avian erythroblastosis virus E-26 (v-ets) oncogene related	Erg	13876
1,550465	0,0012	A kinase (PRKA) anchor protein 6	Akap6	238161
1,547932	0,0012	amyloid beta (A4) precursor protein binding, family A, member 1	Apba1	319924
1,546667	0,0012	Hedgehog-interacting protein	Hhip	15245
1,545824	0,0013	phosphatase, orphan 1 /// ABI gene family, member 3	Phospho1 /// Abi3	237928 /// 66610
1,541618	0,0013	HtrA serine peptidase 1	Htra1	56213
1,541198	0,0013	orthodenticle homolog 2 (Drosophila)	Otx2	18424
1,53952	0,0013	cadherin-like 26	Cdh26	381409
1,530322	0,0013	mitogen-activated protein kinase 13	Mapk13	26415
1,530322	0,0013	collagen, type XVI, alpha 1	Col16a1	107581
1,530322	0,0013	solute carrier family 24 (sodium/potassium/calcium exchanger), member 2	Slc24a2	76376
1,527408	0,0013	receptor transporter protein 2	Rtp2	224055

Supplementary Table X. Genes down-regulated in MES adjacent to OE E14.5 vs. OPL 11.5. The gene symbols, gene names, Entrez gene ID, fold-change and significance are reported. DEGs are ranked according to the "fold-change". The non-annotated probes have been eliminated.

log2 FC	P value	Gene Name	Symbol	Entrez ID
-1,54617	0,0004	nuclear receptor subfamily 2, group F, member 1	Nr2f1	13865
-1,56662	0,0003	carbonic anhydrase 2	Car2	12349
-1,57425	0,0003	immunoglobulin superfamily containing leucine-rich repeat 2	Islr2	320563
-1,57701	0,0003	transforming growth factor, beta induced	Tgfbi	21810
-1,5912	0,0003	Friend leukemia integration 1	Fli1	14247
-1,60231	0,0002	stimulated by retinoic acid gene 6	Stra6	20897
-1,61753	0,0002	insulin-like growth factor 2 mRNA binding protein 1	Igf2bp1	140486
-1,63469	0,0002	hydroxy-delta-5-steroid dehydrogenase, 3 beta- and steroid delta-isomerase 2 /// hydroxy-delta-5-steroid dehydrogenase, 3 beta- and steroid delta-isomerase 6 /// hydroxy-delta-5-steroid dehydrogenase, 3 beta- and steroid delta-isomerase 3	Hsd3b2 /// Hsd3b6 /// Hsd3b3	15493 /// 15497 /// 15494
-1,66015	0,0002	RIKEN cDNA 2610318N02 gene	2610318N02Rik	70458
-1,676	0,0002	fibronectin leucine rich transmembrane protein 2	Flrt2	399558
-1,68769	0,0002	crystallin, mu	Crym	12971
-1,75249	0,0001	SH3-domain GRB2-like 3	Sh3gl3	20408
-1,77707	0,0001	homeobox, msh-like 2	Msx2	17702
-1,79456	0,0001	sal-like 4 (Drosophila)	Sall4	99377
-1,79581	0,0001	dual specificity phosphatase 9	Dusp9	75590
-1,79693	0,0001	forkhead box F2	Foxf2	14238
-1,85834	0,0001	cerebellin 1 precursor protein	Cbln1	12404
-1,87019	0,0001	olfactory receptor 20	Olfr20	258925
-1,87531	0,0001	ubiquitin-like 4B	Ubl4b	67591
-1,90497	0	R-spondin 2 homolog (Xenopus laevis)	Rspo2	239405
-1,90981	0	glycophorin A	Gypa	14934
-1,91594	0	Cbp/p300-interacting transactivator with Glu/Asp-rich carboxy-terminal domain 1	Cited1	12705
-1,92847	0	aristaless-like homeobox 3	Alx3	11694
-2,00043	0	polycystic kidney and hepatic disease 1-like 1	Pkhd1l1	192190
-2,00141	0	limb expression 1 homolog (chicken)	Lix1	66643
-2,02404	0	forkhead box C2	Foxc2	14234
-2,04282	0	RIKEN cDNA 1200009O22 gene	1200009O22Rik	66873
-2,07173	0	gene regulated by estrogen in breast cancer protein	Greb1	268527
-2,11357	0	Kell blood group	Kel	23925
-2,1552	0	5-hydroxytryptamine (serotonin) receptor 3B	Htr3b	57014
-2,21844	0	solute carrier family 4 (anion exchanger), member 1	Slc4a1	20533
-2,38722	0	Rhesus blood group-associated A glycoprotein	Rhag	19743
-2,41099	0	hemogen	Hemgn	93966
-2,42366	0	sal-like 4 (Drosophila)	Sall4	99377
-2,47171	0	carbonic anhydrase 14	Car14	23831
-2,63939	0	RIKEN cDNA 5031408O05 gene /// gene model 784, (NCBI)	5031408O05Rik /// Gm784	331491 /// 333564
-2,75666	0	hemoglobin X, alpha-like embryonic chain in Hba complex	Hba-x	15126
-2,9805	0	T-box 22	Tbx22	245572
-3,66812	0	forkhead box F1a	Foxf1a	15227
-5,35247	0	hemoglobin Z, beta-like embryonic chain /// predicted gene, EG436003	Hbb-bh1 /// EG436003	15132 /// 436003

upplementary Table XI. Genes up-regulated in MES adjacent to VNO E14.5 vs. OPL 11.5. The gene symbols, gene names, Entrez gene ID, fold-change and significance are reported. DEGs are ranked according to the "fold-change". The non-annotated probes have been eliminated.

log2 FC	P value	Gene Name	Symbol	Entrez ID
6,965784	0	asporin	Aspn	66695
6,097888	0	UDP glucuronosyltransferase 2 family, polypeptide A1 /// UDP glucuronosyltransferase 2 family, polypeptide A2	Ugt2a1 /// Ugt2a2	94215 /// 552899
5,6878	0	palate, lung, and nasal epithelium associated	Plunc	18843
5,321928	0	cytochrome P450, family 2, subfamily g, polypeptide 1	Cyp2g1	13108
4,717857	0	leukocyte cell derived chemotaxin 1	Lect1	16840
4,536168	0	regenerating islet-derived 3 gamma	Reg3g	19695
4,512925	0	matrix Gla protein	Mgp	17313
4,356975	0	sulfotransferase family 1E, member 1	Sult1e1	20860
4,237864	0	follistatin-like 5	Fstl5	213262
4,132894	0	matrilin 1, cartilage matrix protein	Matn1	17180
4,016249	0	contactin 4	Cntn4	269784
3,974829	0	thrombospondin 2	Thbs2	21826
3,895395	0	sodium channel, voltage-gated, type IX, alpha	Scn9a	20274
3,867752	0	aggrecan	Acan	11595
3,809954	0	aldehyde oxidase 3-like 1	Aox3l1	213043
3,789861	0	ets homologous factor	Ehf	13661
3,719756	0	E74-like factor 5	Elf5	13711
3,717857	0	serine (or cysteine) peptidase inhibitor, clade B (ovalbumin), member 11	Serpinb11	66957
3,647467	0	cytochrome P450, family 2, subfamily f, polypeptide 2	Cyp2f2	13107
3,634867	0	carbonyl reductase 2	Cbr2	12409
3,53952	0	ABI gene family, member 3 (NESH) binding protein	Abi3bp	320712
3,431287	0	ermin, ERM-like protein	Ermn	77767
3,417348	0	solute carrier family 27 (fatty acid transporter), member 2	Slc27a2	26458
3,406598	0	integrin, beta-like 1	Itgbl1	223272
3,395929	0	tripartite motif-containing 66	Trim66	330627
3,394411	0	serine/threonine kinase 32A	Stk32a	269019
3,388355	0	integrin binding sialoprotein	Ibsp	15891
3,373327	0	decorin	Dcn	13179
3,36736	0	aquaporin 1	Aqp1	11826
3,365871	0	nescient helix loop helix 1	Nhlh1	18071
3,365871	0	collagen, type XIV, alpha 1	Col14a1	12818
3,257045	0	zinc finger, CCHC domain containing 5	Zcchc5	213436
3,184425	0	sulfotransferase family, cytosolic, 1C, member 1	Sult1c1	20888
3,155855	0	glutamyl aminopeptidase	Enpep	13809
3,148161	0	annexin A1	Anxa1	16952
3,090496	0	sulfotransferase family 1D, member 1	Sult1d1	53315
3,079478	0	periostin, osteoblast specific factor	Postn	50706
3,069755	0	G protein-coupled receptor 126	Gpr126	215798
3,0613	0	V-set and transmembrane domain containing 2A	Vstm2a	211739
3,030325	0	osteoglycin	Ogn	18295
2,98965	0	RIKEN cDNA 4930431L04 gene	4930431L04Rik	270049
2,953439	0	early B-cell factor 2	Ebf2	13592
2,937878	0	CD55 antigen	Cd55	13136
2,925769	0	elastin	Eln	13717
2,912673	0	matrilin 4	Matn4	17183
2,909418	0	testis expressed gene 15	Tex15	104271

2,863546	0	myelin transcription factor 1-like	Myt1l	17933
2,842698	0	regulator of G-protein signaling 5	Rgs5	19737
2,824188	0	neurexin I	Nrxn1	18189
2,809954	0	actin, alpha 2, smooth muscle, aorta	Acta2	11475
2,782893	0	activating transcription factor 5	Atf5	107503
2,768076	0	olfactory receptor 430	Olfr430	258713
2,756331	0	scinderin	Scin	20259
2,693388	0	epoxide hydrolase 1, microsomal	Ephx1	13849
2,693388	0	odd-skipped related 2 (Drosophila)	Osr2	107587
2,6878	0	keratocan	Kera	16545
2,681307	0	aquaporin 3	Aqp3	11828
2,67761	0	creatine kinase, mitochondrial 1, ubiquitous	Ckmt1	12716
2,673923	0	sparc/osteonectin, cwcv and kazal-like domains proteoglycan 1	Spock1	20745
2,652901	0	ATPase, Na+/K+ transporting, beta 1 polypeptide	Atp1b1	11931
2,652901	0	ELAV (embryonic lethal, abnormal vision, Drosophila)-like 3 (Hu antigen C)	Elavl3	15571
2,646564	0	tandem C2 domains, nuclear	Tc2n	74413
2,639355	0	olfactory receptor 972	Olfr972	258603
2,635764	0	SV2 related protein homolog (rat)-like	Svopl	320590
2,614403	0	hyaluronan and proteoglycan link protein 1	Hapln1	12950
2,586406	0	osteomodulin	Omd	27047
2,576906	0	coiled-coil domain containing 3	Ccdc3	74186
2,573467	0	receptor transporter protein 1	Rtp1	239766
2,572608	0	collagen, type VIII, alpha 1	Col8a1	12837
2,564905	0	mucin 4	Muc4	140474
2,564905	0	acyl-CoA synthetase medium-chain family member 4	Acsm4	233801
2,558942	0	piccolo (presynaptic cytomatrix protein)	Pclo	26875
2,527824	0	CD36 antigen	Cd36	12491
2,526161	0	gene model 606, (NCBI)	Gm606	239789
2,508814	0	thrombospondin, type I, domain containing 7B	Thsd7b	210417
2,506353	0	leucine-rich repeat LGI family, member 2	Lgi2	246316
2,490051	0	S100 calcium binding protein A14	S100a14	66166
2,477141	0	flavin containing monooxygenase 1	Fmo1	14261
2,475535	0	interferon inducible GTPase 1 /// interferon-inducible GTPase-like /// RIKEN cDNA F830016B08 gene /// predicted gene, EG240327	Iigp1 /// LOC435565 /// F830016B08Rik /// EG240327	60440 /// 435565 /// 240328 /// 240327
2,453241	0	Rho GDP dissociation inhibitor (GDI) gamma	Arhgdig	14570
2,442222	0	3'-phosphoadenosine 5'-phosphosulfate synthase 2	Papss2	23972
2,428178	0	mal, T-cell differentiation protein 2	Mal2	105853
2,4243	0	connective tissue growth factor	Ctgf	14219
2,421979	0	B-cell leukemia/lymphoma 6	Bcl6	12053
2,41889	0	RIKEN cDNA 4932418E24 gene	4932418E24Rik	329366
2,39973	0	keratin 17	Krt17	16667
2,397448	0	flavin containing monooxygenase 2	Fmo2	55990
2,383078	0	collagen, type XXIV, alpha 1	Col24a1	71355
2,374823	0	tubulin tyrosine ligase-like family, member 6	Ttll6	237930
2,36736	0,0001	paraoxonase 1	Pon1	18979
2,365128	0,0001	aldolase C, fructose-bisphosphate	Aldoc	11676
2,354759	0,0001	cytoplasmic polyadenylation element binding protein 1	Cpeb1	12877
2,344465	0,0001	stathmin-like 2	Stmn2	20257
2,323372	0,0001	leucine rich repeat and Ig domain containing 2	Lingo2	242384
2,321207	0,0001	Ca2+-dependent secretion activator	Cadps	27062
2,314733	0,0001	olfactory receptor 1006	Olfr1006	258563

2,312581	0,0001	a disintegrin and metallopeptidase domain 28	Adam28	13522
2,312581	0,0001	microtubule associated monoxygenase, calponin and LIM domain containing 2	Mical2	320878
2,310432	0,0001	elongation factor RNA polymerase II-like 3	Ell3	269344
2,298318	0,0001	collagen, type XI, alpha 1	Col11a1	12814
2,297608	0,0001	epiphycan	Epyc	13516
2,278584	0,0001	serine (or cysteine) peptidase inhibitor, clade B, member 7	Serpinb7	116872
2,277184	0,0001	sema domain, immunoglobulin domain (Ig), short basic domain, secreted, (semaphorin) 3C	Sema3c	20348
2,268122	0,0001	N-myc downstream regulated gene 1	Ndrg1	17988
2,266732	0,0001	Ca2+-dependent activator protein for secretion 2	Cadps2	320405
2,263265	0,0001	alcohol dehydrogenase 1 (class I)	Adh1	11522
2,258425	0,0001	calsyntenin 2	Clstn2	64085
2,254978	0,0001	forkhead box A1	Foxa1	15375
2,254289	0,0001	indolethylamine N-methyltransferase	Inmt	21743
2,237864	0,0001	leucine-rich repeat LGI family, member 1	Lgi1	56839
2,227016	0,0001	matrilin 3	Matn3	17182
2,225666	0,0001	chloride intracellular channel 6	Clic6	209195
2,21424	0,0001	runt related transcription factor 1	Runx1	12394
2,182459	0,0001	mitogen-activated protein kinase 10	Mapk10	26414
2,177882	0,0001	aryl-hydrocarbon receptor	Ahr	11622
2,174621	0,0001	gamma-aminobutyric acid (GABA-A) receptor, subunit alpha 1	Gabra1	14394
2,171368	0,0001	olfactory receptor 498	Olfr498	258304
2,159718	0,0001	STEAP family member 4	Steap4	117167
2,155213	0,0001	thiosulfate sulfurtransferase, mitochondrial	Tst	22117
2,15457	0,0001	cyclic nucleotide gated channel alpha 2	Cnga2	12789
2,152003	0,0001	sema domain, immunoglobulin domain (Ig), short basic domain, secreted, (semaphorin) 3D	Sema3d	108151
2,145605	0,0001	tumor protein D52	Tpd52	21985
2,143054	0,0001	tescalcin	Tesc	57816
2,136696	0,0001	Fas apoptotic inhibitory molecule 2	Faim2	72393
2,128472	0,0001	solute carrier family 24 (sodium/potassium/calcium exchanger), member 2	Slc24a2	76376
2,124063	0,0001	fatty acid binding protein 7, brain	Fabp7	12140
2,123434	0,0001	immunoglobulin-like domain containing receptor 1	Ildr1	106347
2,122177	0,0001	matrix metallopeptidase 9	Mmp9	17395
2,11904	0,0001	growth differentiation factor 10	Gdf10	14560
2,11904	0,0001	cDNA sequence BC048546	BC048546	232400
2,11466	0,0001	glutamate receptor, ionotropic, kainate 1	Grik1	14805
2,107803	0,0001	N-myc downstream regulated gene 2	Ndrg2	29811
2,098506	0,0001	RIKEN cDNA E030002O03 gene	E030002O03Rik	244180
2,075824	0,0001	keratin 18	Krt18	16668
2,073393	0,0001	collagen, type XII, alpha 1	Col12a1	12816
2,072786	0,0001	potassium large conductance calcium-activated channel, subfamily M, alpha member 1	Kcnma1	16531
2,070967	0,0001	trinucleotide repeat containing 4	Tnrc4	78784
2,069755	0,0001	myosin VC	Myo5c	208943
2,067334	0,0001	potassium inwardly-rectifying channel, subfamily J, member 3	Kcnj3	16519
2,06673	0,0001	nuclear factor I/X	Nfix	18032
2,066125	0,0001	RAS-like, estrogen-regulated, growth-inhibitor	Rerg	232441
2,057692	0,0001	RIKEN cDNA A930006J02 gene	A930006J02Rik	77790
2,0511	0,0002	RIKEN cDNA D930030D11 gene	D930030D11Rik	320874
2,048711	0,0001	secreted frizzled-related protein 4	Sfrp4	20379

2,045134	0,0001	transient receptor potential cation channel, subfamily V, member 4	Trpv4	63873
2,035638	0,0002	thrombospondin, type I, domain containing 4	Thsd4	207596
2,033274	0,0002	keratin 15	Krt15	16665
2,031504	0,0002	RAB4B, member RAS oncogene family /// melanoma inhibitory activity 1	Rab4b /// Mia1	19342 /// 12587
2,029146	0,0002	RAB25, member RAS oncogene family	Rab25	53868
2,025618	0,0002	A kinase (PRKA) anchor protein 6	Akap6	238161
2,018001	0,0002	regulator of G-protein signaling 5	Rgs5	19737
2,000577	0,0002	neuropilin 1	Nrp1	18186
1,999423	0,0002	sema domain, immunoglobulin domain (Ig), short basic domain, secreted, (semaphorin) 3E	Sema3e	20349
1,997117	0,0002	orthodenticle homolog 2 (Drosophila)	Otx2	18424
1,977668	0,0002	grainyhead-like 1 (Drosophila)	Grhl1	195733
1,971431	0,0002	ELAV (embryonic lethal, abnormal vision, Drosophila)-like 4 (Hu antigen D)	Elavl4	15572
1,968605	0,0002	Purkinje cell protein 4	Pcp4	18546
1,966912	0,0002	olfactory receptor 68	Olfr68	18369
1,964658	0,0002	S100 protein, beta polypeptide, neural	S100b	20203
1,963532	0,0002	coagulation factor II (thrombin) receptor-like 1	F2rl1	14063
1,942862	0,0002	grancalcin	Gca	227960
1,941199	0,0002	reticulon 1	Rtn1	104001
1,938984	0,0002	RIKEN cDNA D930020B18 gene	D930020B18Rik	216393
1,935669	0,0002	glutamate receptor, ionotropic, delta 2	Grid2	14804
1,931811	0,0002	claudin 9	Cldn9	56863
1,926317	0,0002	keratin 19	Krt19	16669
1,924673	0,0002	fibronectin leucine rich transmembrane protein 1	Flrt1	396184
1,924673	0,0003	calmodulin-like 4	Calml4	75600
1,923031	0,0002	keratin 23	Krt23	94179
1,921937	0,0002	beta-1,4-N-acetyl-galactosaminyl transferase 3	B4galnt3	330406
1,91648	0,0002	nephroblastoma overexpressed gene	Nov	18133
1,911587	0,0002	solute carrier family 16 (monocarboxylic acid transporters), member 4	Slc16a4	229699
1,909418	0,0002	synaptotagmin I	Syt1	20979
1,907793	0,0003	mesothelin-like	Mslnl	328783
1,905629	0,0003	olfactory receptor 1176	Olfr1176	258767
1,904548	0,0003	chondroadherin	Chad	12643
1,904008	0,0003	small proline-rich protein 1A	Sprr1a	20753
1,903468	0,0002	DnaJ (Hsp40) homolog, subfamily C, member 6	Dnajc6	72685
1,90185	0,0003	tetraspanin 8	Tspan8	216350
1,901311	0,0003	stathmin-like 3	Stmn3	20262
1,899157	0,0003	guanylate cyclase 1, soluble, alpha 3	Gucy1a3	60596
1,897543	0,0002	nucleolar protein 4	Nol4	319211
1,897006	0,0003	ATPase, H+ transporting, lysosomal V1 subunit B1	Atp6v1b1	110935
1,894858	0,0003	odd Oz/ten-m homolog 1 (Drosophila)	Odz1	23963
1,891108	0,0003	secreted frizzled-related protein 2	Sfrp2	20319
1,890573	0,0003	olfactory receptor 344	Olfr344	258621
1,884168	0,0003	fibroblast growth factor 7	Fgf7	14178
1,878321	0,0003	potassium voltage-gated channel, Isk-related subfamily, gene 4	Kcne4	57814
1,878321	0,0003	internexin neuronal intermediate filament protein, alpha	Ina	226180
1,867226	0,0003	carboxylesterase 3	Ces3	104158
1,861972	0,0003	nuclear receptor binding protein 2	Nrbp2	223649
1,854127	0,0003	cyclin-dependent kinase inhibitor 1A (P21)	Cdkn1a	12575
1,847362	0,0003	CD9 antigen	Cd9	12527

1,838564	0,0004	C1q and tumor necrosis factor related protein 3	C1qtnf3	81799
1,835986	0,0003	family with sequence similarity 107, member A	Fam107a	268709
1,833413	0,0003	potassium voltage-gated channel, Shal-related family, member 2	Kcnd2	16508
1,832385	0,0003	claudin 3	Cldn3	12739
1,831871	0,0003	collagen, type IX, alpha 3	Col9a3	12841
1,829928	0,0016	LIM homeobox protein 2	Lhx2	16870
1,827256	0,0003	receptor transporter protein 2	Rtp2	224055
1,821636	0,0004	neural cell adhesion molecule 2	Ncam2	17968
1,812486	0,0004	bone morphogenetic protein 5	Bmp5	12160
1,808943	0,0004	neurexophilin 1	Nxph1	18231
1,808943	0,0004	dynein light chain roadblock-type 2	Dynlrb2	75465
1,803393	0,0004	desmocollin 2	Dsc2	13506
1,802889	0,0004	tetraspanin 1	Tspan1	66805
1,79636	0,0004	claudin 7	Cldn7	53624
1,795358	0,0004	suppressor of cytokine signaling 2	Socs2	216233
1,793357	0,0004	transglutaminase 2, C polypeptide	Tgm2	21817
1,79371	0,0146	doublecortin	Dcx	13193
1,788863	0,0004	RNA binding motif protein 47	Rbm47	245945
1,782893	0,0004	mitogen-activated protein kinase 10	Mapk10	26414
1,782397	0,0004	RIKEN cDNA 9130221D24 gene	9130221D24Rik	77669
1,781901	0,0004	caveolin 1, caveolae protein	Cav1	12389
1,77596	0,0004	polymerase I and transcript release factor	Ptrf	19285
1,772013	0,0004	chondrolectin	Chodl	246048
1,762192	0,0004	F-box protein 32	Fbxo32	67731
1,755844	0,0004	otoraplin	Otor	57329
1,752923	0,0005	epithelial cell adhesion molecule	Epcam	17075
1,739854	0,0005	olfactory receptor 24	Olfr24	18322
1,73889	0,0005	canopy 1 homolog (zebrafish)	Cnpy1	269637
1,728813	0,0005	RAB6B, member RAS oncogene family	Rab6b	270192
1,724516	0,0006	ATPase, H+ transporting, lysosomal V0 subunit A4 /// RIKEN cDNA D630045J12 gene	Atp6v0a4 /// D630045J12Rik	140494 /// 330286
1,717382	0,0006	olfactory receptor 1264	Olfr1264	258206
1,713119	0,0006	FGGY carbohydrate kinase domain containing	Fggy	75578
1,711701	0,0006	very low density lipoprotein receptor	Vldlr	22359
1,70934	0,0005	expressed sequence AI646023	AI646023	192734
1,707925	0,0006	pannexin 3	Panx3	208098
1,70275	0,0006	transient receptor potential cation channel, subfamily M, member 5	Trpm5	56843
1,698998	0,0006	leucine rich repeat containing G protein coupled receptor 5	Lgr5	14160
1,696658	0,0006	cAMP responsive element binding protein 3-like 1	Creb3l1	26427
1,697324	0,0011	peripherin	Prph	19132
1,69619	0,0006	chondroitin sulfate N-acetylgalactosaminyltransferase 1	Csgalnact1	234356
1,691057	0,0006	S100 calcium binding protein A1	S100a1	20193
1,687335	0,0006	adenylate cyclase 2	Adcy2	210044
1,680845	0,0006	transient receptor potential cation channel, subfamily M, member 3	Trpm3	226025
1,677149	0,0006	neurotrophic tyrosine kinase, receptor, type 2	Ntrk2	18212
1,676226	0,0006	endothelin receptor type B	Ednrb	13618
1,673003	0,0007	cytochrome P450, family 2, subfamily a, polypeptide 4 /// cytochrome P450, family 2, subfamily a, polypeptide 5	Cyp2a4 /// Cyp2a5	13086 /// 13087
1,671623	0,0007	monoamine oxidase B	Maob	109731
1,669327	0,0006	calcium/calmodulin-dependent protein kinase II inhibitor 1	Camk2n1	66259
1,669327	0,0006	sodium channel, voltage-gated, type III, beta	Scn3b	235281
1,663831	0,0007	Eph receptor A6	Epha6	13840

1,659723	0,0007	C-type lectin domain family 2, member d /// C-type lectin domain family 2, member i /// killer cell lectin-like receptor subfamily B member 1, pseudogene 1	Clec2d /// Clec2i /// Klrb1-ps1	93694 /// 93675 /// 724020
1,655626	0,0007	potassium voltage-gated channel, subfamily H (eag-related), member 7	Kcnh7	170738
1,650635	0,0008	ATPase, H+ transporting, lysosomal V1 subunit C2	Atp6v1c2	68775
1,641154	0,0007	amyloid beta (A4) precursor protein binding, family A, member 1	Apba1	319924
1,640132	0,0177	neurogenin 1	Neurog1	18014
1,636212	0,0007	leucine rich repeat transmembrane neuronal 1	Lrrtm1	74342
1,634867	0,0008	synaptophysin	Syp	20977
1,634419	0,0008	CDP-diacylglycerol synthase 1	Cds1	74596
1,633524	0,0008	protein arginine N-methyltransferase 8	Prmt8	381813
1,632629	0,0008	troponin C, cardiac/slow skeletal	Tnnc1	21924
1,632182	0,0008	Williams-Beuren syndrome chromosome region 17 homolog (human)	Wbscr17	212996
1,631288	0,0008	mitogen-activated protein kinase kinase kinase 5	Map3k5	26408
1,627716	0,0008	fibroblast growth factor 12	Fgf12	14167
1,627271	0,0008	clusterin	Clu	12759
1,626825	0,0011	selenoprotein K	Selk	80795
1,625934	0,0008	teashirt zinc finger family member 2	Tshz2	228911
1,62371	0,0008	transmembrane serine protease 6	Tmprss6	71753
1,621932	0,0009	progestin and adipoQ receptor family member IX	Paqr9	75552
1,617499	0,0009	RIKEN cDNA 4930506M07 gene	4930506M07Rik	71653
1,611755	0,0009	regulator of G-protein signaling 2	Rgs2	19735
1,607353	0,0009	met proto-oncogene	Met	17295
1,602964	0,0009	guanine nucleotide binding protein, alpha O	Gnao1	14681
1,595969	0,0009	adaptor-related protein complex 3, beta 2 subunit	Ap3b2	11775
1,594661	0,0009	RNA binding motif protein 35A	Rbm35a	207920
1,592049	0,0009	phosphatase and actin regulator 1	Phactr1	218194
1,589442	0,001	RNA binding motif protein 11	Rbm11	224344
1,58814	0,001	basic helix-loop-helix family, member e40	Bhlhe40	20893
1,58814	0,0009	serine protease inhibitor, Kunitz type 2	Spint2	20733
1,586839	0,0009	mitogen-activated protein kinase 13	Mapk13	26415
1,585107	0,001	anoctamin 2	Ano2	243634
1,576476	0,001	epithelial membrane protein 3	Emp3	13732
1,576476	0,001	zinc finger, DHHC domain containing 2	Zdhhc2	70546
1,571322	0,001	beaded filament structural protein 2, phakinin	Bfsp2	107993
1,570893	0,001	sodium leak channel, non-selective	Nalcn	338370
1,570893	0,0011	cadherin-like 26	Cdh26	381409
1,568324	0,001	sulfotransferase family 4A, member 1	Sult4a1	29859
1,567041	0,0013	chemokine (C-X-C motif) ligand 5	Cxcl5	20311
1,566186	0,0011	RIKEN cDNA 1700003M02 gene	1700003M02Rik	69329
1,565332	0,001	Fc receptor, IgG, alpha chain transporter	Fcgrt	14132
1,564051	0,001	guanine nucleotide binding protein (G protein), gamma 8	Gng8	14709
1,55385	0,0012	chemokine (C-X-C motif) receptor 4	Cxcr4	12767
1,551733	0,0013	trace amine-associated receptor 7D /// trace amine-associated receptor 7E /// trace amine-associated receptor 7A /// trace amine-associated receptor 7B /// trace amine-associated receptor 7F	Taar7d /// Taar7e /// Taar7a /// Taar7b /// Taar7f	435206 /// 276742 /// 215856 /// 209517 /// 435207
1,547088	0,0011	gamma-glutamyltransferase 7	Ggt7	207182
1,54414	0,0012	tubulin, beta 3 /// melanocortin 1 receptor	Tubb3 /// Mc1r	22152 /// 17199
1,543299	0,0011	keratin 14	Krt14	16664

1,5391	0,0012	dihydropyrimidinase-like 3	Dpysl3	22240
1,535332	0,0012	grainyhead-like 2 (Drosophila)	Grhl2	252973
1,534914	0,0012	cystic fibrosis transmembrane conductance regulator homolog	Cftr	12638
1,534078	0,0012	unc-13 homolog C (C. elegans)	Unc13c	208898
1,52367	0,0012	reprimo, TP53 dependent G2 arrest mediator candidate	Rprm	67874

Supplementary Table XII. Genes down-regulated in MES adjacent to VNO E14.5 vs. OPL 11.5. The gene symbols, gene names, Entrez gene ID, fold-change and significance are reported. DEGs are ranked according to the "fold-change". The non-annotated probes have been eliminated.

log2 FC	P-value	Gene Name	Symbol	Entrez ID
-1,63124	0,0004	Cbp/p300-interacting transactivator with Glu/Asp-rich carboxy-terminal domain 1	Cited1	12705
-1,63608	0,0004	hydroxy-delta-5-steroid dehydrogenase, 3 beta- and steroid delta-isomerase 2 /// hydroxy-delta-5-steroid dehydrogenase, 3 beta- and steroid delta-isomerase 6 /// hydroxy-delta-5-steroid dehydrogenase, 3 beta- and steroid delta-isomerase 3	Hsd3b2 /// Hsd3b6 /// Hsd3b3	15493 /// 15497 /// 15494
-1,6397	0,0004	UDP-GlcNAc:betaGal beta-1,3-N-acetylglucosaminyltransferase 5	B3gnt5	108105
-1,64247	0,0003	RIKEN cDNA 2610318N02 gene	2610318N02Rik	70458
-1,67383	0,0003	histone cluster 1, H3i /// histone cluster 1, H3g /// histone cluster 1, H3d /// histone cluster 1, H3e /// histone cluster 1, H3b /// histone cluster 1, H3h	Hist1h3i /// Hist1h3g /// Hist1h3d /// Hist1h3e /// Hist1h3b /// Hist1h3h	319153 /// 97908 /// 319149 /// 319151 /// 319150 /// 319152
-1,68055	0,0003	transcription factor AP-2, alpha	Tcfap2a	21418
-1,68244	0,0003	adenomatosis polyposis coli down-regulated 1	Apcdd1	494504
-1,691	0,0003	Friend leukemia integration 1	Fli1	14247
-1,72888	0,0002	GLI pathogenesis-related 2	Glipr2	384009
-1,7563	0,0001	sal-like 4 (Drosophila)	Sall4	99377
-1,8166	0,0001	branched chain aminotransferase 1, cytosolic	Bcat1	12035
-1,81914	0,0001	homeobox, msh-like 1	Msx1	17701
-1,85965	0,0001	zinc finger protein of the cerebellum 1	Zic1	22771
-1,87704	0,0001	polycystic kidney and hepatic disease 1-like 1	Pkhd1l1	192190
-1,90708	0,0001	gene regulated by estrogen in breast cancer protein	Greb1	268527
-1,92456	0,0001	cerebellin 1 precursor protein	Cbln1	12404
-1,94035	0,0001	stearoyl-Coenzyme A desaturase 1	Scd1	20249
-1,95416	0,0001	sal-like 4 (Drosophila)	Sall4	99377
-1,97229	0,0001	homeobox, msh-like 2	Msx2	17702
-2,00364	0	glycophorin A	Gypa	14934
-2,00985	0	RIKEN cDNA 1200009O22 gene	1200009O22Rik	66873
-2,12029	0	paired box gene 7	Pax7	18509
-2,20286	0	insulin-like growth factor 2 mRNA binding protein 1	Igf2bp1	140486
-2,21288	0	solute carrier family 4 (anion exchanger), member 1	Slc4a1	20533
-2,24394	0	Kell blood group	Kel	23925
-2,29328	0	POU domain, class 3, transcription factor 4	Pou3f4	18994
-2,2996	0	hemogen	Hemgn	93966
-2,31947	0	lymphoid enhancer binding factor 1	Lef1	16842
-2,34792	0	carbonic anhydrase 14	Car14	23831
-2,4393	0	Rhesus blood group-associated A glycoprotein	Rhag	19743
-2,84874	0	RIKEN cDNA 5031408O05 gene /// gene model 784, (NCBI)	5031408O05Rik /// Gm784	331491 /// 333564
-2,8826	0	limb expression 1 homolog (chicken)	Lix1	66643
-2,93804	0	hemoglobin X, alpha-like embryonic chain in Hba complex	Hba-x	15126
-4,90555	0	hemoglobin Z, beta-like embryonic chain /// predicted gene, EG436003	Hbb-bh1 /// EG436003	15132 /// 436003

upplementary Table XIII. Genes differentially expressed in the OE and VNO of Dlx5-/- vs. wild-type embryos, age E12.5. The gene symbols, gene names, Entrez gene ID, fold-change and the presence of predicted Dlx5 binding sites are reported. DEGs are ranked according to the "fold-change". The non-annotated probes have been eliminated. The up-regulated DEGs are on the top (light blue), the down-regulated DEGs at the bottom (light pink).

Gene Name	Symbol	log2 FC	Dlx5 sites
acyl-CoA synthetase medium-chain family member 4	Acsm4	-2,478609468	
cytochrome P450, family 2, subfamily g, polypeptide 1	Cyp2g1	-2,453938935	
sulfotransferase family 1E, member 1	Sult1e1	-2,156461339	
trace amine-associated receptor 7A	Taar7a /// Taar7b /// Taar7d /// Taar7e /// Taar7f	-2,140484198	
glycoprotein hormones, alpha subunit	Cga	-2,059355278	+
fatty acid binding protein 7, brain	Fabp7	-1,962030707	
Purkinje cell protein 4	Pcp4	-1,89782126	+
predicted gene 606	Gm606	-1,855072777	+
solute carrier family 25, member 35	Slc25a35	-1,827371923	
N-terminal EF-hand calcium binding protein 1	Necab1	-1,7193587	
cadherin-like 26	Cdh26	-1,70606634	+
RIKEN cDNA A930006J02 gene	A930006J02Rik	-1,664528351	
receptor transporter protein 1	Rtp1	-1,654802126	
monoamine oxidase B	Maob	-1,615604626	
solute carrier family 27 (fatty acid transporter), member 2	Slc27a2	-1,605020153	+
RIKEN cDNA E330013P04 gene	E330013P04Rik	-1,587268968	
RIKEN cDNA C130071C03 gene	C130071C03Rik	-1,581977846	
A kinase (PRKA) anchor protein 6	Akap6	-1,518635847	+
anterior gradient homolog 3 (Xenopus laevis)	Agr3	-1,518081865	+
ermin, ERM-like protein	Ermn	-1,503348735	
cholinergic receptor, nicotinic, alpha polypeptide 3	Chrna3	-1,489388793	+
gonadotropin releasing hormone 1	Gnrh1	-1,481247268	
replication protein A3	Rpa3	-1,44826666	
leucine rich repeat and Ig domain containing 2	Lingo2	-1,399553938	+
UDP glucuronosyltransferase 2 family, polypeptide B34 ///	Ugt2b34 /// Ugt2a1 /// Ugt2a2	-1,399116393	
visinin-like 1	Vsnl1	-1,386093558	+
predicted gene 9853	Gm9853	-1,379177329	
tubulin polymerization-promoting protein family member 3	Tppp3	-1,378456134	
coagulation factor II (thrombin) receptor-like 1	F2rl1	-1,375790133	
nucleolar protein 4	Nol4	-1,36305891	+
leucine-rich repeat LGI family, member 1	Lgi1	-1,355411006	+
regulator of G-protein signaling 5	Rgs5	-1,351458723	

synaptosomal-associated protein 25	Snap25	-1,348175753	+
ST8 alpha-N-acetyl-neuraminide alpha-2,8-sialyltransferase 6	St8sia6	-1,347212086	+
synaptic vesicle glycoprotein 2 b	Sv2b	-1,331647897	+
SV2 related protein homolog (rat)-like	Svopl	-1,330214177	
sulfotransferase family 1D, member 1	Sult1d1	-1,307894776	
ATP-binding cassette, sub-family D (ALD), member 2	Abcd2	-1,307836502	+
C-type lectin domain family 12, member a	Clec12a	-1,231002239	
tachykinin 1	Tac1	-1,230264508	+
D4, zinc and double PHD fingers, family 3	Dpf3	-1,217292766	+
ATPase, H+ transporting, lysosomal V1 subunit G3	Atp6v1g3	-1,216485907	+
calmegin	Clgn	-1,214995135	
dual adaptor for phosphotyrosine and 3-phosphoinositides 1	Dapp1	-1,209453366	
polymerase (RNA) II (DNA directed) polypeptide H	Polr2h	-1,203890227	
keratin 23	Krt23	-1,200316016	+
ganglioside-induced differentiation-associated-protein 1	Gdap1	-1,193582525	+
unc-13 homolog C (C. elegans)	Unc13c	-1,175684271	+
Purkinje cell protein 4-like 1	Pcp4l1	-1,172231469	+
bone morphogenetic protein receptor, type 1B	Bmpr1b	-1,170309669	+
secretogranin III	Scg3	-1,168577857	
ARP3 actin-related protein 3 homolog B (yeast)	Actr3b	-1,160855665	
RIKEN cDNA 9330120H11 gene	9330120H11Rik	-1,158918642	
canopy 1 homolog (zebrafish)	Cnpy1	-1,157431827	
RIKEN cDNA 5730408A14 gene ///	5730408A14Rik /// 6330418B08Rik /// Rgs7	-1,147046143	
follistatin-like 5	Fstl5	-1,142609487	+
cyclic nucleotide gated channel alpha 2	Cnga2	-1,129348967	+
stathmin-like 3	Stmn3	-1,127237062	
claudin 1	Cldn1	-1,126047755	+
guanine nucleotide binding protein (G protein), gamma 3	Gng3	-1,123931008	
ATP-binding cassette, sub-family A (ABC1), member 13	Abca13	-1,119887336	+
UDP galactosyltransferase 8A	Ugt8a	-1,113967146	+
RIKEN cDNA 1700028P14 gene	1700028P14Rik	-1,112966965	+
sodium channel, voltage-gated, type III, beta	Scn3b	-1,112900262	
vesicle amine transport protein 1 homolog-like (T. californica)	Vat1l	-1,10701825	+
RIKEN cDNA 1700003M02 gene	1700003M02Rik	-1,10514166	+
mesoderm induction early response 1 homolog (Xenopus laevis	Mier1	-1,10191896	
brain expressed X-linked 2 /// brain expressed gene 4 ///	Bex2 /// Bex4 /// Bex1	-1,101650076	
synaptotagmin I	Syt1	-1,100977648	+
potassium voltage-gated channel, Shal-related family, member 2	Kcnd2	-1,100237614	+

paraoxonase 1	Pon1	-1,094573898	+
solute carrier family 14 (urea transporter), member 1	Slc14a1	-1,093154488	
ELMO domain containing 1	Elmod1	-1,089226942	+
tetratricopeptide repeat domain 39B	Ttc39b	-1,083655897	+
transient receptor potential cation channel, subfamily M, member 3	Trpm3	-1,080862286	+
nudix (nucleoside diphosphate linked moiety X)-type motif 10 /// nudix (nucleoside diphosphate linked moiety X)-type motif 11	Nudt10 /// Nudt11	-1,078609835	
uromodulin-like 1	Umodl1	-1,077926579	
prolactin receptor	Prlr	-1,074436931	+
peripherin	Prph	-1,070114503	
solute carrier family 24 (sodium/potassium/calcium exchanger), member 2	Slc24a2	-1,067638717	+
SMT3 suppressor of mif two 3 homolog 1 (yeast)	Sumo1	-1,050118553	
histone cluster 3, H2ba /// histone cluster 3, H2bb	Hist3h2ba /// Hist3h2bb	-1,033299651	
leucine rich repeat protein 1, neuronal	Lrrn1	-1,032383005	+
PNMA-like 1 /// coiled-coil domain containing 8	Pnmal1 /// Ccdc8	-1,018206857	
non-metastatic cells 5, protein expressed in (nucleoside-diphosphate kinase)	Nme5	-1,007195501	
immunoglobulin superfamily containing leucine-rich repeat 2	Islr2	-0,996750279	+
a disintegrin-like and metallopeptidase (reprolysin type) with thrombospondin type 1 motif, 5 (aggrecanase-2)	Adamts5	0,937878288	+
RNA binding protein with multiple splicing 2 /// protein kinase, cGMP-dependent, type II	Rbpms2 /// Prkg2	0,946193556	
ISL1 transcription factor, LIM/homeodomain	Isl1	0,964094616	+
hyaluronan synthase 3 /// CTF8, chromosome transmission fidelity factor 8 homolog (S. cerevisiae)	Has3 /// Chtf8	0,970017134	
kinesin family member 26B	Kif26b	0,970299766	+
cache domain containing 1	Cachd1	0,975396633	+
keratin 17	Krt17	0,992804499	
gamma-aminobutyric acid (GABA) A receptor, subunit beta 2	Gabrb2	1,006361865	+
angiopoietin 1	Angpt1	1,010134377	+
R-spondin 2 homolog (Xenopus laevis)	Rspo2	1,010134377	+
GATA binding protein 3	Gata3	1,020340448	+
wingless-related MMTV integration site 5A	Wnt5a	1,03209363	+
quaking	Qk	1,054991672	+
paired-like homeodomain transcription factor 2	Pitx2	1,055591295	+
special AT-rich sequence binding protein 2	Satb2	1,059194282	+
grainyhead-like 3 (Drosophila)	Grhl3	1,068543859	+
transmembrane protease, serine 11b	Tmprss11b	1,073696888	
GLI-Kruppel family member GLI1	Gli1	1,075215994	+
ADAMTS-like 1	Adamtsl1	1,090495686	+

cathepsin A /// phospholipid transfer protein	Ctsa /// Pltp	1,109047529	
RIKEN cDNA A930038C07 gene	A930038C07Rik	1,110292842	+
keratin 14 /// keratin 16 /// keratin 17	Krt14 /// Krt16 /// Krt17	1,135744509	
fermitin family homolog 1 (Drosophila)	Fermt1	1,145605322	+
cytochrome P450, family 26, subfamily b, polypeptide 1	Cyp26b1	1,168446996	+
keratin 6B /// keratin 6A /// keratin 5 /// predicted gene 5478	Krt6b /// Krt6a /// Krt5 /// Gm5478	1,20889443	
homeobox, msh-like 1	Msx1	1,450478679	+
DNA-damage-inducible transcript 4-like	Ddit4l	1,467533417	
forkhead box F2	Foxf2	1,473530495	+
protein phosphatase 4, regulatory subunit 1 /// cadherin 8	Ppp4r1 /// Cdh8	1,481162758	
asporin	Aspn	1,622376462	
expressed sequence AI593442	AI593442	1,709811543	

Supplementary Table XIV Olfactory Receptor genes down-modulated in the *Dlx5-/-* OE vs. wild-type OE, age E12.5, ranked according to log FC (green column).

olfactory receptor 430	Olfr430	-2.63169
olfactory receptor 244	Olfr244	-2.4822
olfactory receptor 550	Olfr550	-2.04848
olfactory receptor 578	Olfr578	-2.04474
olfactory receptor 745	Olfr745	-2.01289
olf rec 69 /// olf rec 68	Olfr69 /// Olfr68	-1.81607
olfactory receptor 15	Olfr15	-1.78614
olfactory receptor 424	Olfr424	-1.72129
olfactory receptor 923	Olfr923	-1.71787
olfactory receptor 385	Olfr385	-1.61259
olfactory receptor 553	Olfr553	-1.44806
olfactory receptor 571	Olfr571	-1.43381
olfactory receptor 1006	Olfr1006	-1.42669
olfactory receptor 1404	Olfr1404	-1.36977
olfactory receptor 691	Olfr691	-1.33891
olfactory receptor 301	Olfr301	-1.33731
olfactory receptor 495	Olfr495	-1.3336
olfactory receptor 410	Olfr410	-1.33354
olfactory receptor 32	Olfr32	-1.32705
olfactory receptor 144	Olfr144	-1.30323
olfactory receptor 918	Olfr918	-1.26796
olfactory receptor 1339	Olfr1339	-1.24233
olfactory receptor 569	Olfr569	-1.23242
olfactory receptor 1176	Olfr1176	-1.23229
olfactory receptor 622	Olfr622	-1.22237
olfactory receptor 545	Olfr545	-1.2194
olfactory receptor 344	Olfr344	-1.21748
olfactory receptor 654	Olfr654	-1.19875
olfactory receptor 1264	Olfr1264	-1.18802
olfactory receptor 507	Olfr507	-1.1606
olfactory receptor 1026	Olfr1026	-1.08461
olfactory receptor 736	Olfr736	-1.06915
olfactory receptor 1044	Olfr1044	-1.05596
olfactory receptor 1342	Olfr1342	-1.02262
olfactory receptor 1408	Olfr1408	-0.99675

Of these 35 OR genes, 4 were also present in the list of 9 genes up-regulated in the wild-type OE during development (Olfr578, Olfr495, Olfr32, Olfr1264) and 4 were present among the 8 up-regulated in the wild-type VNO, during development (Olfr550, Olfr745, Olfr12, Olfr410).

Supplementary Table XV. Top-scoring predicted Dlx5 target genes, based on the Position-Weigth Matrix for this transcription factor.

RefSeq	Gene ID	Symbol	Gene Name	Score
NM_177906	330908	Opcml	opioid binding protein/cell adhesion molecule-like	73
NM_172290	235106	Ntm	neurotrimin	69
NM_001004176	433586	Maml3	mastermind like 3 (Drosophila)	60
NM_026324	67703	Kirrel3	kin of IRRE like 3 (Drosophila)	57
NM_001034013	11418	Accn1	amiloride-sensitive cation channel 1, neuronal (degenerin)	57
NM_021442	14013	Mecom	MDS1 and EVI1 complex locus	56
NM_207676	54725	Cadm1	cell adhesion molecule 1	50
NM_207654	13640	Efna5	ephrin A5	46
NM_030708	80892	Zfhx4	zinc finger homeodomain 4	46
NM_010109	13640	Efna5	ephrin A5	46
NM_010154	13869	Erbb4	v-erb-a erythroblastic leukemia viral oncogene homolog 4 (avian)	44
NM_013646	19883	Rora	RAR-related orphan receptor alpha	41
NM_172577	217593	Slc25a21	solute carrier family 25 (mitochondrial oxodicarboxylate carrier), member 21	38
NM_015820	50787	Hs6st3	heparan sulfate 6-O-sulfotransferase 3	37
NM_080455	228911	Tshz2 t	eashirt zinc finger family member 2	36
NM_001145977	239857	Cadm2	cell adhesion molecule 2	36
NM_172870	242509	Bnc2	basonuclin 2	33
NM_019697	16508	Kcnd2	potassium voltage-gated channel, Shal-related family,	
member 2	33			
NM_177047	319974	Auts2	autism susceptibility candidate 2	32
NM_057173	109594	Lmo1	LIM domain only 1	32
NM_027504	70673	Prdm16	PR domain containing 16	31
NM_019931	56543	Kcnd3	potassium voltage-gated channel, Shal-related family, member 3	31
NM_007496	11906	Zfhx3	zinc finger homeobox 3	31
NM_001177995	70673	Prdm16	PR domain containing 16	31
NM_153131	107448	Unc5a	unc-5 homolog A (C. elegans)	29
NM_133195	108013	Celf4	CUGBP, Elav-like family member 4	29
NM_001177434	545432	Gm13695	predicted gene 13695	28
NM_001177432	668119	Gm13691	predicted gene 13691	28
NM_001177430	668115	Gm13697	predicted gene 13697	28
NM_001177429	668113	Gm13694	predicted gene 13694	28
NM_001177424	668107	Gm13696	predicted gene 13696	28
NM_001177422	668100	Gm13693	predicted gene 13693	28
NM_001177418	668096	Gm13698	predicted gene 13698	28
NM_212435	114142	Foxp2	forkhead box P2	27
NM_025696	66673	Sorcs3	sortilin-related VPS10 domain containing rec 3	27
NM_008032	14266	Aff2	AF4/FMR2 family, member 2	27
NM_001174154	100384868	Pcdha4-g	protocadherin alpha 4-gamma	27
NM_011021	18420	Otp	orthopedia homolog (Drosophila)	26
NM_009472	22253	Unc5c	unc-5 homolog C (C. elegans)	26
NM_001162368	67569	Mgat4c	mannosyl (alpha-1,3-)-glycoprotein beta-1,4-N-	

			acetylglucosaminyltransferase, isozyme C (putative)	26
NM_001113420	30953	Schip1	schwannomin interacting protein 1	26
NM_175548	268890	Lsamp	limbic system-associated membrane protein	25
NM_027398	70357	Kcnip1	Kv channel-interacting protein 1	25
NM_010050	13371	Dio2	deiodinase, iodothyronine, type II	25
NM_007495	11899	Astn1	astrotactin 1	25
NM_001025286	432450	Nkain2	Na+/K+ transporting ATPase interacting 2	25
NM_173446	270028	Fam155a	family with sequence similarity 155, member A	23
NM_026724	68436	Rpl34	ribosomal protein L34	23
NM_010162	14042	Ext1	exostoses (multiple) 1	23
NM_001168321	71458	Bcor	BCL6 interacting corepressor	23
NM_001081300	110796	Tshz1	teashirt zinc finger family member 1	23
NM_183188	268859	Rbfox1	RNA binding protein, fox-1 hom (C. elegans) 1	21
NM_178725	241568	Lrrc4c	leucine rich repeat containing 4C	21
NM_172385	243937	Zfp536	zinc finger protein 536	21
NM_011800	23836	Cdh20	cadherin 20	21
NM_001109661	12014	Bach2	BTB and CNC homology 2	21
NM_008984	19274	Ptprm	protein tyrosine phosphatase, receptor type, M	20
NM_152229	21907	Nr2e1	nuclear receptor subfamily 2, group E, member 1	19
NM_052977	94191	Adarb2	adenosine deaminase, RNA-specific, B2	19
NM_010835	17701	Msx1	homeobox, msh-like 1	19
NM_007831	13176	Dcc	deleted in colorectal carcinoma	19
NM_001113421	30953	Schip1	schwannomin interacting protein 1	19
NM_001004357	66797	Cntnap2	contactin associated protein-like 2	19
NM_199024	319211	Nol4	nucleolar protein 4	18
NM_175481	110637	Grik4	glutamate receptor, ionotropic, kainate 4	18
NM_146188	233107	Kctd15	potassium channel tetram. domain containing 15	18
NM_133256	14843	Gsx2	GS homeobox 2	18
NM_026135	383348	Kctd16	potassium channel tetramerisation domain containing 16	18
NM_021464	19281	Ptprt	protein tyrosine phosphatase, receptor type, T	18
NM_013627	18508	Pax6	paired box gene 6	18
NM_001163328	76897	Ralyl	RALY RNA binding protein-like	18
NM_001159500	26380	Esrrb	estrogen related receptor, beta	18
NM_172298	243931	Tshz3	teashirt zinc finger family member 3	17
NM_145711	252838	Tox	thymocyte selection-associated high mobility group box	17
NM_029609	76429	Lhpp	phospholysine phosphohistidine inorganic pyrophosphate phosphatase	17
NM_015819	50786	Hs6st2	heparan sulfate 6-O-sulfotransferase 2	17
NM_010875	17967	Ncam1	neural cell adhesion molecule 1	17
NM_008734	18183	Nrg3	neuregulin 3	17
NM_007439	11682	Alk	anaplastic lymphoma kinase	17
NM_001190188	18183	Nrg3	neuregulin 3	17
NM_001190187	18183	Nrg3	neuregulin 3	17
NM_001166585	21676	Tead1	TEA domain family member 1	17
NM_001122758	54216	Pcdh7	protocadherin 7	17
NM_001081052	195727	Nhs	Nance-Horan syndrome (human)	17
NM_001001796	107751	Prrxl1	paired related homeobox protein-like 1	17
NM_174988	104010	Cdh22	cadherin 22	16
NM_133442	74053	Grip1	glutamate receptor interacting protein 1	16

NM_021424	58235	Pvrl1	poliovirus receptor-related 1	16
NM_020253	18190	Nrxn2	neurexin II	16
NM_018800	54524	Syt6	synaptotagmin VI	16
NM_011443	20674	Sox2	SRY-box containing gene 2	16
NM_010160	14007	Celf2	CUGBP, Elav-like family member 2	16
NM_001160293	14007	Celf2	CUGBP, Elav-like family member 2	16
NM_001113399	241494	Zfp385b	zinc finger protein 385B	16
NM_001111026	12395	Runx1t1	"runt-related transcription factor 1; translocated to, 1 (cyclin D-related)"	16
NM_001110228	14007	Celf2	CUGBP, Elav-like family member 2	16
NM_001035243	226025	Trpm3	transient receptor potential cation channel, subfamily M, member 3	16
NM_177274	320840	Negr1	neuronal growth regulator 1	15
NM_177259	13131	Dab1	disabled homolog 1 (Drosophila)	15
NM_176835	72778	Dnajc22	DnaJ (Hsp40) homolog, subfamily C, member 22	15
NM_173406	231986	Jazf1	JAZF zinc finger 1 15	
NM_172728	231991	Creb5	cAMP responsive element binding protein 5	15
NM_172671	107515	Lgr4	leucine-rich repeat-containing G protein-coupled receptor 4	15
NM_145841	244431	Sgcz	sarcoglycan zeta	15
NM_053202	108655	Foxp1	forkhead box P1	15
NM_053171	94109	Csmd1	CUB and Sushi multiple domains 1	15
NM_029556	69634	Clybl	citrate lyase beta like	15
NM_022312	21960	Tnr	tenascin R 15	
NM_019944	15285	Mnx1	motor neuron and pancreas homeobox 1	15
NM_019707	12554	Cdh13	cadherin 13	15
NM_011807	23859	Dlg2	discs, large homolog 2 (Drosophila)	15
NM_010053	13390	Dlx1	distal-less homeobox 1	15
NM_010014	13131	Dab1	disabled homolog 1 (Drosophila)	15
NM_007825	13123	Cyp7b1	cytochrome P450, family 7, subfamily b, poly 1	15
NM_007384	11418	Accn1	amiloride-sensitive cation channel 1, neuronal (degenerin)	15
NM_001081377	211712	Pcdh9	protocadherin 9	15
NM_001039094	320840	Negr1	neuronal growth regulator 1	15
NM_207222	109593	Lmo3	LIM domain only 3	14
NM_177879	330222	Sdk1	sidekick homolog 1 (chicken)	14
NM_173868	240690	St18	suppression of tumorigenicity 18	14
NM_130867	170643	Kirrel	kin of IRRE like (Drosophila)	14
NM_029394	69226	Snx24	sorting nexing 24	14
NM_018744	20358	Sema6a	sema domain, transmembrane domain (TM), and cytoplasmic domain, (semaphorin) 6A	14
NM_016694	50873	Park2	Parkinson disease (autosomal recessive, juvenile) 2, parkin	14
NM_009233	20664	Sox1	SRY-box containing gene 1	14
NM_008751	18231	Nxph1	neurexophilin 1	14
NM_008083	14432	Gap43	growth associated protein 43	14
NM_001170985	170643	Kirrel	kin of IRRE like (Drosophila)	14
NM_001122952	18027	Nfia	nuclear factor I/A 14	
NM_001099299	230959	Ajap1	adherens junction associated protein 1	14
NM_001025559	20679	Sox6	SRY-box containing gene 6	14
NM_198671	382034	Gse1	genetic suppressor element 1	13

NM_178870	15478	Hs3st3a1	heparan sulfate (glucosamine) 3-O-sulfotransferase 3A1	13
NM_177708	237847	Rtn4rl1	reticulon 4 receptor-like 1	13
NM_177368	278279	Tmtc2	transmembrane and tetratricopeptide repeat containing 2	13
NM_130865	140477	Dmbx1	diencephalon/mesencephalon homeobox 1	13
NM_030265	80334	Kcnip4	Kv channel interacting protein 4	13
NM_021399	58208	Bcl11b	B-cell leukemia/lymphoma 11B	13
NM_011098	18741	Pitx2	paired-like homeodomain transcription factor 2	13
NM_001114125	69601	Dab2ip	disabled homolog 2 (Drosophila) interacting protein	13
NM_001113198	17342	Mitf	microphthalmia-associated transcription factor	13
NM_001081354	333639	Mamld1	mastermind-like domain containing 1	13
NM_001081097	14807	Grik3	glutamate receptor, ionotropic, kainate 3	13
NM_001079883	58208	Bcl11b	B-cell leukemia/lymphoma 11B	13
NM_001025567	140477	Dmbx1	diencephalon/mesencephalon homeobox 1	13
NM_001011874	497097	Xkr4	X Kell blood group precursor related family member 4	13
NM_181058	56490	Zbtb20	zinc finger and BTB domain containing 20	12
NM_178245	244813	Bsx	brain specific homeobox	12
NM_172804	238266	Syt16	synaptotagmin XVI	12
NM_172522	214058	Megf11	multiple EGF-like-domains 11	12
NM_172475	209630	Frmd4a	FERM domain containing 4A	12
NM_134437	171463	Il17rd	interleukin 17 receptor D	12
NM_033587	93712	Pcdhga4	protocadherin gamma subfamily A, 4	12
NM_033586	93711	Pcdhga3	protocadherin gamma subfamily A, 3	12
NM_033585	93710	Pcdhga2	protocadherin gamma subfamily A, 2	12
NM_033584	93709	Pcdhga1	protocadherin gamma subfamily A, 1	12
NM_033575	93700	Pcdhgb2	protocadherin gamma subfamily B, 2	12
NM_033574	93699	Pcdhgb1	protocadherin gamma subfamily B, 1	12
NM_020296	56878	Rbms1	RNA binding motif, single stranded interacting protein 1	12
NM_019778	56490	Zbtb20	zinc finger and BTB domain containing 20	12
NM_013601	17702	Msx2	homeobox, msh-like 2	12
NM_011265	19726	Rfx3	regulatory factor X, 3 (influences HLA class II expression)	12
NM_010894	18012	Neurod1	neurogenic differentiation 1	12
NM_010710	16870	Lhx2	LIM homeobox protein 2	12
NM_010698	16826	Ldb2	LIM domain binding 2	12
NM_010468	15434	Hoxd3	homeobox D3	12
