# Supplementary figures and images for: Profiling, Bioinformatic, and Functional Data on the Developing Olfactory/GnRH System Reveal Cellular and Molecular Pathways Essential for This Process and Potentially Relevant for the Kallmann Syndrome
Source: Front Endocrinol (Lausanne). 2013 Dec 31;4:203. doi: 10.3389/fendo.2013.00203 (PMC3876029; doi:10.3389/fendo.2013.00203)

# TBP

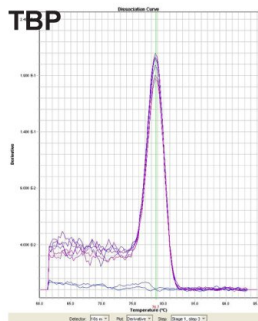

# Akap6

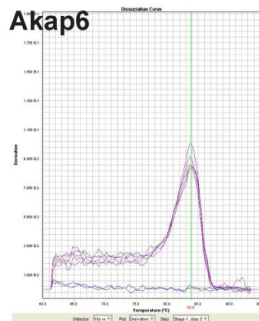

# Lrrn1

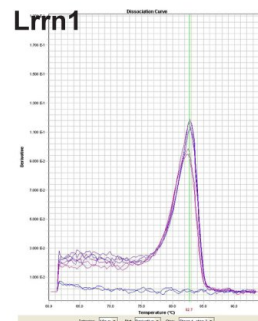

# St8siaVI

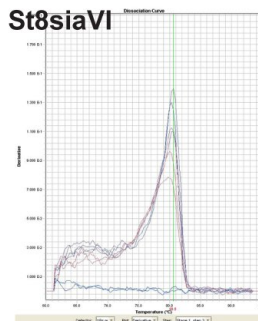

# Lingo2

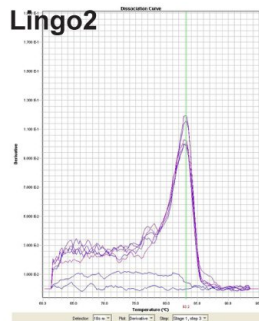

# Homer2

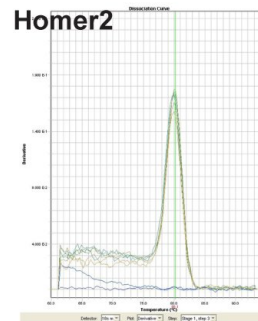

Supplement: Supplementary file 3 [file 76323_Merlo_Presentation3.PDF]
